# The Gender Gap in One-Night Stand Regret: Evidence from Heterosexual and Same-Sex Encounters

**DOI:** 10.1007/s10508-025-03380-3

**Published:** 2026-02-25

**Authors:** Christina Sagioglou, Maximilian Dick

**Affiliations:** Institute of Psychology, University of Innsbruck, Universitätsstraße 15, 6020 Innsbruck, Austria

**Keywords:** One-night stands, Gender differences, Regret, Sexual satisfaction, Decision autonomy, Heteronomy

## Abstract

This research examined gender differences in regret following one-night stands. While women consistently report higher regret than men after casual sexual encounters, the experiential factors contributing to this gap remain untested. We conducted a cross-sectional international survey with 1044 participants (649 females, 395 males; more than 40 nationalities) who retrospectively evaluated their most recent one-night stand. Most participants evaluated their one-night stands neutrally to positively. Crucially, the gender gap in regret emerged predominantly in heterosexual one-night stands, suggesting the phenomenon stems from relational dynamics rather than inherent gender characteristics. Structural equation modeling revealed that sexual satisfaction—particularly orgasm achievement—was the strongest mediator of gender differences, accounting for approximately 49% of the indirect effect. Decision heteronomy, intoxication, and reputational concerns also significantly contributed to the gender gap. Intoxication exhibited a U-shaped relationship with regret, reflecting stronger increases in regret at higher levels of intoxication. Sexual satisfaction strongly mediated gender differences even when controlling for emotional stability and intoxication. Contextual factors (e.g., prior familiarity with the partner, occurrence context) did not notably contribute to regret. The effects were similar for Germans, Austrians, US Americans, and Italians as well as for college students and non-students. The results indicate that enhancing experiential quality through mutual satisfaction and autonomous decision-making offers the most promising path toward enhancing the positive outcomes of single sexual encounters.

**Supplementary Information:**

The online version contains supplementary material available at 10.1007/s10508-025-03380-3.

## Introduction

Casual sexual relationships and experiences (CSREs)—sometimes termed hook-ups—are a common behavior in many countries around the world (Jonason & Balzarini, 2016). They have been investigated predominantly in samples of North American university and college students (Wesche et al., 2021a), who experience casual sex so frequently that CSREs are considered an essential part of college culture (Paul et al., 2000). In recent decades, these encounters have become more accessible to the broader population through online applications specifically designed for finding casual sex partners (e.g., *Kasual, casualx*), highlighting the necessity to investigate them beyond college contexts (Heldman & Wade, 2010). CSREs manifest in diverse forms ranging from one-night stands (ONS) to booty calls and friends-with-benefits relationships. While all share an uncommitted nature (i.e., the casualness), they vary regarding partner relationships and encounter frequency (Claxton & van Dulmen, 2013). For instance, both booty calls and friends with benefits describe casual sex with recurring partners, while implying different levels of closeness. In this research, we investigate the retrospective experiential evaluation of single sexual encounters—ONS—in the context of gender differences.

## Emotional Evaluations of Casual Sexual Relationships and Experiences

Decisions about sexual experiences are among the most common causes of regret in humans (Morrison & Roese, 2011). Accordingly, regret is one of the central emotional consequences investigated in the context of CSREs. Indeed, out of the 34 articles on emotional consequences of CSREs reviewed by Wesche et al. (2021a), 11 (32%) explicitly focused on feelings of regret. This emphasis is logical as regret has been extensively researched in decision-making contexts (Connolly & Zeelenberg, 2002), and engaging in an ONS represents a discrete decision. Regret is typically defined as a negative emotion experienced when individuals realize or believe that the present situation would have been better had they decided or acted differently in the past (Zeelenberg, 1999). It serves to guide future decision making. In the context of ONS, people may engage in counterfactual thinking by comparing the consequences of their decision to engage in the ONS against the alternative of not having done so (Gilovich & Medvec, 1995). As such, post-ONS regret exemplifies action regret: negative feelings about something done rather than something not done (Gilovich & Medvec, 1995).

Various theoretical frameworks offer competing predictions about the emotional consequences of CSREs. On the negative side, these encounters may frustrate attachment needs for connection and belongingness (Fraley & Shaver, 2000), or suffer from insufficient partner communication, creating confusion or uncertainty (Littleton et al., 2009). However, other perspectives suggest potential benefits: CSREs might effectively balance sexual expression with life responsibilities (Shulman & Connolly, 2013), and yield positive outcomes when driven by autonomous rather than external motivations (Deci & Ryan, 2000; Vrangalova, 2015).

Empirical findings largely support theories predicting a positive outcome. Most studies observed low levels of regret, with both men and women evaluating their CSREs positively rather than negatively (for reviews, see Claxton & van Dulmen, 2013; Garcia et al., 2012; Wesche et al., 2021a). This has been confirmed in recent work, where individuals reported overall high levels of positive outcomes such as sexual satisfaction and increased confidence after CSREs (Luz et al., 2022; McKeen et al., 2022) and low levels of negative outcomes such as regret (McKeen et al., 2022). Naturally, this does not mean that CSREs have positive outcomes universally. For the same individual, CSREs can have markedly different effects depending on the practices engaged in (Wesche et al., 2021b). Moreover, although CSREs are evaluated positively overall, when compared with sexual experiences within committed relationships, they are evaluated more negatively—both by the same individual and across individuals (Wesche et al., 2021b).

Most research studying the emotional consequences of CSREs has examined not only regret but also a variety of contextual factors (e.g., alcohol use, partner familiarity), experiential evaluations (e.g., sexual satisfaction, health concern), and demographic variables (e.g., gender) to help explain variation in regret (Wesche et al., 2021a). In the following, we briefly describe a selection of these variables focused on in the present study.

### Gender

Gender is frequently considered as a demographic predictor of sexual regret, most likely because—with a few exceptions—women reliably evaluate casual sex less positively and more negatively than do men (Wesche et al., 2021a). Theoretical explanations of this gender gap involve largely two perspectives. First, evolutionary frameworks such as parental investment theory (Trivers, 1972), sexual strategies theory (Buss & Schmitt, 1993), and error management theory (Haselton & Buss, 2000) suggest that casual sex conflicts with evolved female mating preferences (Bendixen et al., 2017; Galperin et al., 2013; Kennair et al., 2016; Reiber & Garcia, 2010).

Second, sociocultural perspectives emphasize how gender socialization and cultural norms shape asymmetric sexual expectations. Women face harmful sexual double standards that simultaneously stigmatize female sexual agency while celebrating male conquest (Allison & Risman, 2013; England & Bearak, 2014), potentially intensifying post-ONS guilt and regret. Moreover, heteronormative sexual scripts privilege male pleasure, disadvantaging women through gendered communication patterns and sexual practices during casual encounters (Backstrom et al., 2012). While these findings derive predominantly from North American samples (Wesche et al., 2021a), the persistence of gender differences in regret even in Norway’s highly egalitarian, secular society (Kennair et al., 2016, 2018) suggests a complex interplay: evolutionary mechanisms may establish baseline predispositions, while immediate sociocultural contexts shape the specific experiential factors that ultimately predict regret after casual sexual encounters.

### Experiential Factors Predicting Regret

Most extensively, Kennair et al. (2018) examined factors directly related to the experience of the ONS as predictors of regret, comparing Norwegian to US American college students. The present study was designed as an extended replication of their study, and thus we measured the same variables: sexual satisfaction, heteronomy of the decision, health concerns, reputational concern, moral concern, and sexual physical disgust.

#### Sexual Satisfaction

Sexual gratification and pleasure of an ONS reliably predict lower levels of regret (Wesche et al., 2021a). Specifically, high-quality sex in the context of a CSRE was associated with feeling confident, happy, and satisfied, while low-quality sex was linked to feelings of disappointment and embarrassment (Fisher et al., 2012). Some studies find women report greater importance of sexual satisfaction for regret (Kennair et al., 2018), while others find this effect stronger in men (Fisher et al., 2012). Sexual satisfaction in terms of orgasm achievement and overall sexual pleasure of the CSRE is typically higher for men than for women (Kennair et al., 2016, 2018), with the most pronounced effect observed for orgasm achievement (Mahar et al., 2020). Although not directly tested, sexual satisfaction might function as a mediator of gender differences in regret (Kennair et al., 2016). A factor closely related to sexual satisfaction is perceived sexual competence of the partner, with both variables correlating similarly with regret and showing comparable gender differences (Kennair et al., 2016).

#### Heteronomy of the Decision

Based on self-determination theory (Deci & Ryan, 2000), the more intrinsically motivated and autonomous (vs. heteronomous) the decision to engage in the ONS is, the lower feelings of regret should be. For example, feminist theories argue that defying gender scripts by engaging in an ONS signals high autonomy and may lead to positive outcomes (Kalish & Kimmel, 2011). Accordingly, factors diminishing autonomy should increase regret. Indeed, heteronomous motivations to engage in casual sex predicted negative health outcomes at a later point in time (Vangralova, 2015). Feeling pressured also predicts stronger levels of regret, slightly more for women than for men (Kennair et al., 2018). Finally, a factor directly influencing the autonomy of the decision is alcohol use. When alcohol is consumed before the ONS, alcohol-induced decision making may be an important component of decision autonomy.

#### Health, Reputational, and Moral Concerns

Compared to sex with committed partners, ONS carry a higher risk of STI and unplanned pregnancies due to contextual factors such as spontaneity, intoxication, and lower levels of monogamy (Downing-Matibag & Geisinger, 2009; Manning et al., 2005). Having unprotected sex was sometimes associated with more negative outcomes of CSREs (e.g., Napper et al., 2016; but see Zimmer-Gembeck et al., 2015, who found no correlation between condom use and emotional outcomes), and frequently cited as a reason for regret (Galperin et al., 2013). Recent studies have combined these health concerns with reputational concern into a composite worry score, which predictably correlates positively with regret (Kennair et al., 2018), though reputational concern appears to be the primary driver of this relationship (Kennair et al., 2016). However, these concern variables did not substantially reduce the gender gap in regret.

Reputational concern measures the evaluation of the ONS in terms of others’ perceptions. This item captures societal and subcultural value systems as well as considerations specific to the person’s social network. For the present population, a recent study with both German students and a community sample indicated that moderate levels of casual sexual activity were rated most favorably for both genders, suggesting at least a baseline level of social acceptance of these behaviors (Weber & Friese, 2024; see also Allison & Risman, 2013). Yet, the trajectory differs by gender: men are permitted a higher number of casual sex partners before their reputation suffers. Reflecting this common societal double standard, reputational concern could partially explain the gender gap in regret.

Finally, individuals may have moral concerns about their ONS, which strongly relates to regret (Kennair et al., 2018). When asked about reasons for regret, many individuals report feeling guilty and having cheated on a partner (e.g., Fisher et al., 2012; Galperin et al., 2013). Unlike reputational concern, the perceived wrongness of the ONS reflects personal moral evaluations, capturing diverse forms of harm the ONS may have caused. Kennair et al. (2018) found moral concern to be a strong predictor of regret for both men and women, yet slightly stronger for women.

#### Physical Disgust

Kennair et al. (2018) assessed how physically disgusting and unhygienic the ONS was, but combined these items as part of a composite score together with moral concern. While they reported that—out of the three items—moral concern was the strongest predictor of regret, they did not analyse their specific individual contributions. Since moral concern and physical disgust (e.g., unhygienic) represent conceptually distinct constructs (e.g., Herz & Hinds, 2013), we examined them separately. To our knowledge and based on a recent review (Wesche et al., 2021a), no other research has measured physical disgust in the context of CSREs and gender. While the presence of disgust should correlate with regret, we expect overall levels to be low. Additionally, although women self-report higher disgust sensitivity, their physiological reactions suggest no reliable gender difference in actual disgust responses (Rohrmann et al., 2008). In sum, we expect this measure to be neither a strong predictor of regret nor a meaningful explanation for gender differences.

### Contextual Factors Predicting Regret

Various contextual factors may influence regret about an ONS. For example, regret can be affected by the specific sexual behaviors engaged in (Wesche et al., 2021b), by being in a relationship and thus cheating (Fisher et al., 2012), or by it being one’s first sexual experience (Fisher et al., 2012; Galperin et al., 2013). Age at the time of the ONS is another reliable correlate of regret, with older participants experiencing less regret (Kennair et al., 2016; Mendle et al., 2013). Below, we report details on selected contextual factors investigated in the present study, which constitute the primary extensions to this replication.

#### One-Night Stand Type

In a sample of 6955 US adults, women reported lower levels of sexual satisfaction after casual sex with women compared to casual sex with men and to men in both types of casual sex encounters, while men in homosexual casual encounters reported the highest level of sexual satisfaction (Mark et al., 2015). Conversely, another study with 24,230 US adults found lesbian (*n* = 136) and bisexual (*n* = 376) women reported lower levels of regret than heterosexual women, though both groups reported more regret than men (Galperin et al., 2013). It remains unclear how heterosexual compared to same-sex ONS affect regret eight years later in a largely European sample, following significant progression in attitudes and legal frameworks regarding homosexuality. Furthermore, various experiential factors of same-sex ONS have never been measured in relation to regret, a gap which we intend to address with our study.

#### Intoxication

Alcohol consumption significantly increases CSRE likelihood (Owen et al., 2010) and is prevalent during casual sexual encounters, especially among young adults (Claxton et al., 2015; Fisher et al., 2012). Alcohol use also positively predicts regrettable experiences (Jones et al., 2020). However, the relationship between alcohol and post-ONS evaluation reveals complex patterns. Research findings are inconsistent—some studies report minimal impact on emotional outcomes (Owen et al., 2010; Wesche et al., 2021b), while others document substantial negative consequences including diminished positive affect, heightened negative affect (Lewis et al., 2012), and increased anxiety (Black et al., 2019). When directly asked about sources of regret, both men and women frequently identify substance use as a primary factor (Fisher et al., 2012). Pharmacologically, heavy alcohol consumption impairs sexual functioning and arousal across genders (Peugh & Belenko, 2001), suggesting a dose-dependent relationship between intoxication and negative ONS evaluation, with potentially stronger effects at higher consumption levels.

The influence of non-alcoholic psychoactive substances on post-ONS regret remains largely unexplored, despite occasional inclusion in general substance use assessments (Fielder & Carey, 2010; Fisher et al., 2012). Psychoactive substances (i.e., psychoactive drugs) are chemical compounds which alter central nervous system functioning and affect perception, mood, consciousness, cognition, and behavior (World Health Organization [WHO], 2004). They vary substantially in prevalence, legality, and subjective effects. Despite their predominantly illicit status, usage rates remain significant in many populations (Global Drug Survey, 2021; WHO, 2004). Given their diverse psychopharmacological properties and experiential effects, we sought to explore their distinct contributions to post-ONS evaluations.

#### Relationship with the One-Night Stand Partner

Relationship with the partner can be considered in terms of quantity (i.e., familiarity) and in terms of quality (i.e., the type of relationship). While some argue that familiarity is typically low in ONS (Claxton & van Dulmen, 2013), we maintain that familiarity can vary substantially, even in single sexual encounters. Prior research often did not differentiate between types of casual sex when investigating familiarity, yet people may have sex only once with someone they know well, such as a friend, classmate, or colleague. Within the context of CSREs, familiarity can have a detrimental impact, such as when it negatively affects an existing, continuing relationship or involves an ex-partner (Fisher et al., 2012; Galperin et al., 2013). Conversely, engaging in casual sex with someone barely known can also induce regret (Fisher et al., 2012; Galperin et al., 2013), and across CSREs, familiarity generally improves the experience (Wesche et al., 2021a). For example, more than 20% of men and women reported that ruining a friendship caused their regret, while approximately 45% of women and 35% of men cited hardly knowing their partner as a source of regret (Fisher et al., 2012). For ONS specifically, familiarity alone may not strongly predict regret, but the particular relationship type with the partner likely plays a more significant role.

### Personality Predictors of Regret

We are not aware of research that has investigated how personality relates to the experience of post-CSRE regret. Low emotional stability and agreeableness predict counterfactual thinking (Bacon et al., 2020), suggesting they should similarly predict regret after an ONS. Women typically score lower on emotional stability (Feingold, 1994), yet there are no gender differences in regret and counterfactual thinking generally (Roese & Summerville, 2005). We will explore personality as a predictor of post-ONS regret and as a potential covariate of gender differences in regret.

### The Present Research

This research is an extended replication of the study by Kennair et al. (2018), who compared gender differences in evaluations of the most recent ONS in Norway to the USA. Women consistently reported higher levels of regret after casual sexual encounters than men across Norwegian and US American samples. The six explanatory factors investigated—sexual gratification, worry, disgust, partner’s sexual competence, feeling pressured, and taking initiative—predicted regret differentially by gender. All predictors except worry had stronger effects on women’s regret than men’s. The combined factors explained approximately 40% of the variance in regret. While this moderation approach effectively demonstrated how various factors differently influence men’s and women’s experiences of casual sex regret, it stopped short of quantifying the extent to which these factors account for the overall gender difference in regret.

In the present study, we changed the analysis approach to statistically compare the experiential factors as mediators of gender differences, directly testing for indirect effects—which has, to our knowledge, not been examined yet. That is, gender served as an exogenous independent variable, regret as the dependent variable, and the experiential factors as mediators. We refined the measurement of regret in three key ways: (1) using multiple items rather than a single item; (2) implementing a 5-point Likert scale rather than a 4-point ordinal scale, allowing more nuanced expression and approximating interval-level measurement; and (3) incorporating both regret-gladness and shame-pride dimensions, capturing a broader emotional manifestation of regret.

A second extension was testing ONS type as a moderator. Despite examining large samples of similar US adult populations, past research has yielded inconsistent findings with regard to regret and sexual satisfaction after CSREs (Galperin et al., 2013; Mark et al., 2015). Recent social progress in Central Europe, including the legalization of same-sex marriage in 18 European countries since 2013 (e.g., Germany in 2017; Austria in 2019), should reduce moral and reputational concerns surrounding same-sex ONS due to shifting societal attitudes toward homosexuality. More importantly, examining gender differences as a function of ONS type can reveal crucial insights about the mechanisms driving regret. If the gender gap disappears in same-sex ONS, this would suggest that evolutionary female characteristics are less likely to explain the regret gap. Alternatively, if regret stems primarily from specific relational dynamics between men and women, then the gender gap should vary depending on ONS type.

Finally, we added psychoactive substance use as a predictor variable, thereby combining much prior research with Kennair et al.’s (2018) extensive list of experiential factors. In addition to testing it as a direct predictor of regret, we planned to test alcohol intoxication as a first-order mediator influencing both sexual satisfaction and decision heteronomy (Herbenick et al., 2019). Moreover, because a) alcohol intoxication increases the likelihood of engaging in an ONS and b) women are less inclined to engage in an ONS per se (Grøntvedt et al., 2015; Kennair et al., 2018), we expect women in heterosexual ONS to be more intoxicated when making the decision to engage in the ONS than men. As such, intoxication may also function as a mediator of gender differences in regret.

## Method

### Participants and Procedure

We recruited a large, international convenience sample. In order to minimize the influence of response tendencies and arrive at a clear picture of the hypothesised relationships, we asked only about the most recent ONS (Galperin et al., 2013; Kennair et al., 2018). We advertised this study by asking whether participants had “experience with one-night stands (single sexual encounters)” and, in case they did, invited them to participate in an exciting study which would take 5 min of their time (see SI for the complete recruitment texts on differing platforms). All measures were taken at one point in time, making this a cross-sectional design. The original questionnaire, data set, and analysis code are accessible here: https://osf.io/9rk7y/.

Invitations to participate in the study were posted in various thematically-related online forums and sent out via the university mailing list. There was no compensation for this study. In total, 1079 participants reported having had at least one ONS. Although we explicitly addressed people with ONS experience, our initial filter showed that 131 had not yet had the opportunity to experience an ONS, and 199 reported having had the opportunity, but not making use of it. After the exclusion of those without experience and a few implausible cases (n = 4; see “documentation” file in the online repository), the sample included 1075 individuals (651 females, 398 males, 16 non-binary, 10 did not indicate; *M*_Age_ = 25.14, SD = 6.45, range = 16–61). As there were not enough non-binary participants and partners to reliably consider this variable in our analysis, we created a subsample of only female and male participants and partners (*n* = 1044, 649 females, 395 males, *M*_Age_ = 25.01, SD = 6.41, range = 16–61). The most common nationalities were German (*n* = 349), followed by Austrian (*n* = 348), US American (*n* = 119), Italian (*n* = 107), and British (*n* = 31). The survey was available in German (*n* = 815) and English (*n* = 229). Further participant demographics are given in Table 1.

**Table 1:** Sociodemographic characteristics of the sample

	Women		Men	
	*n*	%	*n*	%
Education				
Middle school or earlier	2	0	6	2
High school or equivalency	37	6	19	5
Junior college/trade school	320	49	177	44
4-year college/university	213	33	127	32
Graduate degree—Masters	71	11	60	15
Graduate degree—Doctoral	6	1	9	2
Marital status				
Not married	601	92	366	92
Married	36	6	28	7
Separated	5	1	2	1
Divorced	6	1	2	1
Widowed	2	0	0	0
Relationship status				
Single	348	54	215	54
In a relationship	300	46	183	46
Occupation				
At work as employee or employer/self-employed	146	22	134	34
Employed, on child care leave	6	1	2	1
Employed, on other special leave (e.g., sickness; not holiday)	3	0	2	1
Unemployed less than 12 months	12	2	11	3
Unemployed 12 months or more	3	0	2	1
Unable to work due to long-term illness or disability	3	0	1	0
Retired	0	0	1	0
Full-time homemaker/fulfilling domestic tasks	5	1	2	1
In education (at school, university, etc.)	441	68	226	57
Other (please specify:)	31	5	16	4
ONS partner gender				
Female	28	4	348	87
Male	620	95	47	12
Non-binary	0	0	2	1
I don't know	0	0	1	0
Student				
Yes	474	73.1	256	64.8
No	174	26.9	139	35.2
Time passed since ONS (grouped)				
< 1 month	80	17.4	47	14.2
1–3 month	51	11.1	45	13.6
3–12 month	148	32.2	89	27
1–3 years	181	39.3	112	34
> 3 years	128	27.8	78	23.6

### Measures

After providing informed consent, we specified that “This survey investigates feelings and experiences concerning single sexual encounters (one-night stand; henceforth: ONS).” Participants first indicated how many ONS they had had in total (*M* = 7.76, SD = 21.56; *Median* = 3, range = 1–561, skew = 17.6, kurtosis = 421.18). As this response was highly skewed and had some extreme outliers, we log-transformed it. We then asked participants to recall their most recent ONS, provide its date of occurrence, and then answer all questions concerning that specific ONS.

#### Contextual Variables

Participants indicated their relationship status at the time (single, in a relationship, recently broken up, almost broken up), the sexual activities they engaged in (given oral sex, received oral sex, vaginal sex, anal sex, other), details regarding the occurrence (e.g., after a date, after a party, after a small social gathering, after a sex date, after a random encounter, other), about the relationship with the partner (e.g., met for the first time on the same day/evening, dating partner, distant acquaintance, good friend, ex-partner, …), and the partner’s gender (female, male, non-binary, unknown). From the date of occurrence and participants’ age, we calculated the age at the time of the ONS. From the date of occurrence and the survey response date, we calculated the days passed since the ONS. This variable was log-transformed because of very positive skew, high kurtosis, and large range (see SI, Figures S1-S2).

#### Influence of Psychoactive Substances

To measure subjective intoxication, we combined information from two questions. First, participants reported whether they were under the influence of substances when deciding to engage in the ONS (yes/no). If yes, they rated the intensity of this influence on a 5-point scale (1—*barely noticeable* to 5—*very strong*). We transformed these responses into a 6-point scale where 1 = no substances consumed and 2–6 represented increasing levels of influence intensity.

We then averaged this 6-point scale with participants’ ratings of how much their decision was influenced by substances (1—*influenced by psychoactive substances [e.g., alcohol]*, 5—*not at all influenced by psychoactive substances [e.g., alcohol];* reverse-scored). This created a composite subjective intoxication score with very good reliability (average split-half reliability = 0.88; *M* = 3.10, SD = 1.53).

Examination of this variable revealed a non-normal, multimodal distribution. While Hartigans’ (Hartigan & Hartigan, 1985) dip test for unimodality was significant, *D* = 0.09, *p* < 0.001, a mixture model analysis suggested 9 components, hinting at a complex distribution with multiple peaks. We analyzed the relationship to regret in detail, which we report in the results section.

To assess the potential influence of different drugs in more detail, participants also indicated the strength of the influence of common other psychoactive substances (e.g., cannabis, MDMA; 1—*did not consume*, 6—*very strong*). Yet, these were too infrequently reported to allow for a comparative analysis.

#### Retrospective Evaluation of Participants’ Most Recent ONS

The next part of the questionnaire dealt with the subjective experience of the ONS. Most of the items were taken from Kennair et al. (2018), but for theoretical and data-driven reasons, we created the mean scores differently, which we describe below. First, feelings of regret were assessed with three items asking about the extent of felt regret (1—*no regret at all*; 5—*very strong regret*), regret versus gratification (1—*I regret it very much*; 3—*neutral*; 5—*I’m glad I did it,* reverse-coded), and feelings of shame versus pride (1—*strong feelings of shame*; 3—*neutral*; 5—*great pride,* reverse-coded). Higher values indicate higher levels of regret (*α* = 0.82; *M* = 2.50, SD = 0.91).

Sexual satisfaction was assessed with five items by asking about the extent to which participants experienced sexual pleasure (1—*not at all*, 5—*very strongly*), whether they had had an orgasm (*yes/no*), the subjective importance of an orgasm (1—*not important at all*, 5—*very important*), perceived sexual competence of the partner (1—*not at all*; 5—*very much*), and the partner’s ability to sufficiently satisfy them sexually (1—*not at all*; 5—*very much*). Examining the correlation of these variables showed that importance to have an orgasm was only very weakly related to the other four variables (*r* = 0.04–0.14; and *r* = 0.32 with having had an orgasm or not), and reliability analyses suggested that the alpha was raised from 0.77 to 0.84 by dropping the importance from the scale. Furthermore, we had reservations about the theoretical alignment of this variable with the overall sexual satisfaction concept. We thus created a mean score based on the four variables except importance, after rescaling the binary orgasm item into a 5-point item (*α* = 0.84,* M* = 3.25, SD = 0.97), assigning the value “5” to *yes*, and “1” to *no*. We rescaled the orgasm item instead of standardizing each item because this approach preserves the intuitive 1–5 metric while treating orgasm as spanning the full satisfaction range. We also conducted analyses using the more common approach of standardizing items prior to creating composite scores (z-transformation), which yielded near-identical results.

Next, we examined worry to have become pregnant or having caused a pregnancy (*M* = 1.83, SD = 1.23), worry to have contracted a sexually transmitted infection (STI; *M* = 2.01, SD = 1.14), and reputational concerns (*M* = 1.71, SD = 1.13). Combining the items to one mean score as done in previous research (Kennair et al., 2018) resulted in an unacceptably low reliability of *α* = 0.50 (similar to what was reported by Kennair et al., 2018), so we included them as separate variables. We also measured moral concern with one item (“To what extent was the ONS wrong/immoral?”; *M* = 1.72, SD = 1.16). If the ONS was perceived as wrong/immoral, participants were able to select potential reasons. Options were “wrong person (e.g., good friend, ex-partner of a friend, own ex-partner)”, “cheating (on your part),” “I hurt somebody else by doing it,” and “I hurt myself by doing it”. Participants again had the option to provide more details about their reasons by selecting “Other”.

We measured physical disgust with two items (“To what extent was the ONS disgusting; unhygienic?”; 1—*not at all*; 5—*very much;* average split half reliability = 0.69; *M* = 1.46, SD = 0.74).

At last, heteronomy of the decision to engage in the ONS was examined with five items. Participants rated the extent to which they felt pressured to engage in the ONS, felt obliged to engage in the ONS (1—*not at all*, 5—*very strongly*), whether the decision was well-considered or impulsive (1—*well-considered*; 5—*very impulsive*), whether it was one’s own decision (1—*my own decision*; 5—*not really my own decision*), and who took the initiative to engage in the ONS (1—*my partner*; 2—*largely my partner*, 3—*both of us even-handedly*; 4—*Largely I*, 5—*myself*; reverse-coded). We created a mean score so that higher values indicated a more heteronomous decision (*α* = 0.73; *M* = 2.35, SD = 0.71).

#### Personality and Demographics

The last section of the survey assessed Big-Five personality dimensions with the Ten-Item-Personality Inventory (Gosling et al., 2003), which measures each trait with two adjective pairs, one of which is phrased in reverse. For example, extraversion is assessed with these two pairs: I am… “extraverted, enthusiastic”, “reserved, quiet” (response scale: 1—*Not at all*, 7—*Completely*). Average split-half reliabilities were: extraversion = 0.76 (*M* = 4.53, SD = 1.50), agreeableness = 0.39 (*M* = 5.29, SD = 1.15), conscientiousness = 0.69 (*M* = 5.10, SD = 1.35), emotional stability = 0.71 (*M* = 4.87, SD = 1.42), openness to experience = 0.43 (*M* = 5.49, SD = 1.42). The distribution of personality differed expectedly by gender, but not notably by ONS type or the interaction (see SI, e.g., Figure S4).

Participants also indicated demographic data such as age, gender, nationality, relationship, and marital status, educational attainment, and occupation status. There were four nationalities that had more than 100 cases. To explore nationality as a predictor of regret, we created a five-categorical variable, ordered by frequency (German [reference], Austrian, Italian, US American, other).

### Data Analysis

First, we examined the distribution patterns of regret and all potential predictor variables, as well as zero-order correlations among them. We then analyzed the relationship between contextual variables (e.g., relationship status, partner familiarity, occurrence context) and regret through multiple regression. Given the non-normal distribution of subjective intoxication, we compared multiple functional (e.g., linear, quadratic) forms to determine its optimal relationship with regret.

Our main analysis began with a linear regression model predicting regret from participant gender, ONS type, and their interaction, followed by a sensitivity power analysis. We also examined the distribution for heterogeneity. To investigate mechanisms underlying gender differences in regret, we conducted a series of structural equation models (SEM). Our initial model tested parallel mediation with sexual satisfaction, decision heteronomy, intoxication (both linear and quadratic terms), and reputational concerns as mediators between gender, ONS type, their interaction, and regret. We calculated conditional indirect effects for heterosexual and same-sex ONS separately, with indices of moderated mediation quantifying differences between ONS types.

We extended this analysis in several ways. First, we added emotional stability as a covariate to test whether personality traits accounted for gender differences. Second, we tested a serial mediation model where gender influenced intoxication, which then affected decision heteronomy and satisfaction, which in turn influenced regret—as outlined in the introduction. Third, we decomposed the satisfaction composite into its components (pleasure, orgasm achievement, partner competence, and partner’s ability to satisfy) to identify which aspects most strongly mediated gender differences. Following up on this model, we analyzed sexual practices in relation to regret and satisfaction.

Finally, we explored several potential moderators: (1) intoxication as a moderator of gender and ONS type effects on regret; (2) student status as a moderator to test whether findings differed between student and non-student populations; and (3) nationality as a moderator to examine potential country differences in the gender gap.

Our sample comprised 620 women reporting ONS with men, 28 women reporting ONS with women, 348 men reporting ONS with women, and 47 men reporting ONS with men. Due to this uneven distribution and partly small cell sizes, we employed robust estimation methods throughout our analyses. We tested data for heteroscedasticity, which confirmed significant variance heterogeneity across groups. Consequently, we implemented heteroscedasticity-consistent standard errors (HC3) in regression analyses to obtain reliable parameter estimates. For structural equation models, we used maximum likelihood estimation with robust standard errors (MLR) and handled missing data through full information maximum likelihood (FIML). For inference on indirect effects, we implemented nonparametric bootstrapping with 5000 resamples to derive bias-corrected 95% confidence intervals. In all analyses involving multiple statistical tests, we controlled for family-wise error rate using Holm-adjusted *p*-values to minimize Type I errors. Notably, we did not apply further corrections for multiple testing in our SEM analyses, as we included only mediators that showed significant correlations with regret and gender in our Holm-corrected correlation analyses, thus filtering variables before inclusion in the more complex models. All data were analyzed using RStudio 2024.12.1 Build 563, “Kousa Dogwood” Release (27,771,613, 2025-02-02) for windows based on R Version 4.4.3 (2025-02-28 ucrt) “Trophy Case” (R Core Team, 2021; RStudio Team, 2021).

## Results

### Contextual Variables

**Fig. 1:**
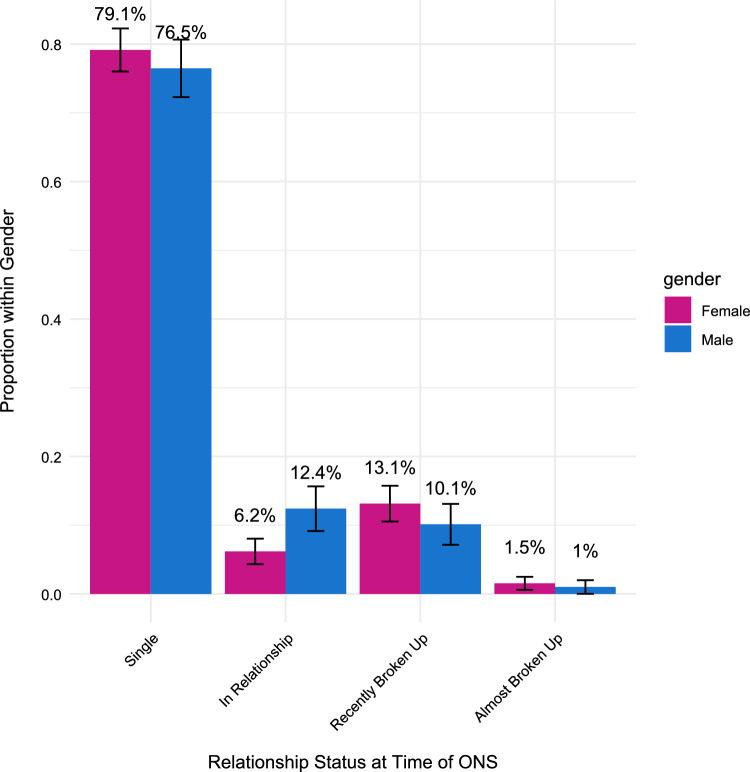
Distribution of each gender across relationship statuses. Bars represent the proportion of women and men who were in each relationship status category when engaging in their most recent ONS. Error bars show 95% confidence intervals. Statistical significance was assessed using Fisher’s exact tests for each relationship category with Holm correction for multiple comparisons. There was a significant gender difference only for the “In Relationship” category (*p* = 0.003), with men significantly more likely than women to report having a one-night stand while in a relationship. No significant gender differences were found for the other categories (Single: *p* = 0.633; Recently Broken Up:* p* = 0.507; Almost Broken Up: *p* = 0.633). Both genders predominantly reported being single at the time of their one-night stand, though proportionally more so for women than men

Table S1 reports a variety of contextual variables by gender and partner gender. The large majority of individuals (78%) were single at the time of the ONS, while 13% were just or almost broken up and 8.5% were in a relationship. These are much higher percentages of individuals being single than those reported in recent studies with comparable populations (Kennair et al., 2016, 2018). There were notable gender differences regarding whether a person was in a relationship (Fig. 1), with men being more likely to be in a relationship than women (see Figure S5, for the distribution of relationship status by gender and ONS type).

More than half of the sample (55%) had met their partner that day, 34% reported to have known them for a while (e.g., long-term but distant acquaintance: 19.8%, friend: 9.8%, ex-partner: 0.8%, colleague: 0.7%), and 11.3% had met recently (e.g., dating partner: 9.1%). This shows that single sexual encounters most often (64% of the time) occur with strangers or brief acquaintances (Claxton & van Dulmen, 2013), while about one fifth of the sample had loosely known the person for a while (see Figure S6, for the distribution of partner familiarity by gender and ONS type).

Most individuals (50%) reported having had three ONS or fewer, with 20% reporting having had ten or more. For some individuals, their latest ONS had happened more than a year ago (Table 1). We thus investigated the effects of time passed since the most recent ONS and report these results in detail in the supplementary section and return to them in the general discussion. Most often (60%), the ONS happened after a party or a smaller social gathering, followed by a date (15%), a date for sex (13%), and a chance encounter (8%). On average, people were 23 years old at their latest ONS (SD = 5.27, *Median* = 21.85, range = 13.77–59.03 years old; *n* = 1026), with 90 individuals younger than 18, of which 11 were younger than 16. Context differed depending on gender and ONS type (see Figure S7).

We also examined the correlations (Holm-corrected, bootstrap [*n* = 5000] derived CIs, accounting for different variable types) between contextual variables and evaluations of the ONS. The strongest correlation in our dataset was between sex date contexts and intoxication levels (*r*_linear_ = − 0.36, 95% CI [− 0.41, − 0.31], *p* < .001; *r*_quadratic_ = 0.16, 95% CI [0.10, 0.22], *p* < 0.001), indicating substantially lower intoxication during planned sexual encounters compared to other contexts. Sex dates were also associated with lower decision heteronomy (*r* = − 0.13, 95% CI [− 0.19, − 0.07], *p* = 0.003). Regular date contexts showed similar but weaker patterns, with lower intoxication levels (*r*_linear_ = − 0.20, 95% CI [− 0.26, − 0.14], *p* < 0.001) compared to other contexts. Both date and sex date contexts were more likely to occur with slightly familiar partners.

Age at the time of the ONS emerged as another noteworthy correlate. Older participants reported significantly lower intoxication levels (*r* = − 0.25, 95% CI [− 0.30, − 0.19], *p* < 0.001), lower decision heteronomy (*r* = − 0.21, 95% CI [− 0.27, − 0.15], *p* < 0.001), and higher sexual satisfaction (*r* = 0.20, 95% CI [0.14, 0.26], *p* < 0.001). Importantly, increased age was associated with reduced regret (*r* = − 0.14, 95% CI [− 0.20, − 0.08], *p* = 0.001) and fewer reputational concerns (*r* = − 0.12, 95% CI [− 0.18, − 0.06], *p* = 0.019). Age and ONS experience were positively correlated (*r* = 0.31, 95% CI [0.25, 0.37], *p* < 0.001), suggesting accumulated experience with age.

Individuals in relationships reported higher moral concerns (*r* = 0.22, 95% CI [0.16, 0.27], *p* < 0.001), yet, they also reported higher sexual satisfaction (*r* = 0.14, 95% CI [0.08, 0.20], *p* = 0.002). Relationship status was positively associated with age (*r* = 0.23, 95% CI [0.17, 0.29], *p* < 0.001) and ONS experience (*r* = 0.11, 95% CI [0.05, 0.17], *p* = 0.038), suggesting that ONS while in committed relationships may occur more frequently among older, more experienced individuals. The complete correlation results are reported in Table S2.

To examine the overall influence of contextual variables on regret, we calculated a linear regression model. We used treatment-coding when predictors were multi-categorical (occurrence context, reference: party; relationship status, reference: single; partner familiarity, reference: not known). We predicted regret with occurrence context, relationship status, the age at the time of the ONS, the number of ONS (log-transformed), the partner familiarity, and the days that passed since the event (log-transformed). The overall regression model was significant, adjusted *R*^2^ = 0.05, *F*(13, 1006) = 5.00, *p* < 0.001^1^. After accounting for multiple testing (Holm), there were two significant predictors of regret in the contextual model. Regret after a party was significantly higher than after a sex date, *b* = 0.32, SE = 0.09, *t* = 3.49, *p* = 0.007, and people who have experienced more ONS in their lives report lower levels of regret, *b* = − 0.12, SE = 0.04, *t* = 3.37, *p* = 0.011. We provide correlational analyses of context variables with experiential variables in the supplementary file (e.g., Table S2).

### Intoxication

As intoxication had a non-normal, multimodal distribution, we first examined it separately. The majority (75%) reported being under the influence of psychoactive substances when making the decision to engage in the ONS. Among these individuals, the most frequently consumed substance was alcohol, which was consumed by 99%. In addition, some individuals had consumed cannabis (17%), cocaine (3%), MDMA (2%), and amphetamines (1%). Only 12 individuals reported not consuming alcohol but a different substance. Table S3 breaks down drug use and intensity by participant gender and ONS partner gender.

**Fig. 2:**
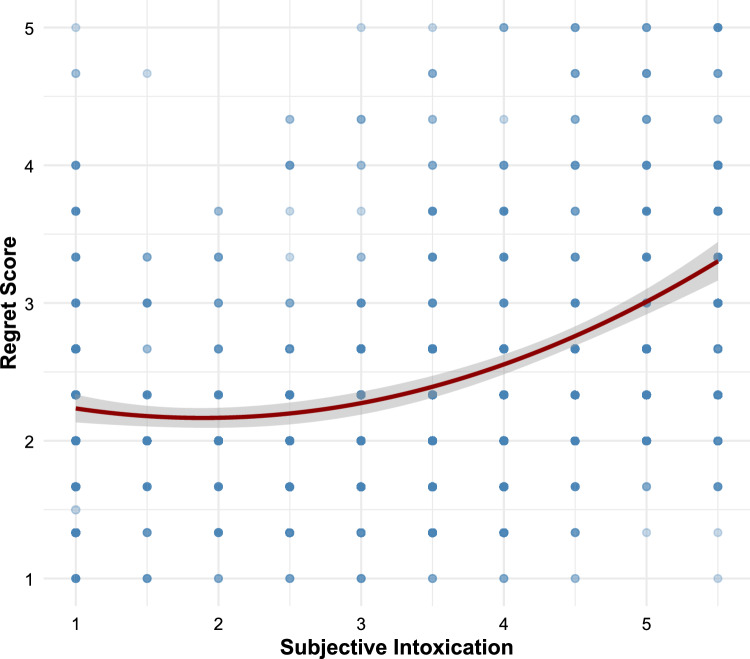
Relationship between subjective intoxication and regret following ONS. The scatter plot shows individual observations (*n* = 1037) with the fitted quadratic curve (dark red line) and 95% confidence interval (shaded area). The positive linear and quadratic terms create an accelerating pattern where regret increases more steeply at higher levels of intoxication

To determine the optimal functional form for modeling the relationship between subjective intoxication and regret, we compared multiple regression models using information criteria and explained variance. Model comparison revealed that a quadratic function provided the best fit (Table S4), significantly outperforming linear and logarithmic models, while more complex functions (cubic, spline) offered no meaningful improvement. In a multiple regression model predicting regret with the linear and quadratic intoxication term, there was a significant positive linear term (*b* = 0.21, *p* < 0.001) and a significant positive quadratic term (*b* = 0.09, *p* < 0.001), resulting in a U-shaped, upward-curving relationship (Fig. 2). This nonlinear pattern explained approximately 14.4% of the variance in regret scores, *F*(2, 1034) = 86.8, *p* < 0.001). It suggests that regret increases with intoxication levels at an accelerating rate. While even low levels of intoxication are associated with some increase in regret, the effect becomes notably stronger at higher intoxication levels. Regression analyses involving subjective intoxication thus include the linear and the squared term as predictors.

### Evaluation of the One-Night Stand

This sample reported rather low levels of regret, evident in the fact that most values cluster below the midpoint of 3 (*Median* = 2.33). The typical post-ONS feelings for the vast majority of our sample (79%) were thus neutral to positive. More precisely, the descriptive analysis of the three regret items revealed generally positive evaluations of the ONS. For direct regret intensity (*M* = 2.06, SD = 1.25; *Median* = 2), nearly half of participants (47.4%) reported no regret at all. The regret versus gratification item (*M* = 3.54, SD = 1.16; *Median* = 3) showed a more balanced distribution with a slight tendency toward positive evaluation, as 48.7% of participants reported being glad about their ONS experience (ratings of 4–5). Feelings of shame versus pride (*M* = 3.01, SD = 0.78; *Median* = 3) predominantly clustered around the neutral midpoint, with 63.5% of participants selecting this option. We visualize the results of the distribution analysis of the regret items and all other evaluation items in the supplementary materials (Figures S8–S11).

The distribution of the other evaluation items is consistent with the overall low levels of regret. As a result, some variables had floor effects. Most notably, direct pressure to engage in the ONS had the strongest floor effects (confirming prior studies, e.g., Garcia & Reiber, 2008), similar to physical disgust. As the skewness in negative evaluation items is conceptually meaningful and not excessive, while the composite scores have good distributional properties, we use the mean scores as described in the method section. The sexual satisfaction items—the only positively framed experiential items—are overall normally distributed with slight positive skew, which again reflects the rather positive overall evaluation of the ONS (Figures S10–S11).

### Evaluation of the One-Night Stand as a Function of Participant Gender and One‑Night Stand Type

**Fig. 3:**
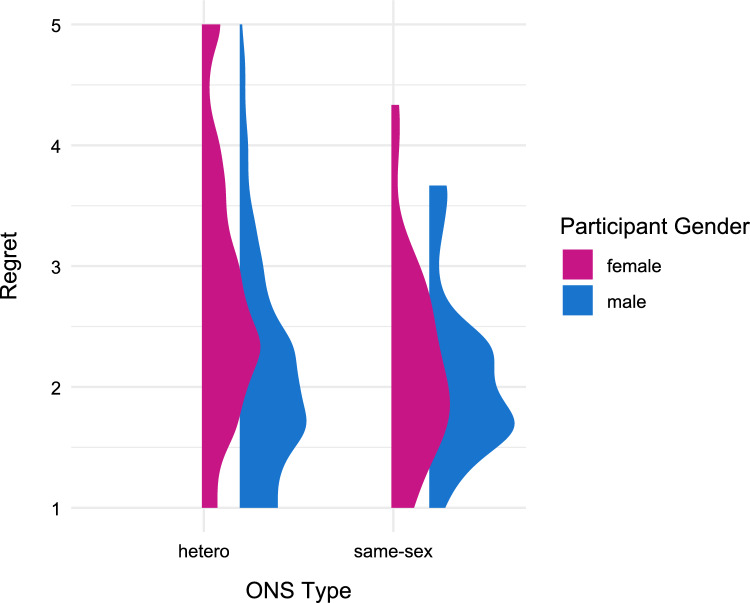
Distribution density of regret by participant gender and ONS type. This graph displays the distribution of retrospective regret about the most recent casual sexual encounters by gender and ONS type. Higher values indicate more regret. Due to the small number of people in same-sex ONS (n = 75), these results are to be interpreted with caution with regard to reliability

Due to the uneven group sizes, we tested our data for heteroscedasticity and examined the comparative density distribution (Fig. 3). We employed both formal tests and visual inspection of residual plots (Figure S14). The Breusch-Pagan test (BP = 8.93, *df* = 3, *p* = 0.030) and White’s test (*χ*^2^ = 8.50, *df* = 1*, p* = 0.004) both indicated significant heteroscedasticity in the model. Additionally, Levene’s test for homogeneity of variance across groups revealed significant differences in variance among the gender and ONS type combinations, *F*(3, 1033) = 4.04, *p* = 0.007. We therefore implement heteroscedasticity-consistent standard errors (HC3) in the subsequent analyses to obtain more reliable parameter estimates (MacKinnon & White, 1985).

**Fig. 4:**
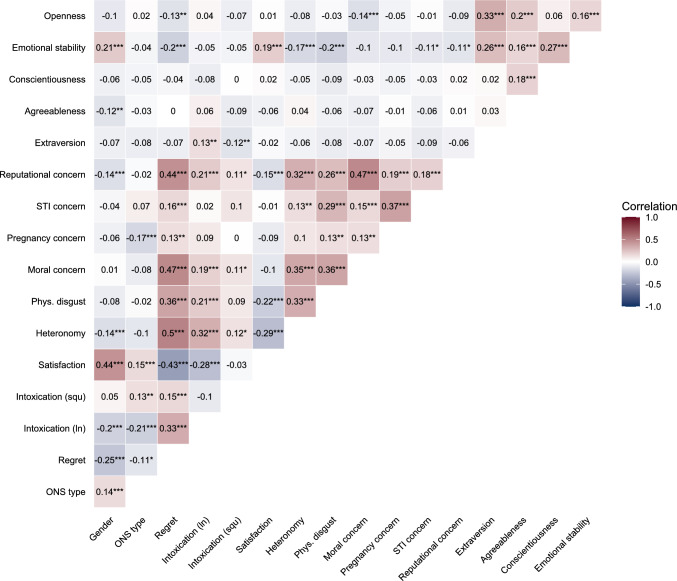
Correlation matrix of predictors and outcome variables. Pearson correlation coefficients are displayed with significance levels indicated by asterisks (*p < 0.05, **p < 0.01, ***p < 0.001, after Holm correction). Color intensity represents correlation strength, with red indicating positive correlations and blue indicating negative correlations. Gender was coded female = − 1, male = 1, ONS type was coded heterosexual = − 1, same-sex = 1

We calculated a linear regression model predicting regret with participant gender, ONS type (female coded as − 1, male coded as 1; heterosexual coded as − 1, same-sex as 1), and their interaction (which equals the effect of partner gender). The overall regression model was significant, *R*^2^ = 0.07, *F*(3, 1040) = 26.03, *p* < 0.001. There was a significant main effect of participant gender indicating that women reported more regret than did men, *b* = − 0.14, SE = 0.05, *t* = − 2.97, *p* = 0.003, *β* = − 0.16 [− 0.27, − 0.04]. There was also a significant main effect of ONS type indicating that regret was higher after heterosexual ONS than after same-sex ONS, *b* = − 0.17, SE = 0.05, *t* = − 3.48, *p* = 0.001, *β* = − 0.17 [− 0.30, − 0.07]. The effect of the interaction (i.e., of partner gender) was not significant, *b* = 0.09, *p* = 0.063, *β* = 0.10 [− 0.02, 0.22] (Fig. 4).

**Fig. 5:**
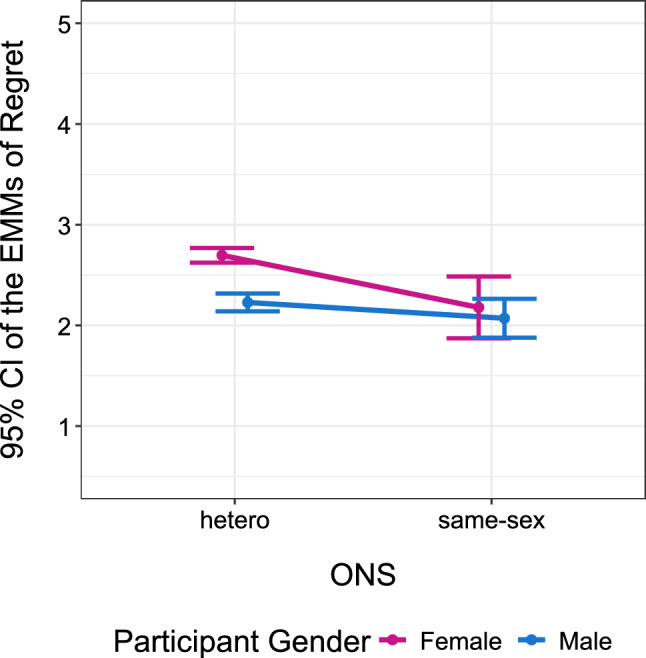
Regret as a function of gender and ONS type. Retrospective evaluation of the ONS predicted by participant gender and ONS type. Higher values indicate more regret. Error bars indicate the 95% confidence interval of the robust estimated marginal means

Examining the contrasts (Tukey-adjusted p-values, robust standard errors) showed that women after heterosexual ONS reported significantly more regret (EMM = 2.70, SE = 0.04) than all other groups (Fig. 5). That is, more than men after heterosexual ONS (EMM = 2.23, SE = 0.05), *b* = 0.47, SE = 0.06, *t* = 8.02, *p* < 0.001, *d* = 0.53 [0.47, 0.59], more than women after same-sex ONS (EMM = 2.18, SE = 0.16), *b* = 0.52, SE = 0.16, *t* = 3.22, *p* = 0.007, *d* = 0.59 [0.51, 0.66], and more than men after same-sex ONS (EMM = 2.07, SE = 0.10), *b* = 0.63, SE = 0.11, *t* = 5.95, *p* < 0.001, *d* = 0.71 [0.62, 0.79]. None of the other groups differed from each other (*p*s > 0.467, *d*s < 0.16). All following analyses are based on this interaction model.

#### Sensitivity Power Analysis

A sensitivity analysis was conducted to evaluate the statistical power of our basic regression model. With a sample size of 1044 participants and alpha set at 0.05, the study had 80% power to detect effects as small as Cohen's *f*^2^ = 0.0076 (partial *R*^2^ = 0.0075). The observed robust effect sizes for the main effects of gender (*f*^2^ = 0.0085, partial *R*^2^ = 0.0084) and ONS type (*f*^2^ = 0.0116, partial *R*^2^ = 0.0115) exceeded this threshold, indicating adequate power for detecting these effects. However, the interaction effect between sex and ONS type yielded a smaller effect size (*f*^2^ = 0.0033, partial *R*^2^ = 0.0033), falling below the minimum detectable effect size threshold. This indicates that the study was underpowered to reliably detect the interaction effect, and the non-significant interaction result should be interpreted with caution as a potential Type II error.

#### Examining Heterogeneity in Gender Effects

To examine individual-level heterogeneity beyond mean differences, we calculated distributional overlap statistics. For heterosexual one-night stands, 69.9% of women reported regret levels exceeding the median man, while 71.6% of men scored below the median woman. The probability of superiority indicated that a randomly selected woman had a 60.3% probability of reporting higher regret than a randomly selected man. Cliff’s Delta—a non-parametric effect size appropriate for ordinal data, which directly quantifies the probability of dominance between groups (Cliff, 1993)—indicated a small-to-medium effect size for the gender difference, *δ* = 0.31, 95% CI [0.24, 0.38]. In contrast, for same-sex one-night stands, the gender difference was substantially attenuated: only 42.9% of women exceeded the median man’s regret score, 44.7% of men fell below the median woman, and the probability of superiority approached chance levels (46.8%). Cliff’s Delta indicated a negligible effect size for the gender difference, *δ* = 0.08, 95% CI [− 0.20, 0.34].

These estimates suggest considerable within-gender variability in regret responses, with approximately 30% of women in heterosexual encounters reporting regret levels at or below the responses by men, while same-sex encounters showed near-complete distributional overlap between genders.

### Why Do Women Retrospectively Evaluate Heterosexual One-Night Stand Less Positively Than Other Gender-One‑Night Stand Type Combinations?

To investigate the gender gap established above, we first examined the zero-order correlations of the proposed mediating variables with regret and gender (Fig. 4; see Table S5, for correlation coefficients including confidence intervals; and Figure S12). Correlations of the mediating variables with regret by ONS type are reported in Table S6. Correlations on item level are reported in Figure S13.

Subjective intoxication, decision heteronomy, moral concerns, reputation concerns, feeling physically disgusted, and STI concern were significantly positively correlated with regret, while sexual satisfaction, emotional stability, and openness to experience were negatively correlated with regret. Intoxication, decision heteronomy, reputational concerns, agreeableness, and emotional stability were higher for women than for men, while sexual satisfaction was lower for women than for men. We then calculated a series of structural equation models (SEM) exploring variables that were correlated with both gender and regret (intoxication, satisfaction, heteronomy, reputational concerns) as mediators, starting with a parallel mediated moderation model. As moral concern was highly correlated with regret but did not differ by gender, we report an analysis of its distribution and the reasons indicated for moral concern in the supplementary materials (Figures S15–S17, Table S7).

#### Structural Equation Modeling: Parallel Mediation of the Basic Interaction Model

We constructed an SEM to test a mediated moderation model using lavaan (Rosseel, 2012) with participant gender, ONS type, and their interaction as exogenous predictors. The model included sexual satisfaction, decision heteronomy, intoxication, and reputational concerns as mediators, with regret as the outcome variable. To address the previously identified U-shaped relationship between intoxication and regret, we mean-centered the intoxication variable and included both linear and quadratic terms as separate mediators in the model.

Given that the model was fully saturated (*df* = 0), fit indices showed perfect fit (*χ*^2^ = 0.00, *df* = 0; CFI = 1.00; TLI = 1.00; SRMR = 0.00; RMSEA = 0.00, 90% CI [0.00, 0.00]). Robust versions of these indices showed identical results (Robust CFI = 1.00; Robust TLI = 1.00; Robust RMSEA = 0.00). Information criteria values for this model were AIC = 18,286.32 and BIC = 18,509.11. The model explained about 44% (*R*^2^) of the variance in regret (calculated by subtracting the residual variance of regret from 1; i.e., *R*^2^ = 1 − 0.558822 = 0.441178). Path statistics are reported in Table 2.

**Table 2:** Path statistics for the parallel structural equation model

Criterion	Predictor	*b*	SE	*z*	*p*	LLCI	ULCI	*β*
Regret	Gender	− 0.02	0.04	− 0.44	0.660	− 0.09	0.06	− 0.02
Regret	ONS type	− 0.05	0.04	− 1.31	0.190	− 0.13	0.03	− 0.03
Regret	Gender × ONS type	0.02	0.04	0.40	0.686	− 0.06	0.09	0.02
Satisfaction	Gender	0.38	0.06	6.25	< 0.001	0.26	0.50	0.34
Satisfaction	ONS type	0.21	0.06	3.31	0.001	0.09	0.33	0.10
Satisfaction	Gender × ONS type	− 0.11	0.06	− 1.88	0.061	− 0.23	0.01	− 0.10
Heteronomy	Gender	− 0.03	0.04	− 0.95	0.340	− 0.10	0.04	− 0.05
Heteronomy	ONS type	− 0.13	0.04	− 3.75	< 0.001	− 0.20	− 0.06	− 0.10
Heteronomy	Gender × ONS type	0.07	0.04	1.99	0.047	0.00	0.14	0.10
Intoxication (ln)	Gender	− 0.46	0.09	− 5.15	< 0.001	− 0.63	− 0.29	− 0.29
Intoxication (ln)	ONS type	− 0.50	0.09	− 5.66	< 0.001	− 0.67	− 0.33	− 0.17
Intoxication (ln)	Gender × ONS type	− 0.22	0.09	− 2.46	0.014	− 0.39	− 0.04	− 0.14
Intoxication (squ)	Gender	0.28	0.12	2.43	0.015	0.05	0.51	0.14
Intoxication (squ)	ONS type	0.40	0.11	3.54	< 0.001	0.18	0.63	0.11
Intoxication (squ)	Gender × ONS type	0.26	0.12	2.27	0.023	0.04	0.49	0.13
Reputational concern	Gender	− 0.06	0.07	− 0.89	0.372	− 0.19	0.07	− 0.05
Reputational concern	ONS type	− 0.03	0.07	− 0.50	0.617	− 0.16	0.10	− 0.02
Reputational concern	Gender × ONS type	0.12	0.06	1.86	0.063	− 0.01	0.25	0.10
Regret	Satisfaction	− 0.22	0.02	− 8.94	< 0.001	− 0.26	− 0.17	− 0.26
Regret	Heteronomy	0.37	0.04	9.52	< 0.001	0.29	0.45	0.29
Regret	Intoxication (ln)	0.07	0.02	4.23	< 0.001	0.04	0.10	0.11
Regret	Intoxication (squ)	0.04	0.01	3.54	< 0.001	0.02	0.07	0.09
Regret	Reputational concern	0.22	0.02	9.10	< 0.001	0.17	0.27	0.27

Pathway	*b*	SE	z	*p*	LLCI	ULCI	*β*
Indirect: Gender→Satisfaction→Regret (Hetero)	− 0.11	0.01	− 7.53	< 0.001	− 0.14	− 0.08	− 0.11
Indirect: Gender→Heteronomy→Regret (Hetero)	− 0.04	0.01	− 4.28	< 0.001	− 0.06	− 0.02	− 0.04
Indirect: Gender→Intoxication (ln)→Regret (Hetero)	− 0.02	0.01	− 3.14	0.002	− 0.03	− 0.01	− 0.02
Indirect: Gender→Intoxication (squ)→Regret (Hetero)	0.00	0.00	0.24	0.807	− 0.01	0.01	0.00
Indirect: Gender→Reputational concern→Regret (Hetero)	− 0.04	0.01	− 4.64	< 0.001	− 0.06	− 0.02	− 0.04
Indirect: Gender→Satisfaction→Regret (Same− sex)	− 0.06	0.03	− 2.18	0.029	− 0.11	− 0.01	− 0.06
Indirect: Gender→Heteronomy→Regret (Same− sex)	0.01	0.03	0.55	0.584	− 0.04	0.06	0.01
Indirect: Gender→Intoxication (ln)→Regret (Same-sex)	− 0.05	0.02	− 2.86	0.004	− 0.08	− 0.01	− 0.05
Indirect: Gender→Intoxication (squ)→Regret (Same-sex)	0.02	0.01	1.97	0.049	0.00	0.05	0.03
Indirect: Gender→Reputational concern→Regret (Same-sex)	0.01	0.03	0.50	0.619	− 0.04	0.07	0.01
Total indirect effect (Hetero)	− 0.20	0.02	− 9.42	< 0.001	− 0.24	− 0.16	− 0.21
Total indirect effect (Same-sex)	− 0.05	0.06	− 0.89	0.375	− 0.17	0.06	− 0.06
Total effect (Hetero)	− 0.23	0.03	− 8.00	< 0.001	− 0.29	− 0.18	− 0.25
Total effect (Same-sex)	− 0.05	0.09	− 0.59	0.554	− 0.23	0.12	− 0.06
Moderated mediation: Satisfaction pathway	0.05	0.03	1.83	0.068	0.00	0.10	0.05
Moderated mediation: Heteronomy pathway	0.05	0.03	1.94	0.053	0.00	0.11	0.06
Moderated mediation: Intoxication (ln) pathway	− 0.03	0.01	− 2.09	0.037	− 0.06	0.00	− 0.03
Moderated mediation: Intoxication (squ) pathway	0.02	0.01	1.87	0.061	0.00	0.05	0.02
Moderated mediation: Reputational concern pathway	0.05	0.03	1.85	0.064	0.00	0.11	0.06
Moderated mediation: Total index	0.15	0.06	2.44	0.015	0.03	0.27	0.16

*LLCI* Lower level confidence interval, *ULCI* Upper level confidence interval; all confidence intervals of the pathways are 95% confidence intervals based on 5000 bias-corrected bootstraps. Hetero refers to heterosexual one-night stands, Same-sex refers to same-sex one-night stands. Gender was coded as − 1 = female, 1 = male; ONS type was coded as − 1 = heterosexual, 1 = same-sex

After accounting for the mediators, the direct effects of gender (*b* = − 0.02, *p* = 0.660), ONS type (*b* = − 0.05, *p* = 0.181), and their interaction (*b* = 0.02, *p* = 0.685) on regret were not significant. Men reported significantly higher sexual satisfaction than women (*b* = 0.38, *p* < 0.001) and less intoxication (linear component: *b* = − 0.46, *p* < 0.001; quadratic component: *b* = 0.28, *p* = 0.013). Gender was not significantly associated with decision heteronomy (*b* = − 0.03, *p* = 0.333) or reputational concerns (*b* = − 0.06, *p* = 0.375), suggesting that the zero-order correlations were accounted for by other variables in this model (i.e., covariances with other mediators).

**Fig. 6:**
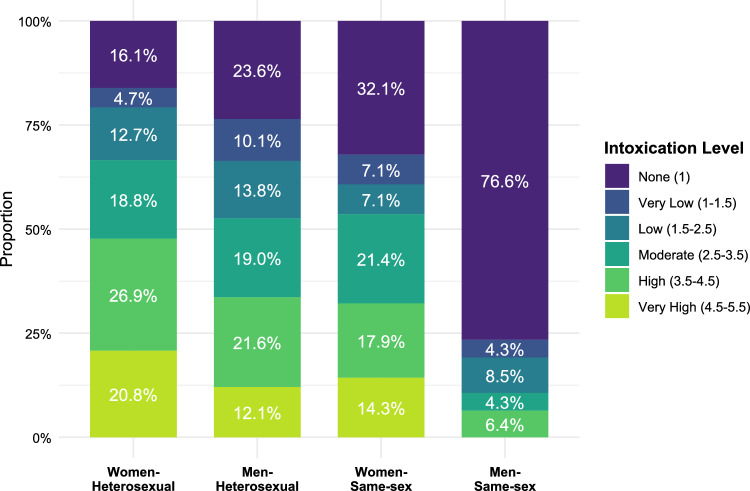
Intoxication levels during ONS by gender and ONS type. Most men in same-sex ONS were not intoxicated at all (36 out of 47 individuals). This mirrors the finding that ONS for this group occur in different contexts; specifically, 49% of the time after sex dates, possibly explaining why intoxication is infrequent (see also Figure S7, Table S1)

ONS type significantly predicted all mediators except reputational concerns. Same-sex ONS were associated with higher satisfaction (*b* = 0.21, *p* < 0.001), lower heteronomy (*b* = − 0.13, *p* < 0.001), lower linear intoxication (*b* = − 0.50, *p* < 0.001), and higher quadratic intoxication (*b* = 0.40, *p* < 0.001). Due to the substantial differences in intoxication, we followed up on this pattern and visualized intoxication by gender and ONS type (Fig. 6).

Significant yet very small gender × ONS type interactions were observed in heteronomy and both intoxication variables, indicating that gender differences varied by ONS type. All mediator variables significantly predicted regret. Sexual satisfaction predicted lower regret (*b* = − 0.22, *p* < 0.001). Heteronomy (*b* = 0.37, *p* < 0.001), linear intoxication (*b* = 0.07, *p* < 0.001), quadratic intoxication (*b* = 0.04, *p* < 0.001), and reputational concerns (*b* = 0.22, *p* < 0.001) predicted higher regret.

Indirect Effects and Indices of Moderated Mediation.

In heterosexual ONS, we found significant indirect effects of gender on regret through sexual satisfaction, *b* = − 0.11, 95% CI [− 0.14, − 0.08], decision heteronomy, *b* = − 0.04, 95% CI [− 0.06, − 0.02], linear intoxication, *b* = − 0.02, 95% CI [− 0.03, − 0.01], and reputational concerns, *b* = − 0.04, 95% CI [− 0.06, − 0.02]. The quadratic intoxication coefficient was not significant, *b* = 0.00, 95% CI [− 0.01, 0.01]. The total indirect effect for heterosexual ONS was *b* = − 0.20, 95% CI [− 0.25, − 0.16], with a total effect of *b* = − 0.23, 95% CI [− 0.29, − 0.18] and a nonsignificant direct effect of *b* = − 0.02, 95% CI [− 0.09, 0.06], suggesting full mediation. Together, the mediators explain most of the gender difference by accounting for 86% of the total effect. Based on the standardized estimates (Table 2), satisfaction contributes 46% of the total effect, heteronomy and reputational concern each account for 17%, and linear intoxication for 7%.

In same-sex ONS, the total effect (*b* = − 0.05, 95% CI [− 0.23, 0.12]) and the total indirect effect (*b* = − 0.05, 95% CI [− 0.17, 0.06]) were not significant, which is to be expected given the non-existent gender difference in same-sex ONS in the baseline model. The indirect effect was significant via sexual satisfaction,* b* = − 0.06, 95% CI [− 0.11, − 0.01], linear intoxication: *b* = − 0.05, 95% CI [− 0.08, − 0.02]; quadratic intoxication: *b* = 0.02, 95% CI [0.01, 0.05]. Overall, the pattern is complex with some suppressor effects and likely unreliable given the small sample size.

The indices of moderated mediation were significant for decision heteronomy,* b* = 0.05, 95% CI [0.001, 0.11], linear intoxication, *b* = − 0.03, 95% CI [− 0.06, − 0.01], and quadratic intoxication, *b* = 0.02, 95% CI [0.003, 0.05]. The total index of moderated mediation was significant, *b* = 0.15, 95% CI [0.03, 0.27], confirming that ONS type significantly moderated the indirect effects of gender on regret.

#### Parallel Structural Equation Modeling with Emotional Stability as a Covariate

Emotional stability was correlated with being male and lower levels of regret, and so we added it as a covariate. This extended model included direct paths from emotional stability to regret and to all mediators (satisfaction, decision heteronomy, intoxication, and reputational concern). It showed expected excellent model fit due to full saturation (CFI = 1.00, TLI = 1.00, SRMR = 0.00), and accounted for 44.3% of the variance in regret (full model statistics in Table S8). Emotional stability significantly predicted regret (*b* = − 0.04, *p* = .024, 95% CI [− 0.07, − 0.01]), with more emotionally stable individuals reporting less regret. Emotional stability was positively associated with sexual satisfaction (*b* = 0.09, *p* < 0.001, 95% CI [0.04, 0.13]) and negatively associated with heteronomous decision-making (*b* = − 0.08, *p* < 0.001, 95% CI [− 0.11, − 0.04]) and reputational concerns (*b* = − 0.07, *p* = 0.004, 95% CI [− 0.12, − 0.02]).

Notably, after controlling for emotional stability, the direct effect of gender on regret decreased more (*b* = − 0.01, *p* = 0.778) compared to the original model without emotional stability (*b* = − 0.03). However, gender continued to significantly predict sexual satisfaction (*b* = 0.36, *p* < 0.001, 95% CI [0.24, 0.48]) and intoxication (linear: *b* = − 0.45, *p* < 0.001, 95% CI [− 0.62, − 0.29]; quadratic: *b* = 0.30, *p* = 0.008, 95% CI [0.08, 0.52]). The total effect of gender on regret in heterosexual ONS remained significant (*b* = − 0.20, 95% CI [− 0.26, − 0.14]), though slightly reduced from the original model (*b* = − 0.23).

The indirect effects through the other mediators in heterosexual ONS remained significant: satisfaction (*b* = − 0.10, 95% CI [− 0.13, − 0.07]), decision heteronomy (*b* = − 0.03, 95% CI [− 0.05, − 0.01]), linear intoxication (*b* = − 0.02, 95% CI [− 0.03, − 0.01]), and reputational concerns (*b* = − 0.03, 95% CI [− 0.05, − 0.02]). The total indirect effect in heterosexual ONS (*b* = − 0.18, 95% CI [− 0.22, − 0.14]) was slightly reduced compared to the original model (*b* = − 0.20).

Similarly, the moderated mediation index remained significant (*b* = 0.14, 95% CI [0.02, 0.25]), indicating that the pathways explaining gender differences vary in heterosexual versus same-sex ONS, also after controlling for emotional stability. In same-sex ONS, the total effect of gender remained non-significant (*b* = − 0.04, 95% CI [− 0.21, 0.14]), similar to the original model (*b* = − 0.05). These findings suggest that while emotional stability partially explains the gender gap in regret after casual sexual encounters, significant indirect effects persist through satisfaction, decision-making, and intoxication pathways, particularly in heterosexual ONS. Adding emotional stability to the model increased the explained variance only minimally by 0.3% from 44 to 44.3%.

#### Alternative Structural Equation Modeling: Serial Mediation Model

We additionally examined a serial mediation model where gender influences intoxication, which then affects decision heteronomy and satisfaction, which in turn influence regret (full model statistics in Table S9). The model further controlled for quadratic effects of intoxication and included reputational concerns as a parallel mediator. Model fit was mixed, with some indices very good, others inadequate (robust where applicable): CFI = 0.96, TLI = 0.56, RMSEA = 0.14 [0.10, 0.17], SRMR = 0.04. The prior, parallel mediation model demonstrated significantly better fit than the serial mediation model based on a scaled chi-square difference test (χ^2^_diff_ = 51.24, *df* = 3, *p* < 0.001). Nevertheless, we examined the indirect paths to test whether intoxication functions as a first-order mediator, contributing to regret indirectly via lowering sexual pleasure and decision autonomy.

Regarding first-stage effects, the analysis revealed a significant effect of gender on intoxication levels, *b* = − 0.46, *p* < 0.001, 95% CI [− 0.63, − 0.29], with men reporting lower levels of intoxication than women. Additionally, there was a significant effect of ONS type on intoxication, *b* = − 0.50, *p* < 0.001, 95% CI [− 0.67, − 0.33], with lower intoxication levels reported during same-sex ONS. The gender by ONS type interaction was also significant, *b* = − 0.22, *p* = 0.010, 95% CI [− 0.38, − 0.05], indicating that the gender difference in intoxication was more pronounced in same-sex ONS.

Regarding second-stage effects, intoxication significantly predicted both decision heteronomy, *b* = 0.12, *p* < 0.001, 95% CI [0.09, 0.15], and sexual satisfaction, *b* = − 0.14, *p* < 0.001, 95% CI [− 0.18, − 0.10]. The quadratic term of intoxication was also significant in both equations (heteronomy: *b* = 0.05, *p* < 0.001, 95% CI [0.02, 0.07]; satisfaction: *b* = − 0.04, *p* = 0.018, 95% CI [− 0.07, − 0.01]), suggesting non-linear relationships similar to that with regret.

After controlling for intoxication, gender remained a significant predictor of sexual satisfaction, *b* = 0.33, *p* < 0.001, 95% CI [0.21, 0.45], with men reporting higher satisfaction, but not of decision heteronomy, *b* = 0.01, *p* = 0.758, 95% CI [− 0.06, 0.07]—as in the parallel model. The gender by ONS type interaction significantly predicted both heteronomy, *b* = 0.09, *p* = 0.009, 95% CI [0.02, 0.15], and satisfaction, *b* = − 0.13, *p* = 0.024, 95% CI [− 0.25, − 0.02].

Regarding effects on regret, both mediators significantly predicted regret in the expected directions: sexual satisfaction negatively, *b* = − 0.22, *p* < 0.001, 95% CI [− 0.26, − 0.17] and decision heteronomy positively, *b* = 0.37, *p* < 0.001, 95% CI [0.29, 0.45]. Intoxication maintained a direct positive effect on regret, *b* = 0.07, *p* < 0.001, 95% CI [0.03, 0.10], as did its quadratic term, *b* = 0.04, *p* < 0.001, 95% CI [0.02, 0.07]. Reputational concern also significantly predicted higher regret, *b* = 0.22, *p* < 0.001, 95% CI [0.18, 0.27]. After accounting for all mediating pathways, the direct effects of gender, *b* = − 0.02, *p* = 0.658, 95% CI [− 0.09, 0.06], ONS type, *b* = − 0.05, *p* = 0.173, 95% CI [− 0.13, 0.02], and their interaction, *b* = 0.02, *p* = 0.682, 95% CI [− 0.06, 0.09], on regret were not significant.

##### Indirect Effects and Indices of Moderated Mediation

All four serial indirect effects were significant. For heterosexual ONS, the serial indirect effects of gender on regret via intoxication and heteronomy, *b* = − 0.01, 95% CI [− 0.02, − 0.01], and via intoxication and satisfaction, *b* = − 0.01, 95% CI [− 0.05, − 0.004], were significant. Similarly, for same-sex ONS, both serial pathways were significant: via intoxication and heteronomy, *b* = − 0.03, 95% CI [− 0.05, − 0.02] and via intoxication and satisfaction, *b* = − 0.02, 95% CI [− 0.04, − 0.01]. These findings support the hypothesis that gender influences regret through a sequential process involving intoxication and decision/satisfaction factors.

The strongest direct mediational pathway for heterosexual ONS was through sexual satisfaction, *b* = − 0.10, 95% CI [− 0.13, − 0.08], followed by reputational concerns, *b* = − 0.04, 95% CI [− 0.06, − 0.02], decision heteronomy, *b* = − 0.03, 95% CI [− 0.05, − 0.01], and linear intoxication effects, *b* = − 0.02, 95% CI [− 0.03, − 0.01].

For same-sex ONS, only the direct intoxication pathway was significant, *b* = − 0.05, 95% CI [− 0.08, − 0.02], while pathways through satisfaction, *b* = − 0.04, 95% CI [− 0.10, 0.01], heteronomy, *b* = 0.04, 95% CI [− 0.01, 0.08], and reputational concerns, *b* = 0.01, 95% CI [− 0.05, 0.06] were not significant—as in the parallel model.

The total indirect effect of gender on regret was significant for heterosexual ONS, *b* = − 0.20, 95% CI [− 0.25, − 0.16], but not for same-sex ONS, *b* = − 0.09, 95% CI [− 0.21, 0.03]. Correspondingly, the total effect of gender on regret was significant for heterosexual ONS, *b* = − 0.24, 95% CI [− 0.29, − 0.18], but not for same-sex ONS (*b* = − 0.09, 95% CI [− 0.27, 0.09]).

The total moderated mediation index was not significant, *b* = 0.11, 95% CI [− 0.01, 0.24]. All but one of the individual indices of moderated mediation were significant: the serial pathways through intoxication and heteronomy, *b* = − 0.02, 95% CI [− 0.04, − 0.00], through intoxication and satisfaction, *b* = − 0.01, 95% CI [− 0.03, − 0.00], the direct intoxication pathway, *b* = − 0.03, 95% CI [− 0.06, − 0.01], the heteronomy pathway, *b* = 0.06, 95% CI [0.01, 0.11], and the satisfaction pathway, *b* = 0.06, 95% CI [0.01, 0.11]. The reputational concerns pathway did not show significant moderation, *b* = 0.05, 95% CI [− 0.01, 0.11].

##### Summary

The model explained 42% of the variance in regret, slightly less than the parallel model, while still indicating substantial explanatory power. For heterosexual ONS, indirect effects collectively accounted for 86.4% of the total gender effect on regret, with the direct satisfaction pathway explaining the largest portion (43%) of this mediated effect. By comparison, the serial pathways via intoxication and satisfaction (6.4%) and via intoxication and heteronomy (5.4%) accounted for smaller but significant proportions, suggesting that while the intoxication-mediated processes are statistically meaningful, direct gender differences in sexual satisfaction represent the dominant mechanism underlying the gender gap in casual sex regret.

#### Structural Equation Modeling: Satisfaction Component Model

Sexual satisfaction was a strong correlate of gender and regret and a strong mediator in both the parallel and the serial mediation model. To examine which specific aspects of sexual satisfaction most strongly mediate gender differences in regret, we developed a more detailed SEM that decomposed the composite satisfaction measure into its constituent components. This model maintained the same basic structure as our primary parallel mediation model but replaced the overall satisfaction variable with four individual predictors: sexual pleasure, orgasm achievement, perceived partner competence, and partner’s ability to satisfy, while retaining all other mediators (decision heteronomy, intoxication, and reputational concerns). We employed a diagonally weighted least squares (DWLS) estimator to appropriately handle the binary orgasm variable. Missing data were handled using pairwise deletion rather than FIML, as the latter is not available with the DWLS estimator required for categorical mediators. In sum, the model specified direct paths from gender, ONS type, and their interaction to each satisfaction component and to the other mediators. All mediators were allowed to covary, and each had a direct path to regret.

The model demonstrated poor fit to the observed data: CFI = 0.75, TLI = − 0.29, RMSEA = 0.30 (90% CI [0.28, 0.32]), and SRMR = 0.15. While these indices fall well below conventional thresholds for acceptable model fit, it is important to note that this model was not intended to provide optimal overall fit but rather to exploratorily decompose the effects of specific satisfaction components on regret. The full model statistics are listed in Table S10.

Examining which specific aspects of sexual satisfaction mediate gender differences in regret, our analysis revealed that orgasm achievement was the strongest mediator (*b* = − 0.16, 95% CI [− 0.22, − 0.11]) in heterosexual ONS, accounting for approximately 49% of the total indirect effect, *b* = − 0.33, 95% CI [− 0.41, − 0.25]. Sexual pleasure emerged as the second strongest satisfaction component mediator (*b* = − 0.04, 95% CI [− 0.07, − 0.03]), accounting for roughly 13% of the indirect effect, while partner’s ability to satisfy accounted for approximately 9% of the indirect effect (*b* = − 0.03, 95% CI [− 0.06, − 0.003]). Partner competence did not significantly mediate gender differences in regret (*b* = − 0.01, 95% CI [− 0.02, 0.01]).

For same-sex ONS, orgasm achievement (*b* = − 0.11, 95% CI [− 0.21, − 0.04]) and intoxication (*b* = − 0.04, 95% CI [− 0.08, − 0.02]) were significant, though the total indirect effect was not significant, *b* = − 0.15, 95% CI [− 0.32, 0.00]. The significant overall index of moderated mediation (*b* = 0.18, 95% CI [0.01, 0.34]) confirmed that mediation pathways differ between heterosexual and same-sex ONS, but only the indirect effect of gender on regret via intoxication (ln) differed significantly between ONS types, *b* = − 0.03, 95% CI [− 0.06, − 0.01].

In this exploratory analysis, orgasm achievement was the most influential component explaining gender differences in regret after heterosexual ONS, while subjective sexual pleasure and perceived partner abilities also made independent contributions. This suggests that sexual satisfaction’s impact on regret is multidimensional, extending beyond the mere presence or absence of orgasm.

##### Sexual Practices

Finally, we also followed up on the types of practices engaged in, given the dominance of satisfaction as a predictor of regret. In heterosexual ONS, vaginal sex was reported for 93.3% of the cases, anal sex for 6.6%, giving oral sex for 52%, receiving oral sex for 55.4%, while other practices than those listed occurred in 3.3% of the ONS. A proportion analysis of gender in reporting these practices showed a significant difference only for receiving oral sex. Of women, 45.57% reported receiving oral sex, 54.43% did not. Of men, 72.99% reported receiving oral sex, 27.01% did not: χ^2^ = 66.75, *df* = 1, *p* < 0.001. Men are significantly more likely to report receiving oral sex compared to women.

All practices except vaginal sex significantly positively predicted sexual satisfaction, but anal and vaginal sex were moderated by gender. Having anal sex predicted higher satisfaction for women than not having anal sex, averaged across all other practices, *b* = − 0.68, *p* < 0.001. It did not predict satisfaction in men. Similarly, having vaginal sex predicted higher satisfaction for women than not having vaginal sex, averaged across all other practices, *b* = − 0.34, *p* = 0.026, but not for men. Notably, receiving oral sex was the only sexual practice significantly predicting regret, with lower regret reported for ONS that involved the practice: *b* = − 0.22, *p* < 0.001. It was not moderated by gender. When adding satisfaction to the regression model, it was the only significant predictor of regret, *b* = − 0.32, *p* < 0.001, suggesting that the practices contribute to regret via sexual satisfaction.

In same-sex ONS, vaginal sex was reported in 24% of the times, anal sex 34.7%, giving oral sex 84%, receiving oral sex 78.7%, while other practices than those listed occurred in 13.3% of the ONS. Probably due to the small sample size, none of these practices predicted satisfaction or regret in same-sex ONS. The only significant effect was that receiving oral sex was linked to lower regret in men: the interaction of receiving oral sex and gender was significant, *b* = − 0.54, *p* = 0.016. Men who received oral sex reported lower regret than did men who did not.

#### Exploring Further Moderators

##### Intoxication as a Moderator

Being under the influence of drugs can also be theorized as a contextual variable and thus as a moderator. We explored the linear intoxication term as a moderator, controlling for the squared intoxication term, keeping the mediators as in the parallel SEM. The model demonstrated adequate but not excellent fit: CFI = 0.83, TLI = 0.65, RMSEA = 0.09 (90% CI [0.08, 0.11]). The SRMR was slightly above the recommended cut-off at 0.09. Although this model confirmed the U-shaped relationship between intoxication and regret (*b* = 0.04, *p* = 0.004), it did not support intoxication as a significant moderator of the gender gap. None of the intoxication interaction terms with gender or ONS type were significant: Gender × Intoxication: *b* = − 0.01, *p* = 0.649; ONS Type × Intoxication: *b* = 0.01, *p* = 0.760; Gender × ONS Type × Intoxication: *b* = − 0.00, *p* = 0.906. The gender gap in regret thus appears to unfold similarly across different intoxication levels.

##### Being a Student as a Moderator

Much prior research has focused specifically on ONS-induced regret of college students. Indeed, the contexts in which a ONS occurs for students differed systematically from those of nonstudents (see Table S11). For students, ONS occurred more frequently after parties than for non-students, whereas for nonstudents, ONS occurred more frequently after sex dates and after chance encounters than for students. We thus tested being a student as a moderator of gender and ONS type predicting regret. Yet, although dates and sex dates resulted in lower regret than parties (e.g., Table S1), being a student neither predicted regret nor interacted with gender, ONS type, nor both in predicting regret (*p*s > 0.562). This model had extremely poor fit: CFI = 0.22, TLI = 0.71, RMSEA = 0.40, SRMR = 0.09, and is not reported further.

##### Nationality as a Moderator

We explored whether nationality moderated the effects of gender and ONS type in regret (see Figure S18 for the distribution of regret by nationality). In a linear regression model, we predicted regret with gender, ONS type, nationality (reference: German), and their interactions: *R*^2^ = 0.07, *F*(19, 1022) = 4.87, *p* < 0.001. None of the estimates involving nationality were significant (*b*s < − 0.14, *p*s > 0.189). Gender and ONS type still significantly predicted regret.

## Discussion

Consistent with prior research, we found that sexual satisfaction, decision heteronomy, moral concern, and reputational concern strongly correlate with regret, while alcohol intoxication and emotional stability—uniquely among Big Five traits—also significantly predict regret. We replicate findings that ONS experiences are generally evaluated neutrally to positively, despite women reporting more negative assessments than men. The experience of regret is not uniformly distributed across genders, but varies systematically according to the gender composition of the ONS. Specifically, the distributional analyses revealed that the gender difference in post-ONS regret is context-dependent rather than universal. While heterosexual encounters showed meaningful gender differences with substantial non-overlap between distributions, same-sex encounters demonstrated near-complete distributional overlap and chance-level differences. This pattern suggests that the elevated regret commonly observed among women following CSREs may be driven primarily by dynamics specific to mixed-sex encounters rather than representing an inherent sex difference in psychological responses to casual sex.

The gender difference in regret after heterosexual ONS was fully explained by the explored mediating variables, namely sexual satisfaction, heteronomy, intoxication, and reputational concern. While similar correlations of the mediators with regret emerged for both heterosexual and same-sex ONS (Table S6), the higher likelihood to experience lower levels of satisfaction and higher levels of heteronomy, intoxication, and reputational concern accounted for the fact that women in heterosexual contexts reported more regret than all other gender and ONS combinations. Sexual satisfaction emerged as the strongest mediator, with orgasm achievement playing the critical role. Additionally, intoxication affected regret indirectly by decreasing sexual satisfaction and decision autonomy, but the serial mediation effects were small compared to the direct mediation of gender differences in regret via satisfaction, indicating that gender differences in sexual experiences remain robust even when controlling for substance use.

The quadratic relationship between intoxication and regret observed in our data offers a parsimonious explanation for previously contradictory findings regarding alcohol’s impact on post-casual sex evaluations. While some researchers reported minimal effects of alcohol consumption on emotional outcomes (Owen et al., 2010; Wesche et al., 2021b), others documented substantial negative consequences (Black et al., 2019; Lewis et al., 2012). Our analysis, which demonstrated superior fit for a quadratic function, suggests these disparate results may stem from methodological differences in capturing varying points along the intoxication spectrum. Studies using binary classification of intoxication or examining predominantly low-to-moderate consumption levels would detect minimal effects, whereas those sampling higher intoxication levels would observe stronger associations with negative outcomes. Furthermore, the accelerating nature of the relationship indicates a potential threshold effect wherein physiological impairment of sexual functioning and decision-making becomes pronounced only at higher consumption levels. Whether an explanation for prior findings or not, we clearly observed intoxication as a correlate of regret, of both heterosexual and same-sex ONS.

Contextual factors also influenced regret, with ONS following sex dates associated with less regret than those following parties, and more experienced individuals reporting lower regret overall. But the contribution of contextual factors to how positively the ONS was evaluated was small overall. Intercorrelations of contextual with experiential factors showed merely that sex dates are notably related to lower alcohol use. Age at the time of the ONS emerged as the most marked demographic-contextual correlate, associated with lower alcohol use, more autonomy, higher satisfaction, lower pregnancy and reputational concerns—suggesting that age positively predicts enjoyment of CSREs via these quality markers. This finding is consistent with the results of a recent diary study spanning seven semesters, where over time, satisfaction and intimacy with casual sex partners increased (Wesche et al., 2021b).

### Theoretical Implications

Our findings present challenges to strictly evolutionary explanations of gender differences in post-casual sex regret. The absence of a gender difference in same-sex ONS somewhat contradicts predictions derived from parental investment theory, which would anticipate consistent gender-based regret patterns regardless of partner gender. Similarly, the strong mediational role of satisfaction—particularly orgasm achievement—suggests experiential rather than evolved risk-assessment mechanisms contribute to post-ONS regret. Regarding reputational concern, while this variable significantly predicted regret, its mediational contribution was modest compared to satisfaction variables, suggesting it functions as a secondary rather than primary mechanism as evolutionary psychology might predict.

At the same time, the results are broadly consistent with sociocultural explanations, though they did not conclusively test them. Orgasm achievement revealed a large gender gap and constituted the strongest mediational pathway, which is in line with feminist critiques that heteronormative sexual scripts systematically prioritize male pleasure (Backstrom et al., 2012). For the other mediators, the connection to sociocultural factors is more speculative. Decision heteronomy emerged as a significant mediator, potentially indicating reduced perceived agency for women in heterosexual encounters. However, we cannot determine whether this reflects gendered power imbalances or alternative explanations such as differential decision-making styles.

While our findings appear more consistent with sociocultural explanations, it is important to note that evolutionary and sociocultural perspectives need not be mutually exclusive. Evolutionary predispositions may establish baseline tendencies that are then shaped, magnified, or minimized through sociocultural processes. The interaction between these forces likely creates the complex patterns observed in our data. Longitudinal designs would be necessary to more definitively test the causal mechanisms underlying these gender differences.

### Practical Implications

The predominant finding that most participants evaluated their ONS neutrally to positively is strongly in line with prior findings (Wesche et al., 2021a). The statistically significant gender difference in regret was moderate in size, and the proportion analyses revealed that about 70% of women’s most recent heterosexual ONS experiences resulted in higher regret than men’s average experiences. Critically, this reflects encounter-specific outcomes rather than stable individual characteristics. Given the within-person variation documented in diary studies (Wesche et al., 2021b), this supports focusing interventions on experiential factors rather than identifying “regret-prone” individuals.

The unidirectional nature of the effect suggests that systematic factors within heterosexual encounters drive these outcomes. This is also supported by the fact that controlling for emotional stability—a correlate of being male and experiencing less regret—did not account for gender differences in regret. It strengthens the case for addressing structural aspects of heterosexual interactions, particularly those identified in the mediation analysis (satisfaction and autonomy differences). Which type of sexual practices may promote the positivity of the experience differs for each individual (Backstrom et al., 2012), which was also reflected in our analyses of sexual practices predicting satisfaction and regret by gender. Enhancing the quality of individual CSREs through improved satisfaction, communication, and decision-making becomes the primary pathway for enhancing the neutral to positive outcomes further.

### Limitations and Future Research

The power analysis indicated that while the study had adequate power to detect the gender and the ONS type effect, it was underpowered for detecting the interaction between gender and ONS type. This limitation likely stems from the small and unbalanced sample sizes in the same-sex conditions, suggesting that the marginal significance observed for the interaction term should be interpreted with caution. The results underscore the need for larger samples of same-sex encounters. Those may reveal gender differences even in same-sex encounters, which may have gone undetected in our study.

A major methodological limitation concerns the cross-sectional, retrospective nature of our data, which raises concerns about the appropriateness of mediational analysis. Yet, although gender, our independent variable, is not manipulated, it can reasonably be treated as an exogenous variable. It temporally precedes the ONS experience and is not caused by any of our other variables. This similarly applies to the main moderator of our models: ONS type. Furthermore, asking participants specifically about their most recent ONS partially mitigates some limitations of the cross-sectional design while leaving others intact. It reduces selection bias by preventing participants from selectively reporting their most memorable (extremely positive or negative) experiences, which would likely overrepresent regretful encounters. By anchoring responses to the most recent event rather than any remembered ONS, we decrease the impact of memory selectivity bias and potentially capture more typical experiences across the sample. Additionally, focusing on the most recent ONS potentially reduced some memory degradation effects, as for many participants, the ONS occurred relatively recently.

However, fundamental limitations of the cross-sectional design remain. A single ONS, even the most recent one, may not represent an individual’s typical casual sex experiences. Moreover, without establishing temporal precedence between experiential factors and regret, causal inferences remain tentative. The retrospective nature of reporting allows for potential reconstruction bias, whereby participants who regretted their ONS may have retroactively distorted their evaluations to align with negative outcomes (cf. Kennair et al., 2018). While this concern cannot be fully dismissed without longitudinal data, several patterns in our results suggest that reported experiential factors are not merely post-hoc attributions. The quadratic relationship between intoxication and regret represents a nuanced pattern inconsistent with simple attribution effects. Systematic covariation between contextual variables (e.g., sex dates associated with both lower intoxication and lower regret) suggests genuine situational differences rather than overall distortion. The disproportionate mediating effect of orgasm achievement compared to other satisfaction components points to specific experiential mechanisms rather than general negative evaluations. Additionally, consistent age and experience effects across multiple variables suggest learning processes not readily explained by attribution biases. These patterns collectively indicate that while retrospective distortion likely contributes to our findings, the observed relationships also reflect substantive experiential differences.

There was a weak positive correlation between time elapsed and regret, which could be of interest when looking at the trajectories of regret. In our study, it could be explained by several mechanisms. First, memory biases may lead to reappraisal of casual sexual experiences over time, as immediate physical gratification becomes less salient while social norms and personal values gain prominence. That is, positive aspects of ONS experiences (physical pleasure, excitement) may fade more quickly from memory than negative aspects (moral concerns, decision-making issues), creating an increasingly negative overall evaluation. Second, as individuals mature and potentially enter committed relationships, their values regarding casual sex might shift to the negative.

Apart from memory effects, individuals who still experience regret after many years may have had a different experience than those whose regret diminishes over time. Their experience may have actually been more negative, thus causing lasting regret (Rozin & Royzman, 2001; Savitsky et al., 1997). The fact that the link between the mediating variables and regret strengthens over time somewhat supports that people reported on genuinely more negative experiences (Figure S3). The minor gender difference in this time effect suggests these memory and reappraisal processes operate slightly differently across genders, potentially reflecting differential psychological processes in how women and men integrate and reappraise casual sexual experiences over time (Figure S2). This could be explored in future research using longitudinal designs that track regret evaluations at multiple time points after casual sexual encounters, which would better distinguish between retrospective distortion and actual shifts in evaluation over time, and understanding which factors contribute most to lasting CSRE regret.

The level of emotional evaluations of ONS in this research is in line with levels of positivity reported in prior research, including those comparing CSREs with sexual experiences in committed relationships (Wesche et al., 2021a, 2021b). However, our recruitment specifically targeted people with ONS experience, and we excluded participants who never experienced an ONS. Among those who never experienced an ONS may be individuals who actively avoid casual sex due to anticipated regret. Indeed, 199 people indicated that they had the opportunity but did not make use of it. Our findings might thus underestimate the overall regret potential and misrepresent the population-wide gender gap. This is supported by the fact that women are less likely to engage in casual sex overall (e.g., Herold et al., 1998).

Although we extended this finding to 40 nationalities and showed that Germans, Austrians, US Americans, and Italians experienced casual sex in similar ways, cultural values were probably rather similar in our sample, exacerbated by selection bias introduced by sampling ONS-experienced individuals through the same, few channels (e.g., online discussion groups). In future research, cultural values surrounding ONS and other types of casual sex could be explored, as they are likely to influence the emotional consequences. For example, extramarital sex is legally prohibited in some regions of the world, specifically for women (Azam, 2013), typically in regions where the dominant religion is Islam. Casual sex should have substantially different emotional consequences in these regions. Indeed, religiosity predicts casual sex avoidance in college students, and has a slightly stronger impact on women’s sexual motivations than men’s (Hanna-Walker et al., 2023). Even within the US’ religious affiliation, the type of religion significantly predicts experience with casual sex, with Protestants reporting extramarital sex at a higher rate than all other religious groups and non-religious individuals (Davern et al., 2024). Thus, both broader societal values as well as subcultural and personal values are likely to interactively influence the retrospective evaluation of CSREs. Although we measured personality traits, we failed to assess personal sexual values, attitudes toward casual sex, or sexual self-schema, which could moderate the relationship between gender and regret independent of the measured mediators.

### Conclusion

This research advances our understanding of gender differences in casual sex regret by demonstrating that these differences appear to emerge largely from specific relational dynamics between women and men rather than from intrinsic gender characteristics. The non-significant gender difference in regret distributions for same-sex encounters, compared to the substantial difference in mixed-sex encounters, coupled with strong mediation through sexual satisfaction, suggests that structural aspects of heterosexual interactions constitute the primary mechanism underlying the gender gap in casual sex regret. Addressing these experiential factors presents clear opportunities for intervention, as women showed systematically higher regret than men across the entire distribution of heterosexual ONS experiences. Moving beyond simplistic narratives about gender differences, our results indicate that enhancing the quality of individual casual sexual encounters through improved communication, increased focus on mutual satisfaction, and promoting autonomous decision-making offers the most promising path toward reducing negative outcomes.

## Supplementary Information

Below is the link to the electronic supplementary material.

Supplementary file1 (DOCX 12833 KB)

## Data Availability

The materials, data, and analysis code are available here https://osf.io/vbe9h/.

## Footnotes

1 As participants were able to leave questions unanswered, the sample sizes vary slightly depending on the dependent variable in the model.
