# Supplementary material for: The Gender Gap in One-Night Stand Regret: Evidence from Heterosexual and Same-Sex Encounters
Source: Arch Sex Behav. 2026 Feb 25;55(2):589–612. doi: 10.1007/s10508-025-03380-3 (PMC13048962; doi:10.1007/s10508-025-03380-3)
Supplement: Supplementary file 1 — Supplementary file1 (DOCX 12833 KB) [file 10508_2025_3380_MOESM1_ESM.docx]

**Supplementary Information**

**For**

**The Gender Gap in One-Night Stand Regret: Evidence from Heterosexual and Same-Sex Encounters**

**Contents**

[Invitation Texts 1](#_Toc212448807)

[Time passed since the event 2](#_Toc212448808)

[Distribution of the Big Five Personality Variables by Gender and ONS Type 8](#_Toc212448809)

[Contextual variables 11](#_Toc212448810)

[Relationship status by gender and ONS type 13](#_Toc212448811)

[Intoxication: Analysis of the Relationship Between Subjective Intoxication and Regret 26](#_Toc212448812)

[Distribution of Sexual Experience Items 30](#_Toc212448813)

[Zero-order Correlations 32](#_Toc212448814)

[Heteroscedasticity due to uneven group size 39](#_Toc212448815)

[Reasons for Moral Concern 41](#_Toc212448816)

[Structural equation modelling – detailed path statistics 50](#_Toc212448817)

[ONS contexts for students and non-students 63](#_Toc212448818)

[Regret distribution for different nationalities 64](#_Toc212448819)

**List of Figures**

**Figure S1** Days passed since the ONS (log-transformed) by regret

**Figure S2** Days passed since the ONS (log-transformed) by regret and gender

**Figure S3** Relationship between satisfaction and regret by time passed since the ONS

**Figure S4** Personality trait distribution by gender and ONS type

**Figure S5** Relationship status at the time of the ONS by gender and ONS type

**Figure S6** Partner familiarity by gender and ONS type

**Figure S7** Occurrence context of the ONS by gender and ONS type

**Figure S8** Histograms of the experiential evaluation items and composite scores

**Figure S9** QQ Plots of the experiential evaluation items and composite scores

**Figure S10** Histograms plots of the sexual experience items and composite scores

**Figure S11** QQ Plots of the sexual experience items and composite scores

**Figure S12** Zero-order correlations of regret with the mediating variables and the Big Five

**Figure S13** Zero-order correlations of gender, ONS type, and regret with the experiential factors on item level

**Figure S14** Heteroscedasticity due to uneven group size

**Figure S15** Distribution of Moral Concerns by Gender and ONS Type

**Figure S16** Reasons for Moral Concerns by Gender and ONS Type

**Figure S17** Reasons for moral concern by ONS type and relationship status

**Figure S18** Regret distribution for different nationalities

**List of Tables**

**Table S1** Characteristics of the sample and the ONS for women and men by partner gender

**Table S2** Correlations of context variables with experiential factors of the ONS

**Table S3** Drug use and subjective intensity of widely consumed substances by participants and partner

**Table S4** Model Comparisons for the Relationship Between Subjective Intoxication and Regret

**Table S5 Zero-Order Correlation of the Main Predictors with the Experiential Factors and Personality Traits**

**Table S6** Zero-order correlations of the mediating variables with regret by ONS type

**Table S7** Reasons indicated for moral concern as an open text response

**Table S8** Parallel SEM including emotional stability as a covariate

**Table S9** Serial mediation SEM

**Table S10** Satisfaction components SEM

**Table S11** Frequency of different ONS contexts for students and non-students

# Invitation Texts

*Version 1: text used for university mailing list*

Dear students!

Do you have experience with one-night stands (single sexual encounters)? Then we would like to ask you to participate in an exciting current study from the Social Psychology work group, which will take approximately 5 minutes of your time. The aim of the study is to shed more light on the experience of one-night stands and the individual feelings and experiences involved; your information can make a significant contribution to this!

Access: Link to study

Your data is subject to data protection and will be used only for scientific research purposes. You can withdraw from the survey at any time without giving reasons and without personal disadvantage. We would like to thank you very much in advance for your participation and thus also for your support in this study. We are of course available to answer any questions you may have.

*Version 2: text used in social media and forum posts*

Walk of Shame or Walk of Fame?
Do you know “the morning after”? Have you ever had a one-night stand? Then we are interested in what you experienced and how you experienced it. The Social Psychology work group at the University of Innsbruck is currently researching the experience of one-night stands. Take the time to complete a short questionnaire (approx. 5 minutes) and tell us about your feelings and experiences.
Thank you very much!

# Time passed since the event

**Figure S1**

*Days passed since the ONS (log-transformed) by regret*


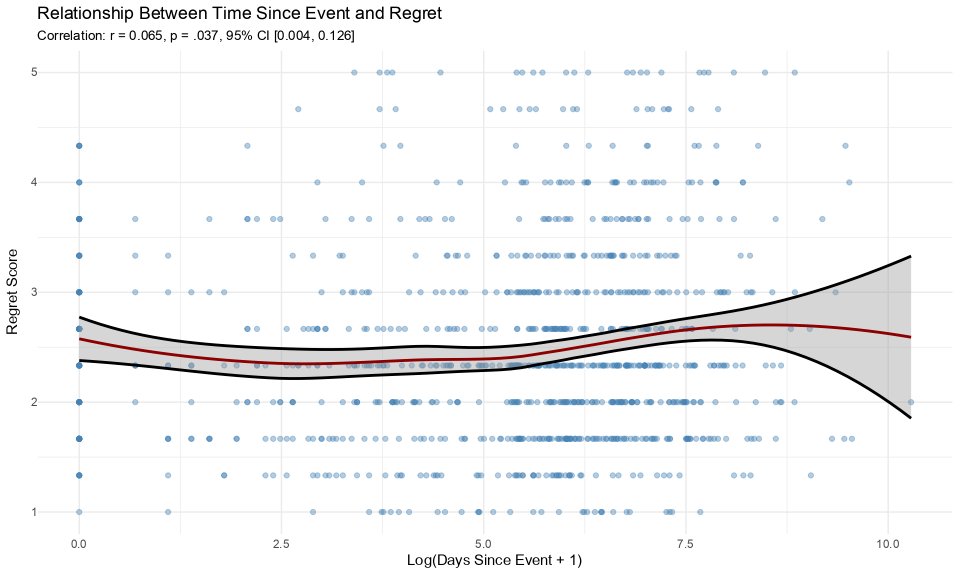


*Note.* Relationship between time elapsed since the one-night stand and retrospective regret. The scatter plot displays individual observations (n = 1,037) with log-transformed days since the event on the x-axis and regret scores on the y-axis. The dark red curve represents a locally estimated scatterplot smoothing (LOESS) fit with 95% confidence interval (shaded area). While the linear correlation is weak (*r* = .07, *p* = .037, 95% CI [0.004, 0.126]), the non-linear pattern suggests two possible interpretations: either regret gradually increases with temporal distance as individuals reappraise past casual encounters, or alternatively, experiences that were genuinely more negative produced more lasting regret and are thus overrepresented among distant memories. We discuss this interpretation further below and in the General Discussion of the main manuscript.

***Generalized Additive Model Analysis of Time-Regret Relationship***

To formally test whether the relationship between time since the ONS and reported regret follows a non-linear pattern, we conducted Generalized Additive Model (GAM) analyses separately for each gender. For women (n = 638), the GAM revealed a statistically significant non-linear relationship between log-transformed days since the event and regret scores (edf = 2.17, Ref.df = 2.71, *F* = 3.3, *p* = .025). An ANOVA comparison confirmed that this non-linear model fit the data significantly better than a simple linear regression (*F* = 6.10, *p* = .010). The smooth term’s effective degrees of freedom exceeding 1.0 mathematically validates the curved pattern observed in the LOESS visualization, though the overall effect size remains modest (*R*² adjusted = .013, deviance explained = 1.66%).

For men (n = 385), the GAM converged to an essentially linear solution (edf = 1.00) with the smooth term reaching only marginal significance (*F* = 3.18, *p* = .075), explaining less than 1% of variance in regret scores (*R*² adjusted = .006, deviance explained = 0.83%). These findings confirm distinct temporal patterns in post-ONS regret between genders: women’s regret follows a significant non-linear trajectory over time, while men’s regret shows a weaker, essentially linear relationship with time elapsed. This gender difference in temporal dynamics may reflect differential psychological processes in how women and men integrate and reappraise casual sexual experiences over time.

**Figure S2**

*Days passed since the ONS (log-transformed) by regret and gender*


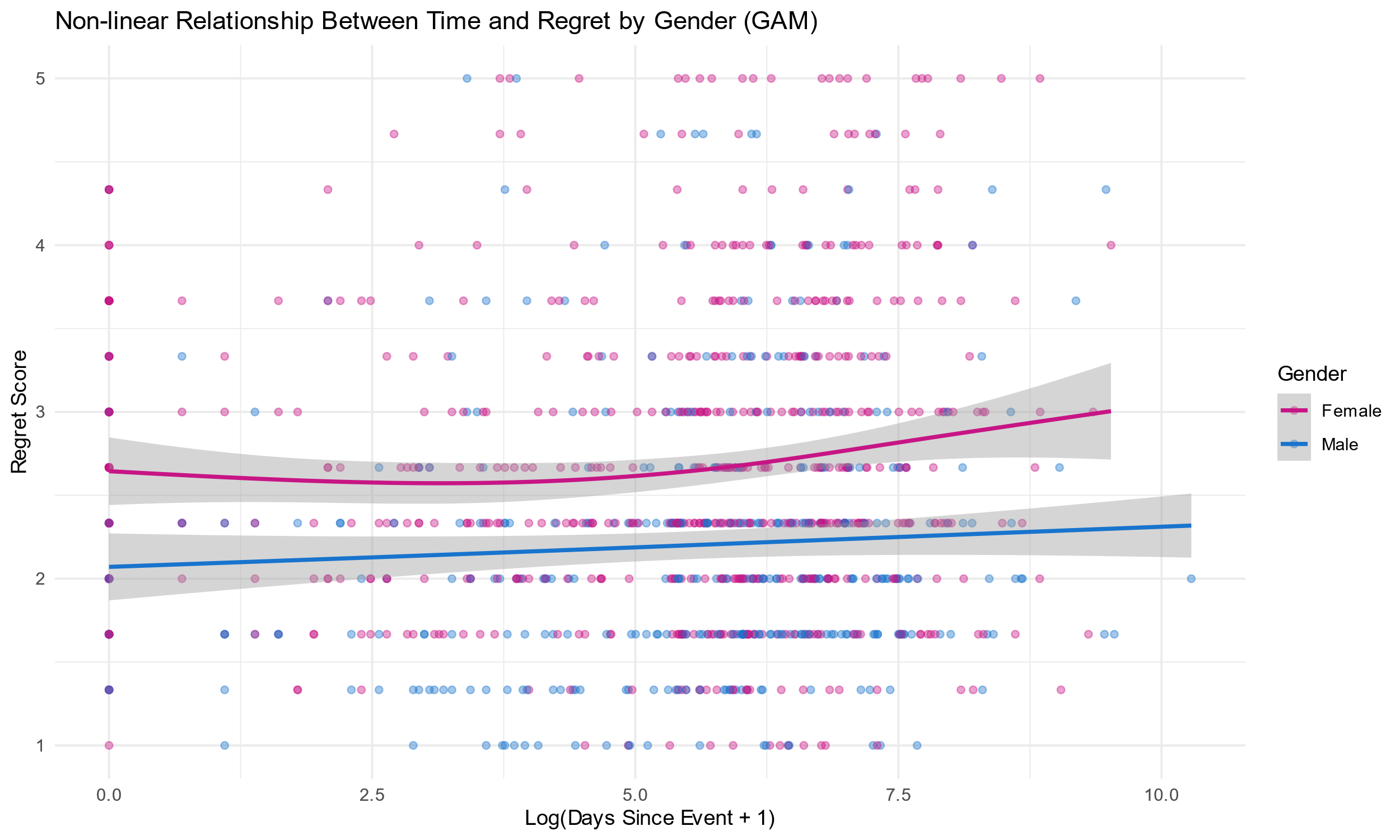


*Note.* The figure displays the relationship between log-transformed time since event and regret scores, with separate trend lines for women and men. Women’s regret follows a significant non-linear pattern, initially decreasing slightly before increasing over longer time periods. In contrast, men’s pattern remains relatively flat with a slight positive linear trend that was marginally significant. Points represent individual observations (alpha = 0.4 for visibility of density).

We created a 2-categorical variable for days since the ONS for recent (1 year or less) and distant (more than 1 year ago) ONS. We then examined whether it moderated the effect of gender and ONS type on regret in a linear regression model predicting regret with gender * ONS type * recency. While the pattern of the two main predictors remained unchanged, recency also predicted regret, with higher regret for those who reported on a more distant ONS, reflecting the pattern from the zero-order correlations: *b* = -0.15, *SE* = 0.07, *t* = -2.17, *p* = .030. Yet, it did not moderate any of the effects or interactions (*p*s > .266), suggesting that whether an ONS was recent or distant did not alter the pattern of gender differences in heterosexual vs. same-sex ONS.

We also repeated our basic regression model after excluding participants who had experienced their ONS more than 3 years ago, resulting in 822 participants. The pattern of results was again identical to the model including the whole sample: *R*^2^ = .07, *F*(3, 818) = 20.20, *p* < .001, with women in heterosexual ONS reporting higher levels of regret than all other groups (all *p* < .005, Tukey-adjusted), while these did not differ from each other (*p* > .729). For example, the gender difference in same-sex ONS based on estimated marginal means was *b* = -0.07, *p* = .989, while the gender difference in heterosexual ONS was *b* = 0.44, *p* < .001.

To understand the development of regret over time in more depth, we explored which mediators were predicted by time passed since the event. Only satisfaction was significantly predicted by time since the event, with the most recent group (ONS in the past month) experiencing significantly higher levels of satisfaction (*EMM* = 3.51, *SE* = 0.10) than all other groups (*b*s > 0.18, *p*s > .044), except the second most recent one (1-3 months, *EMM* = 3.40, *SE* = 0.11; *p* = .552). This pattern was replicated for each satisfaction component (orgasm achievement, pleasure, ability to satisfy and competence).

Time since event significantly moderated the effect of satisfaction, heteronomy, reputational concern, and physical disgust, respectively, on regret. The pattern for satisfaction is shown in this figure. The correlation was stronger when the ONS happened more than three years ago than when it happened less than one month ago, *b* = -0.24, *SE* = 0.09, *t* = -2.78, *p* = .005

**Figure S3**

*Relationship between satisfaction and regret by time passed since the ONS*


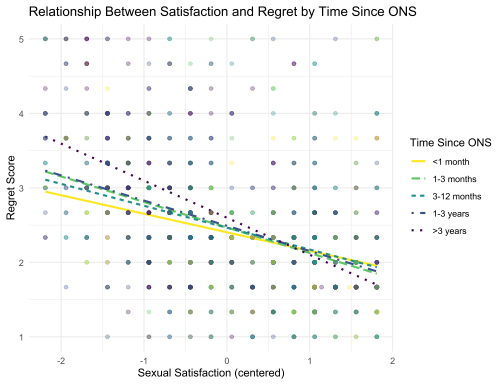


The other mediators showed similar trend as did satisfaction. For ONS that happened more than three years ago, decision heteronomy, reputational concern, and physical disgust were significantly more strongly correlated with regret than for ONS that happened less than one month ago, while simple slope analyses showed that the correlations were significant in the same direction for all mediators in all time categories.

These findings can be interpreted in two main ways. Negativity bias (e.g., Rozin & Royzman, 2001) predicts that genuinely negative experiences (characterized by low satisfaction, compromised autonomy, and elevated disgust) persist in generating regret over extended periods, while more benign experiences see diminished regret over time. This would produce the observed strengthening correlations as the sample gradually becomes weighted toward more genuinely negative encounters in the distant memory categories.

Alternatively, a memory reconstruction account proposes that for experiences that continue generating regret over years, individuals may retrospectively amplify negative aspects through repeated retrieval and reappraisal processes. Under this interpretation, the strengthened correlations represent increasing memory coherence rather than selective retention of truly negative experiences.

Additional explanations include cohort effects (older ONS occurring under different normative social conditions) or developmental processes (evolving values and life contexts altering interpretations). The cross-sectional nature of our data does not allow us to discriminate between these competing interpretations, as we cannot track how individual memories evolve longitudinally. Nevertheless, these findings provide empirical support for the proposition that regret after longer periods represents more than simple memory persistence and reflects qualitatively different relationships with its experiential determinants.

# Distribution of the Big Five Personality Variables by Gender and ONS Type

To test whether Big Five personality traits differ by gender (female, male) and ONS type (heterosexual, same-sex), we conducted a multivariate analysis of variance (MANOVA) with the five personality traits (extraversion, agreeableness, conscientiousness, emotional stability, openness) as dependent variables. Follow-up univariate ANOVAs were subsequently performed for each personality trait to identify specific differences. The distribution of the variables is visualized in Figure S4.

Prior to analysis, we tested MANOVA assumptions. Shapiro-Wilk tests indicated violations of multivariate normality across most groups and traits (*p* < .05). Box’s M test for homogeneity of covariance matrices was significant (χ² = 66.59, *df* = 45, *p* = .020), indicating this assumption was also violated. Given these violations and the unbalanced design, we used Pillai’s trace as the test statistic and Type III sum of squares for follow-up analyses, both of which are more robust to assumption violations (Zwick, 1985^[[1]](#footnote-1)^). The MANOVA revealed a significant main effect of gender, Pillai’s trace = 0.103, *F*(5, 1033) = 23.73, *p* < .001, and ONS type, Pillai’s trace = 0.013, *F*(5, 1033) = 2.69, *p* = .020 on the combined personality traits. The interaction between gender and ONS type approached significance, Pillai’s trace = 0.009, *F*(5, 1033) = 1.88, *p* = .095.

For extraversion, analysis revealed a significant but small main effect of ONS type, *F*(1, 1037) = 5.55, *p* = .019, partial η² = .005, with individuals reporting heterosexual ONS scoring higher on extraversion (women: *M* = 4.64, *SD* = 1.48; men: *M* = 4.43, *SD* = 1.49) than those reporting same-sex ONS (women: *M* = 4.07, SD = 1.74; men: *M* = 4.13, SD = 1.73). No significant effect of gender (*F*(1, 1037) = 0.17, *p* = .684) or the interaction (*F*(1, 1037) = 0.50, *p* = .478) was observed.

For agreeableness, results indicated a significant but small main effect of gender, *F*(1, 1037) = 10.06, *p* = .002, partial η² = .005, with women scoring higher on agreeableness (hetero: *M* = 5.39, *SD* = 1.13; same-sex: *M* = 5.57, *SD* = 0.87) than men (hetero: *M* = 5.13, *SD* = 1.14; same-sex: *M* = 4.94, *SD* = 1.32). Neither ONS type (*F*(1, 1037) = 0.002, *p* = .965) nor the interaction (*F*(1, 1037) = 1.76, *p* = .185) reached significance.

For conscientiousness, the analysis showed no significant main effect of gender (*F*(1, 1037) = 0.40, p = .526). ONS type approached significance (*F*(1, 1037) = 2.82, *p* = .094), as did the interaction (*F*(1, 1037) = 3.42, *p* = .065). The interaction trend suggests that women with heterosexual ONS reported higher conscientiousness (*M* = 5.20, *SD* = 1.31) than women with same-sex ONS (*M* = 4.61, *SD* = 1.37), while men showed similar conscientiousness levels regardless of ONS type (hetero: *M* = 4.99, *SD* = 1.40; same-sex: *M* = 5.02, *SD* = 1.37). Both effects are small in size.

For, emotional stability, the results revealed significant but small main effects of both gender (*F*(1, 1037) = 8.47, *p* = .004, partial η² = .008) and ONS type (*F*(1, 1037) = 4.90, *p* = .027, partial η² = .005). Men reported higher emotional stability than women, and individuals reporting heterosexual ONS showed higher emotional stability than those reporting same-sex ONS. No significant interaction was observed (*F*(1, 1037) = 0.90, *p* = .343).

Finally, for openness to experience, the results indicated a significant main effect of gender (*F*(1, 1037) = 4.39, *p* = .036), with women scoring higher on openness than men. No significant effect of ONS type (*F*(1, 1037) = 1.08, *p* = .299) or interaction (*F*(1, 1037) = 0.18, *p* = .667) was detected.

In summary, there are significant gender differences in agreeableness (women higher), emotional stability (men higher), and openness (women higher). ONS type was associated with differences in extraversion and emotional stability, with heterosexual ONS participants scoring higher on both traits. Conscientiousness showed a marginally significant interaction pattern, suggesting that the effect of gender may depend on ONS type. While statistically significant, all univariate effects were small, indicating that gender and ONS type account for a modest proportion of variance in personality traits. The multivariate effect of gender was more substantial (Pillai’s trace = 0.103), suggesting that the combined personality profile differs more meaningfully between men and women than individual traits considered separately.

**Figure S4**

*Personality trait distribution by gender and ONS type*


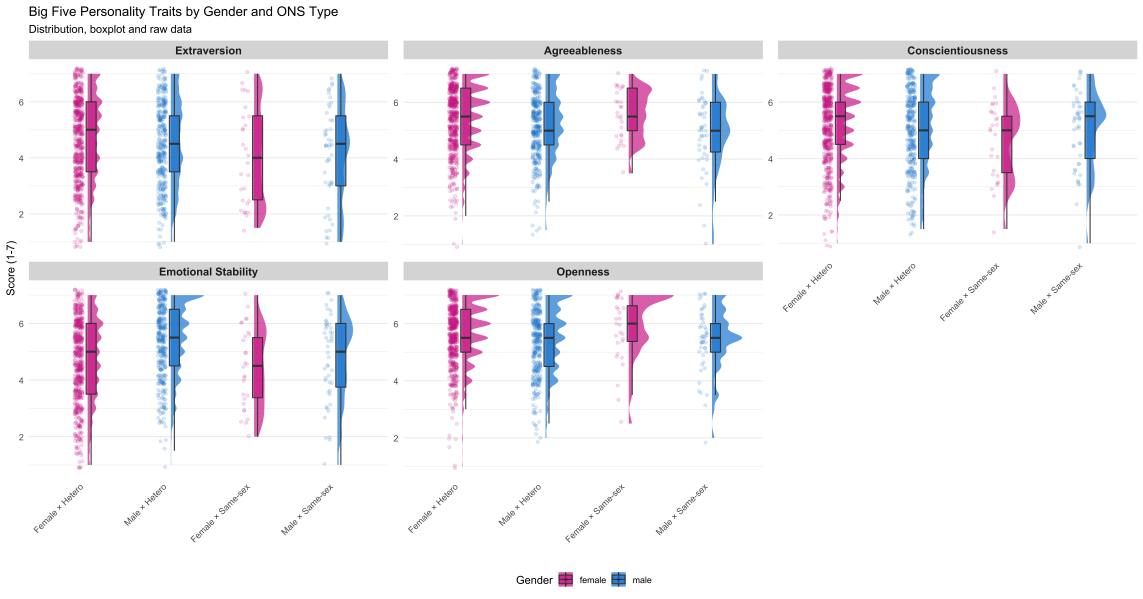


# Contextual variables

**Table S1**

*Characteristics of the sample and the ONS for women and men by partner gender*

| Participant Gender | Women | | | | Men | | | |
| --- | --- | --- | --- | --- | --- | --- | --- | --- |
| Partner Gender | Female | | Male | | Female | | Male | |
|  | Mean | SD | Mean | SD | Mean | SD | Mean | SD |
| Age | 26.71 | 5.28 | 24.3 | 5.90 | 26.18 | 6.87 | 26.64 | 8.13 |
| Number of prior ONS | 10 | 15 | 6 | 12 | 7 | 12 | 32 | 83 |
|  | n | % | n | % | n | % | n | % |
| Relationship status at the time of the ONS |  |  |  |  |  |  |  |  |
| Single | 20 | 71.4 | 492 | 79.5 | 267 | 76.7 | 35 | 74.5 |
| In a relationship | 6 | 21.4 | 34 | 5.5 | 42 | 12.1 | 7 | 14.9 |
| Just broken up | 2 | 7.1 | 83 | 13.4 | 36 | 10.3 | 4 | 8.5 |
| Almost broken up | 0 | 0 | 10 | 1.6 | 3 | 0.9 | 1 | 2.1 |
| Practices |  |  |  |  |  |  |  |  |
| Given oral sex | 22 | 78.6 | 317 | 51 | 187 | 53.7 | 41 | 87.2 |
| Received oral sex | 21 | 75 | 283 | 45.6 | 254 | 73 | 38 | 80.9 |
| Vaginal sex | 14 | 50 | 576 | 92.8 | 328 | 94.3 | 4 | 8.5 |
| Anal sex | 0 | 0 | 37 | 6 | 27 | 7.8 | 26 | 55.3 |
| Context |  |  |  |  |  |  |  |  |
| After a date | 1 | 3.6 | 90 | 14.5 | 65 | 18.7 | 4 | 8.5 |
| After a party | 10 | 35.7 | 320 | 51.5 | 149 | 42.8 | 8 | 17 |
| After a small social gathering | 4 | 14.3 | 87 | 14 | 50 | 14.4 | 3 | 6.4 |
| After a sex date | 7 | 25 | 80 | 12.9 | 49 | 14.1 | 23 | 48.9 |
| After an unplanned encounter | 5 | 17.9 | 42 | 6.8 | 28 | 8 | 8 | 17 |
| other | 1 | 3.6 | 2 | 0.3 | 7 | 2 | 1 | 2.1 |
| Partner known |  |  |  |  |  |  |  |  |
| Met for the first time on same day | 17 | 60.7 | 334 | 53.8 | 185 | 53.2 | 36 | 76.6 |
| Met recently (minimally known) | 0 | 0 | 16 | 2.6 | 7 | 2 | 0 | 0 |
| Dating partner | 3 | 10.7 | 54 | 8.7 | 38 | 10.9 | 0 | 0 |
| Known for a while (e.g., acquaintance) | 5 | 17.9 | 171 | 27.5 | 79 | 22.7 | 9 | 19.1 |
| Good friend | 1 | 3.6 | 22 | 3.5 | 21 | 6 | 1 | 2.1 |
| Ex-partner, friend's partner or ex-partner | 1 | 3.6 | 10 | 1.6 | 8 | 2.3 | 1 | 2.1 |
| Work colleague | 0 | 0 | 4 | 0.6 | 3 | 0.9 | 0 | 0 |
| Other | 1 | 3.6 | 10 | 1.6 | 7 | 2 | 0 | 0 |
| Orgasm |  |  |  |  |  |  |  |  |
| Yes | 13 | 46 | 147 | 24 | 273 | 78 | 39 | 83 |
| No | 15 | 54 | 473 | 76 | 75 | 22 | 8 | 17 |

# Relationship status by gender and ONS type

To examine whether the proportions of relationship statuses differ significantly between groups, we conducted pairwise chi-square tests for each combination of relationship status and group. The p-values were corrected for multiple testing using Holm’s method. The analysis revealed no significant differences in the proportions of relationship statuses between the groups after correcting for multiple testing. All corrected p-values were above the threshold for significance.

**Figure S5**

*Relationship status at the time of the ONS by gender and ONS type*


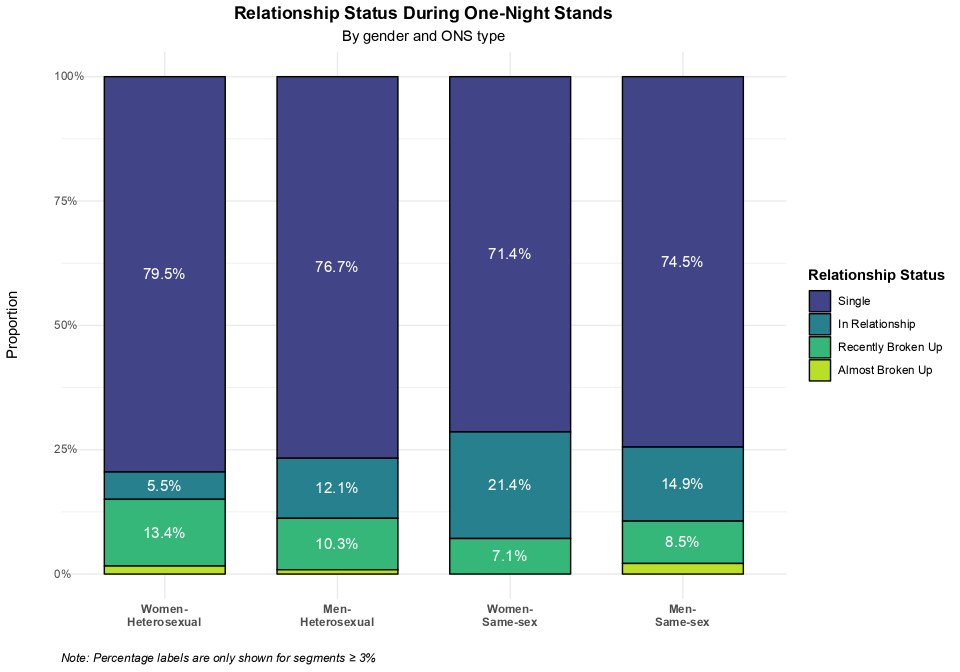


**Figure S6**

*Partner familiarity by gender and ONS type*


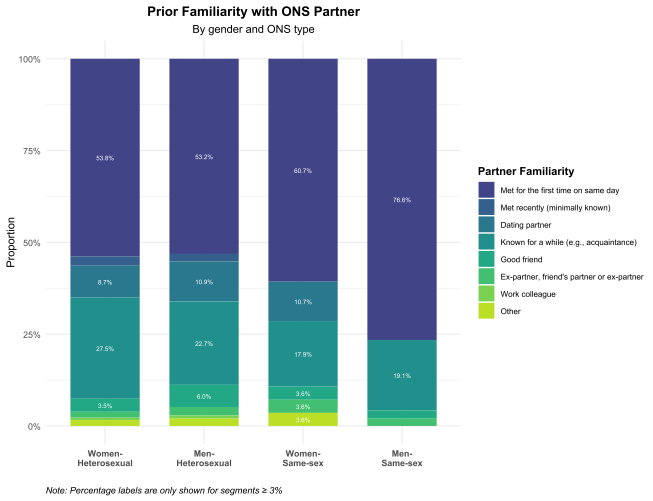


**Figure S7**

*Occurrence context of the ONS by gender and ONS type*


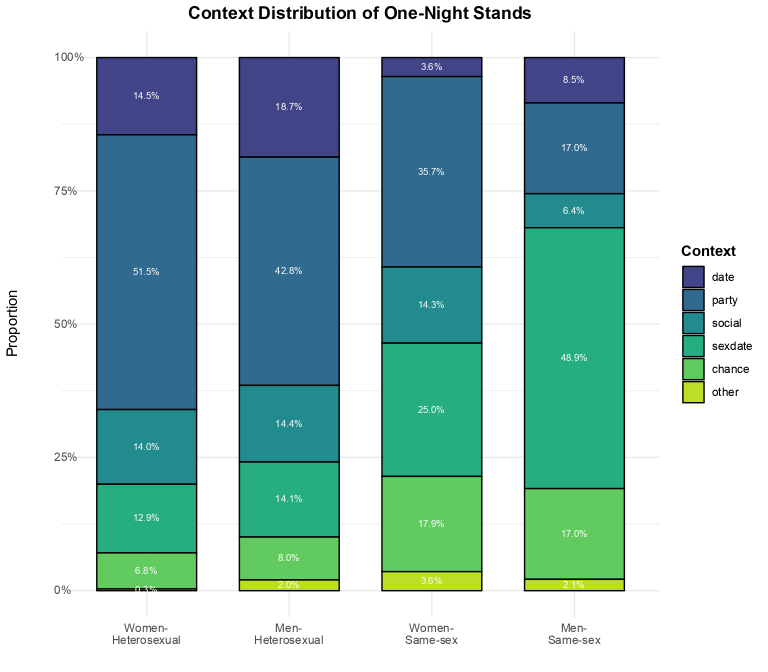


**Table S2**

*Correlations of context variables with experiential factors of the ONS*

| **Variable 1** | **Variable 2** | **Correlation** | **LL CI** | **UL CI** | ***p*** | **Method** | **Holm-adjusted *p*** | **Significance** |
| --- | --- | --- | --- | --- | --- | --- | --- | --- |
| Sex date | Intoxication (ln) | -0.36 | -0.41 | -0.31 | 0.000 | Point-biserial | 0.000 | *** |
| Age at ONS | ONS count (log) | 0.31 | 0.25 | 0.37 | 0.000 | Pearson | 0.000 | *** |
| Date | Partner familiarity | 0.27 | 0.20 | 0.34 | 0.000 | Cramer's V | 0.000 | *** |
| Age at ONS | Intoxication (ln) | -0.25 | -0.30 | -0.19 | 0.000 | Pearson | 0.000 | *** |
| In a relationship | Age at ONS | 0.23 | 0.17 | 0.29 | 0.000 | Point-biserial | 0.000 | *** |
| In a relationship | Moral concern | 0.22 | 0.16 | 0.27 | 0.000 | Point-biserial | 0.000 | *** |
| Age at ONS | Heteronomy | -0.21 | -0.27 | -0.15 | 0.000 | Pearson | 0.000 | *** |
| Age at ONS | Sexual satisfaction | 0.20 | 0.14 | 0.26 | 0.000 | Pearson | 0.000 | *** |
| Date | Intoxication (ln) | -0.20 | -0.26 | -0.14 | 0.000 | Point-biserial | 0.000 | *** |
| ONS count (log) | Sexual satisfaction | 0.20 | 0.14 | 0.25 | 0.000 | Pearson | 0.000 | *** |
| ONS count (log) | Heteronomy | -0.17 | -0.23 | -0.12 | 0.000 | Pearson | 0.000 | *** |
| Social gathering | Partner familiarity | 0.18 | 0.12 | 0.25 | 0.000 | Cramer's V | 0.000 | *** |
| Age at ONS | Days since the event (log) | -0.16 | -0.22 | -0.10 | 0.000 | Pearson | 0.000 | *** |
| ONS count (log) | Regret | -0.16 | -0.22 | -0.10 | 0.000 | Pearson | 0.000 | *** |
| Sex date | Intoxication (squ) | 0.16 | 0.10 | 0.22 | 0.000 | Point-biserial | 0.000 | *** |
| Partner familiarity | Reputational concern | 0.03 | 0.00 | 0.01 | 0.000 | Eta-squared | 0.000 | *** |
| Sex date | ONS count (log) | 0.15 | 0.09 | 0.21 | 0.000 | Point-biserial | 0.000 | *** |
| Age at ONS | Regret | -0.14 | -0.20 | -0.08 | 0.000 | Pearson | 0.001 | ** |
| In a relationship | Sexual satisfaction | 0.14 | 0.08 | 0.20 | 0.000 | Point-biserial | 0.002 | ** |
| Partner familiarity | Intoxication (ln) | 0.02 | 0.00 | 0.01 | 0.000 | Eta-squared | 0.003 | ** |
| Sex date | Heteronomy | -0.13 | -0.19 | -0.07 | 0.000 | Point-biserial | 0.003 | ** |
| Date | Age at ONS | 0.13 | 0.07 | 0.19 | 0.000 | Point-biserial | 0.003 | ** |
| Met by chance | ONS count (log) | 0.13 | 0.07 | 0.19 | 0.000 | Point-biserial | 0.004 | ** |
| Partner familiarity | STI concern | 0.02 | 0.00 | 0.01 | 0.000 | Eta-squared | 0.004 | ** |
| Age at ONS | Partner familiarity | 0.02 | 0.00 | 0.01 | 0.000 | Eta-squared | 0.010 | * |
| Sex date | Age at ONS | 0.12 | 0.06 | 0.18 | 0.000 | Point-biserial | 0.012 | * |
| Age at ONS | Reputational concern | -0.12 | -0.18 | -0.06 | 0.000 | Pearson | 0.019 | * |
| ONS count (log) | Moral concern | -0.12 | -0.18 | -0.06 | 0.000 | Pearson | 0.020 | * |
| ONS count (log) | Intoxication (ln) | -0.12 | -0.18 | -0.06 | 0.000 | Pearson | 0.021 | * |
| Age at ONS | Pregnancy concern | -0.12 | -0.18 | -0.06 | 0.000 | Pearson | 0.023 | * |
| ONS count (log) | Pregnancy concern | -0.12 | -0.18 | -0.06 | 0.000 | Pearson | 0.028 | * |
| In a relationship | Intoxication (squ) | 0.11 | 0.05 | 0.17 | 0.000 | Point-biserial | 0.038 | * |
| In a relationship | ONS count (log) | 0.11 | 0.05 | 0.17 | 0.000 | Point-biserial | 0.038 | * |
| ONS count (log) | Days since the event (log) | -0.11 | -0.17 | -0.05 | 0.000 | Pearson | 0.047 | * |
| Sex date | Regret | -0.11 | -0.17 | -0.05 | 0.000 | Point-biserial | 0.061 |  |
| Age at ONS | Moral concern | -0.11 | -0.17 | -0.05 | 0.000 | Pearson | 0.072 |  |
| Met by chance | Age at ONS | 0.11 | 0.05 | 0.17 | 0.001 | Point-biserial | 0.082 |  |
| Partner familiarity | Moral concern | 0.01 | 0.00 | 0.01 | 0.001 | Eta-squared | 0.110 |  |
| ONS count (log) | Partner familiarity | 0.01 | 0.00 | 0.01 | 0.001 | Eta-squared | 0.138 |  |
| Age at ONS | Physical disgust | -0.10 | -0.16 | -0.04 | 0.001 | Pearson | 0.144 |  |
| Partner familiarity | Physical disgust | 0.01 | 0.00 | 0.01 | 0.001 | Eta-squared | 0.157 |  |
| In a relationship | Reputational concern | 0.10 | 0.04 | 0.16 | 0.001 | Point-biserial | 0.159 |  |
| Sex date | Sexual satisfaction | 0.10 | 0.04 | 0.16 | 0.001 | Point-biserial | 0.166 |  |
| Other context | Intoxication (ln) | -0.10 | -0.16 | -0.04 | 0.002 | Point-biserial | 0.213 |  |
| In a relationship | Pregnancy concern | -0.10 | -0.16 | -0.04 | 0.002 | Point-biserial | 0.270 |  |
| In a relationship | Intoxication (ln) | -0.09 | -0.15 | -0.02 | 0.006 | Point-biserial | 0.799 |  |
| Date | Reputational concern | -0.08 | -0.14 | -0.02 | 0.007 | Point-biserial | 0.906 |  |
| Almost broken up | Age at ONS | 0.04 | -0.02 | 0.10 | 0.235 | Point-biserial | 1 |  |
| Other context | Age at ONS | 0.02 | -0.04 | 0.08 | 0.486 | Point-biserial | 1 |  |
| Recently broken up | Age at ONS | -0.05 | -0.11 | 0.01 | 0.128 | Point-biserial | 1 |  |
| Social gathering | Age at ONS | -0.01 | -0.08 | 0.05 | 0.643 | Point-biserial | 1 |  |
| Almost broken up | Days since the event (log) | -0.04 | -0.10 | 0.02 | 0.207 | Point-biserial | 1 |  |
| Date | Days since the event (log) | -0.02 | -0.08 | 0.04 | 0.599 | Point-biserial | 1 |  |
| In a relationship | Days since the event (log) | -0.08 | -0.14 | -0.02 | 0.009 | Point-biserial | 1 |  |
| Met by chance | Days since the event (log) | -0.01 | -0.07 | 0.05 | 0.687 | Point-biserial | 1 |  |
| Other context | Days since the event (log) | 0.03 | -0.03 | 0.10 | 0.267 | Point-biserial | 1 |  |
| Partner familiarity | Days since the event (log) | 0.00 | 0.00 | 0.01 | 0.125 | Eta-squared | 1 |  |
| Recently broken up | Days since the event (log) | 0.04 | -0.02 | 0.10 | 0.245 | Point-biserial | 1 |  |
| Sex date | Days since the event (log) | -0.07 | -0.13 | -0.01 | 0.017 | Point-biserial | 1 |  |
| Social gathering | Days since the event (log) | 0.02 | -0.05 | 0.08 | 0.608 | Point-biserial | 1 |  |
| Almost broken up | Heteronomy | 0.02 | -0.04 | 0.09 | 0.425 | Point-biserial | 1 |  |
| Date | Heteronomy | -0.01 | -0.07 | 0.05 | 0.777 | Point-biserial | 1 |  |
| In a relationship | Heteronomy | -0.06 | -0.12 | 0.00 | 0.048 | Point-biserial | 1 |  |
| Met by chance | Heteronomy | -0.03 | -0.09 | 0.03 | 0.402 | Point-biserial | 1 |  |
| Other context | Heteronomy | -0.02 | -0.08 | 0.05 | 0.611 | Point-biserial | 1 |  |
| Partner familiarity | Heteronomy | 0.00 | 0.00 | 0.01 | 0.553 | Eta-squared | 1 |  |
| Recently broken up | Heteronomy | 0.00 | -0.06 | 0.06 | 0.945 | Point-biserial | 1 |  |
| Social gathering | Heteronomy | 0.03 | -0.03 | 0.09 | 0.382 | Point-biserial | 1 |  |
| Almost broken up | Intoxication (ln) | -0.02 | -0.08 | 0.04 | 0.540 | Point-biserial | 1 |  |
| Met by chance | Intoxication (ln) | -0.08 | -0.14 | -0.02 | 0.008 | Point-biserial | 1 |  |
| Recently broken up | Intoxication (ln) | 0.00 | -0.06 | 0.06 | 0.913 | Point-biserial | 1 |  |
| Social gathering | Intoxication (ln) | -0.01 | -0.07 | 0.05 | 0.694 | Point-biserial | 1 |  |
| Age at ONS | Intoxication (squ) | 0.07 | 0.01 | 0.13 | 0.024 | Pearson | 1 |  |
| Almost broken up | Intoxication (squ) | 0.01 | -0.05 | 0.07 | 0.686 | Point-biserial | 1 |  |
| Date | Intoxication (squ) | -0.01 | -0.07 | 0.05 | 0.699 | Point-biserial | 1 |  |
| Met by chance | Intoxication (squ) | 0.08 | 0.02 | 0.14 | 0.013 | Point-biserial | 1 |  |
| ONS count (log) | Intoxication (squ) | 0.05 | -0.01 | 0.11 | 0.108 | Pearson | 1 |  |
| Other context | Intoxication (squ) | 0.06 | 0.00 | 0.12 | 0.044 | Point-biserial | 1 |  |
| Partner familiarity | Intoxication (squ) | 0.00 | 0.00 | 0.01 | 0.633 | Eta-squared | 1 |  |
| Recently broken up | Intoxication (squ) | 0.01 | -0.05 | 0.07 | 0.701 | Point-biserial | 1 |  |
| Social gathering | Intoxication (squ) | -0.03 | -0.09 | 0.03 | 0.373 | Point-biserial | 1 |  |
| Almost broken up | Moral concern | 0.06 | 0.00 | 0.12 | 0.038 | Point-biserial | 1 |  |
| Date | Moral concern | -0.03 | -0.09 | 0.03 | 0.328 | Point-biserial | 1 |  |
| Met by chance | Moral concern | -0.02 | -0.08 | 0.04 | 0.453 | Point-biserial | 1 |  |
| Other context | Moral concern | -0.04 | -0.10 | 0.03 | 0.258 | Point-biserial | 1 |  |
| Recently broken up | Moral concern | 0.01 | -0.05 | 0.07 | 0.738 | Point-biserial | 1 |  |
| Sex date | Moral concern | -0.05 | -0.11 | 0.01 | 0.085 | Point-biserial | 1 |  |
| Social gathering | Moral concern | 0.00 | -0.07 | 0.06 | 0.885 | Point-biserial | 1 |  |
| Almost broken up | ONS count (log) | -0.02 | -0.08 | 0.04 | 0.575 | Point-biserial | 1 |  |
| Date | ONS count (log) | -0.06 | -0.12 | 0.00 | 0.048 | Point-biserial | 1 |  |
| Other context | ONS count (log) | 0.06 | 0.00 | 0.12 | 0.045 | Point-biserial | 1 |  |
| Recently broken up | ONS count (log) | -0.07 | -0.13 | -0.01 | 0.025 | Point-biserial | 1 |  |
| Social gathering | ONS count (log) | -0.06 | -0.12 | 0.00 | 0.060 | Point-biserial | 1 |  |
| Almost broken up | Partner familiarity | 0.05 | 0.02 | 0.13 | 0.212 | Cramer's V | 1 |  |
| In a relationship | Partner familiarity | 0.02 | 0.01 | 0.09 | 0.772 | Cramer's V | 1 |  |
| Met by chance | Partner familiarity | 0.10 | 0.04 | 0.15 | 0.009 | Cramer's V | 1 |  |
| Other context | Partner familiarity | 0.01 | 0.01 | 0.08 | 0.890 | Cramer's V | 1 |  |
| Recently broken up | Partner familiarity | 0.04 | 0.01 | 0.10 | 0.507 | Cramer's V | 1 |  |
| Sex date | Partner familiarity | 0.02 | 0.01 | 0.10 | 0.788 | Cramer's V | 1 |  |
| Almost broken up | Physical disgust | 0.03 | -0.03 | 0.09 | 0.346 | Point-biserial | 1 |  |
| Date | Physical disgust | -0.05 | -0.11 | 0.01 | 0.088 | Point-biserial | 1 |  |
| In a relationship | Physical disgust | -0.01 | -0.07 | 0.05 | 0.837 | Point-biserial | 1 |  |
| Met by chance | Physical disgust | 0.01 | -0.05 | 0.07 | 0.653 | Point-biserial | 1 |  |
| ONS count (log) | Physical disgust | -0.06 | -0.12 | 0.00 | 0.051 | Pearson | 1 |  |
| Other context | Physical disgust | 0.01 | -0.05 | 0.07 | 0.802 | Point-biserial | 1 |  |
| Recently broken up | Physical disgust | 0.05 | -0.01 | 0.11 | 0.115 | Point-biserial | 1 |  |
| Sex date | Physical disgust | -0.02 | -0.08 | 0.04 | 0.543 | Point-biserial | 1 |  |
| Social gathering | Physical disgust | 0.00 | -0.06 | 0.06 | 0.952 | Point-biserial | 1 |  |
| Almost broken up | Pregnancy concern | 0.00 | -0.06 | 0.06 | 0.941 | Point-biserial | 1 |  |
| Date | Pregnancy concern | 0.00 | -0.06 | 0.06 | 0.980 | Point-biserial | 1 |  |
| Met by chance | Pregnancy concern | -0.05 | -0.11 | 0.01 | 0.099 | Point-biserial | 1 |  |
| Other context | Pregnancy concern | -0.03 | -0.09 | 0.03 | 0.294 | Point-biserial | 1 |  |
| Partner familiarity | Pregnancy concern | 0.00 | 0.00 | 0.01 | 0.270 | Eta-squared | 1 |  |
| Recently broken up | Pregnancy concern | -0.06 | -0.12 | 0.01 | 0.074 | Point-biserial | 1 |  |
| Sex date | Pregnancy concern | 0.01 | -0.05 | 0.07 | 0.656 | Point-biserial | 1 |  |
| Social gathering | Pregnancy concern | 0.02 | -0.04 | 0.08 | 0.531 | Point-biserial | 1 |  |
| Almost broken up | Regret | 0.04 | -0.02 | 0.10 | 0.211 | Point-biserial | 1 |  |
| Date | Regret | -0.05 | -0.11 | 0.01 | 0.114 | Point-biserial | 1 |  |
| In a relationship | Regret | 0.04 | -0.02 | 0.10 | 0.218 | Point-biserial | 1 |  |
| Met by chance | Regret | -0.03 | -0.09 | 0.03 | 0.320 | Point-biserial | 1 |  |
| Other context | Regret | -0.03 | -0.09 | 0.03 | 0.304 | Point-biserial | 1 |  |
| Partner familiarity | Regret | 0.00 | 0.00 | 0.01 | 0.099 | Eta-squared | 1 |  |
| Recently broken up | Regret | 0.03 | -0.03 | 0.09 | 0.272 | Point-biserial | 1 |  |
| Social gathering | Regret | 0.01 | -0.05 | 0.07 | 0.734 | Point-biserial | 1 |  |
| Almost broken up | Reputational concern | 0.05 | -0.02 | 0.11 | 0.145 | Point-biserial | 1 |  |
| Met by chance | Reputational concern | -0.06 | -0.12 | 0.00 | 0.071 | Point-biserial | 1 |  |
| ONS count (log) | Reputational concern | -0.06 | -0.12 | 0.00 | 0.050 | Pearson | 1 |  |
| Other context | Reputational concern | -0.08 | -0.14 | -0.01 | 0.015 | Point-biserial | 1 |  |
| Recently broken up | Reputational concern | -0.01 | -0.08 | 0.05 | 0.630 | Point-biserial | 1 |  |
| Sex date | Reputational concern | -0.07 | -0.13 | -0.01 | 0.028 | Point-biserial | 1 |  |
| Social gathering | Reputational concern | 0.07 | 0.01 | 0.13 | 0.026 | Point-biserial | 1 |  |
| Almost broken up | Sexual satisfaction | 0.01 | -0.05 | 0.07 | 0.790 | Point-biserial | 1 |  |
| Date | Sexual satisfaction | 0.06 | 0.00 | 0.12 | 0.068 | Point-biserial | 1 |  |
| Met by chance | Sexual satisfaction | 0.05 | -0.01 | 0.11 | 0.084 | Point-biserial | 1 |  |
| Other context | Sexual satisfaction | 0.00 | -0.06 | 0.07 | 0.886 | Point-biserial | 1 |  |
| Partner familiarity | Sexual satisfaction | 0.00 | 0.00 | 0.01 | 0.328 | Eta-squared | 1 |  |
| Recently broken up | Sexual satisfaction | -0.05 | -0.11 | 0.01 | 0.092 | Point-biserial | 1 |  |
| Social gathering | Sexual satisfaction | 0.02 | -0.04 | 0.08 | 0.458 | Point-biserial | 1 |  |
| Age at ONS | STI concern | 0.02 | -0.04 | 0.08 | 0.485 | Pearson | 1 |  |
| Almost broken up | STI concern | 0.05 | -0.01 | 0.11 | 0.108 | Point-biserial | 1 |  |
| Date | STI concern | 0.01 | -0.05 | 0.07 | 0.685 | Point-biserial | 1 |  |
| In a relationship | STI concern | 0.04 | -0.02 | 0.10 | 0.244 | Point-biserial | 1 |  |
| Met by chance | STI concern | 0.02 | -0.04 | 0.08 | 0.467 | Point-biserial | 1 |  |
| ONS count (log) | STI concern | -0.05 | -0.11 | 0.01 | 0.105 | Pearson | 1 |  |
| Other context | STI concern | 0.03 | -0.03 | 0.09 | 0.278 | Point-biserial | 1 |  |
| Recently broken up | STI concern | -0.04 | -0.10 | 0.02 | 0.203 | Point-biserial | 1 |  |
| Sex date | STI concern | 0.02 | -0.04 | 0.08 | 0.492 | Point-biserial | 1 |  |
| Social gathering | STI concern | -0.07 | -0.13 | -0.01 | 0.017 | Point-biserial | 1 |  |

**Table S3**

*Drug use and subjective intensity of widely consumed substances by participants and partner gender*

| Participant Gender | Women | | | | Men | | | |
| --- | --- | --- | --- | --- | --- | --- | --- | --- |
| Partner Gender | Female | | Male | | Female | | Male | |
|  | n | % | n | % | n | % | n | % |
| Drug influence |  |  |  |  |  |  |  |  |
| Yes | 18 | 64 | 506 | 82 | 249 | 72 | 10 | 21 |
| No | 10 | 36 | 114 | 18 | 99 | 28 | 37 | 79 |
| Alcohol |  |  |  |  |  |  |  |  |
| Yes | 18 | 64 | 500 | 81 | 245 | 70 | 9 | 19 |
| No | 10 | 36 | 120 | 19 | 103 | 30 | 38 | 81 |
| Marijuana |  |  |  |  |  |  |  |  |
| Yes | 2 | 7 | 79 | 13 | 51 | 15 | 3 | 6 |
| No | 26 | 93 | 541 | 87 | 297 | 85 | 44 | 94 |
| MDMA |  |  |  |  |  |  |  |  |
| Yes | 1 | 4 | 13 | 2 | 4 | 1 | 0 | 0 |
| No | 27 | 96 | 607 | 98 | 344 | 99 | 47 | 100 |
| Cocaine |  |  |  |  |  |  |  |  |
| Yes | 0 | 0 | 15 | 2 | 9 | 3 | 1 | 2 |
| No | 28 | 100 | 605 | 98 | 339 | 97 | 46 | 98 |
| Amphetamines |  |  |  |  |  |  |  |  |
| Yes | 0 | 0 | 5 | 1 | 4 | 1 | 0 | 0 |
| No | 28 | 100 | 615 | 99 | 344 | 99 | 47 | 100 |
| Psychedelics |  |  |  |  |  |  |  |  |
| Yes | 0 | 0 | 5 | 1 | 3 | 1 | 0 | 0 |
| No | 28 | 100 | 615 | 99 | 345 | 99 | 47 | 100 |
| Other substances |  | 0 |  |  |  |  |  |  |
| Yes | 1 | 4 | 10 | 2 | 5 | 1 | 0 | 0 |
| No | 27 | 96 | 610 | 98 | 343 | 99 | 47 | 100 |
|  | Mean | SD | Mean | SD | Mean | SD | Mean | SD |
| Intensity overall | 3.17 | 1.20 | 3.21 | 1.20 | 3.09 | 1.21 | 2.60 | 0.97 |
| Intensity alcohol | 4.28 | 1.02 | 4.13 | 1.34 | 4.01 | 1.31 | 3.40 | 1.35 |
| Intensity marijuana | 1.33 | 0.97 | 1.36 | 0.99 | 1.54 | 1.20 | 1.70 | 1.16 |

# Intoxication: Analysis of the Relationship Between Subjective Intoxication and Regret

To determine the optimal functional form for modeling the relationship between subjective intoxication and regret, we conducted a multi-step analysis. Initial testing with Hartigans’ dip test revealed significant multimodality in the subjective intoxication variable (D = 0.087644, *p* < .001), with a subsequent mixture model analysis identifying approximately nine components in the distribution. Given this complex distribution, we systematically compared multiple functional forms to identify which best characterized the relationship between intoxication and regret. We fitted five competing models: linear, quadratic, cubic, logarithmic, and spline regression, and compared then using information criteria (AIC and BIC) and explained variance (R² and adjusted R²) to determine which functional form provided the most parsimonious yet accurate representation of the data.

The quadratic model emerged as the clearly superior functional form. As shown in Table S4, the quadratic model produced the lowest AIC (2603.995) and BIC (2623.771) values, indicating the best balance of fit and parsimony. More complex models (cubic and spline) did not meaningfully improve fit despite their additional parameters, while simpler models (linear and logarithmic) showed substantially poorer fit (e.g., Raftery, 1995^[[2]](#footnote-2)^, p. 139).

Table S4
*Model Comparisons for the Relationship Between Subjective Intoxication and Regret*

| **Model** | **AIC** | **BIC** | **R-squared** | **Adj. R-squared** |
| --- | --- | --- | --- | --- |
| Quadratic | 2603.995 | 2623.771 | 0.1438 | 0.1421 |
| Cubic | 2605.887 | 2630.608 | 0.1438 | 0.1414 |
| Spline | 2607.541 | 2637.205 | 0.1441 | 0.1408 |
| Linear | 2642.060 | 2656.892 | 0.1100 | 0.1092 |
| Log | 2665.340 | 2680.173 | 0.0898 | 0.0889 |

*Note:* Lower AIC and BIC values indicate better model fit.

**Figure S8**

*Histograms of the experiential evaluation items and composite scores*


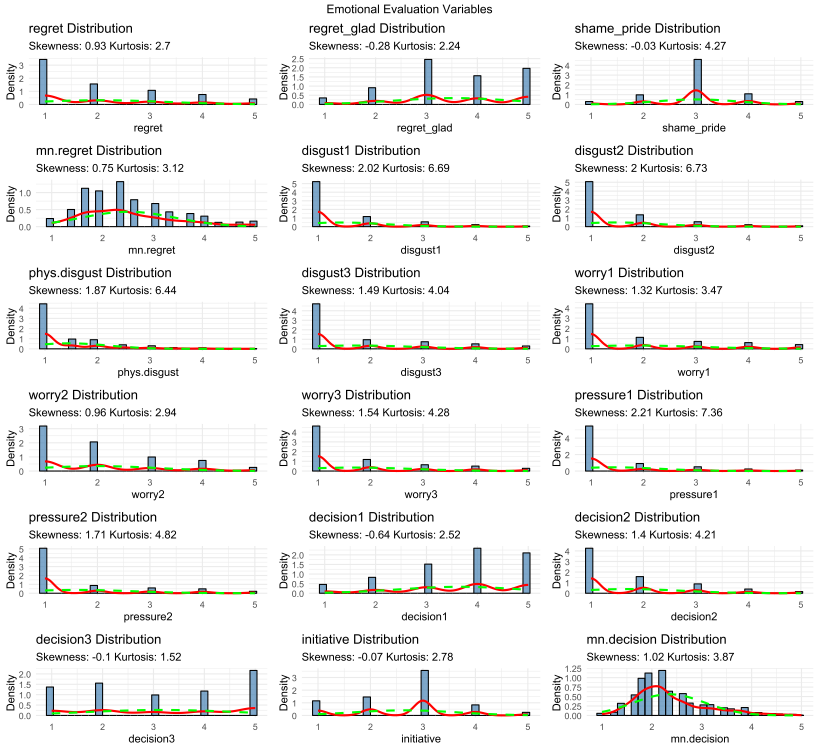


**Figure S9**

*QQ Plots of the experiential evaluation items and composite scores*


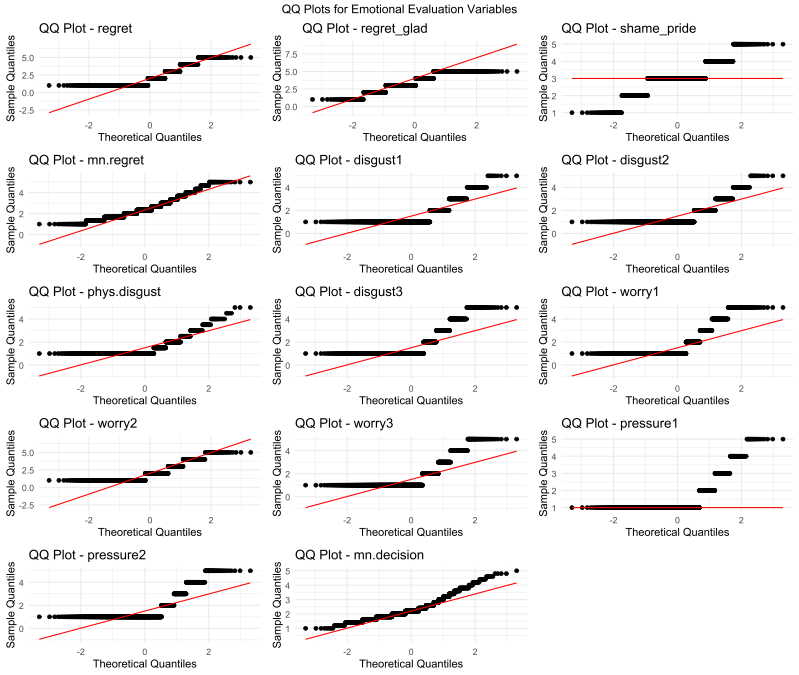


# Distribution of Sexual Experience Items

**Figure S10**

*Histograms plots of the sexual experience items and composite scores*


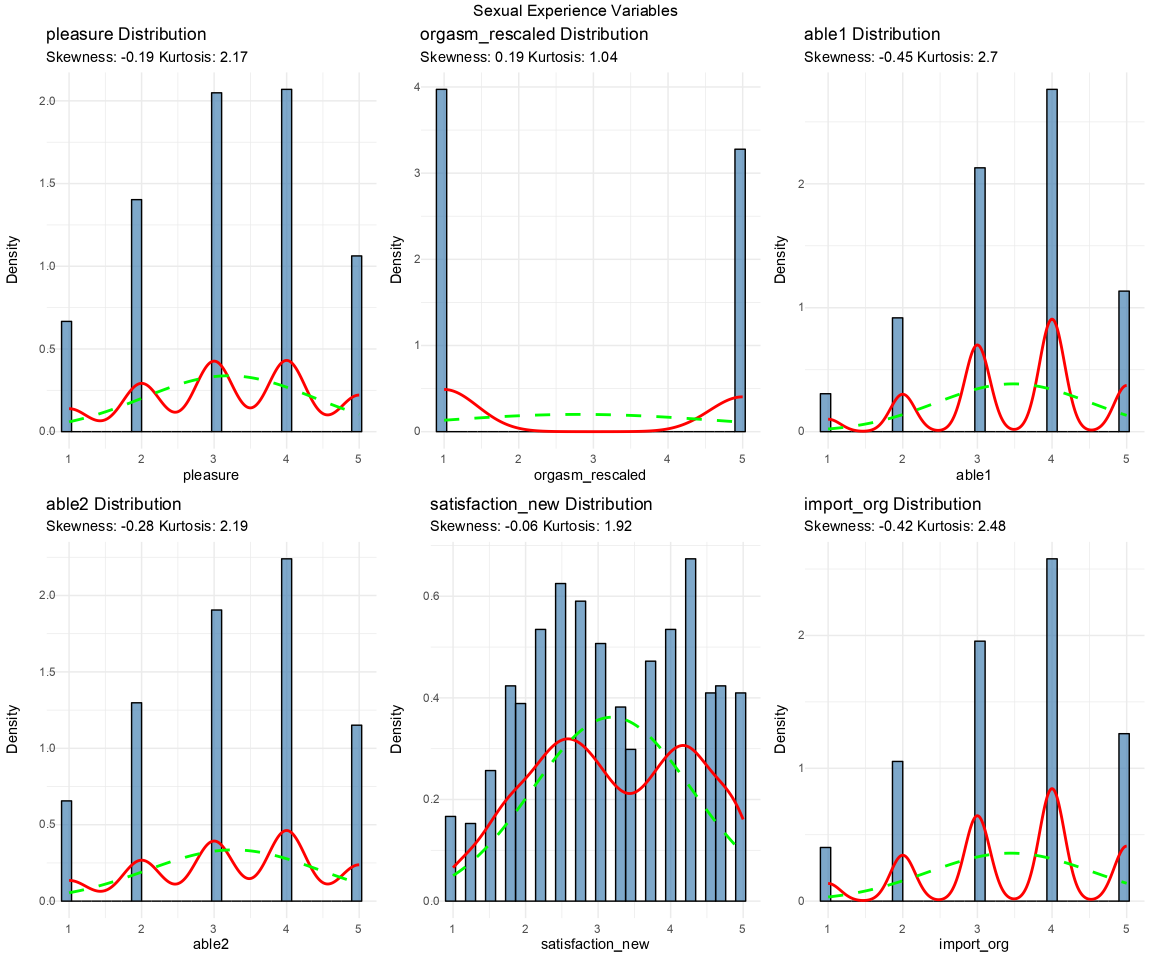


**Figure S11**

*QQ Plots of the sexual experience items and composite scores*


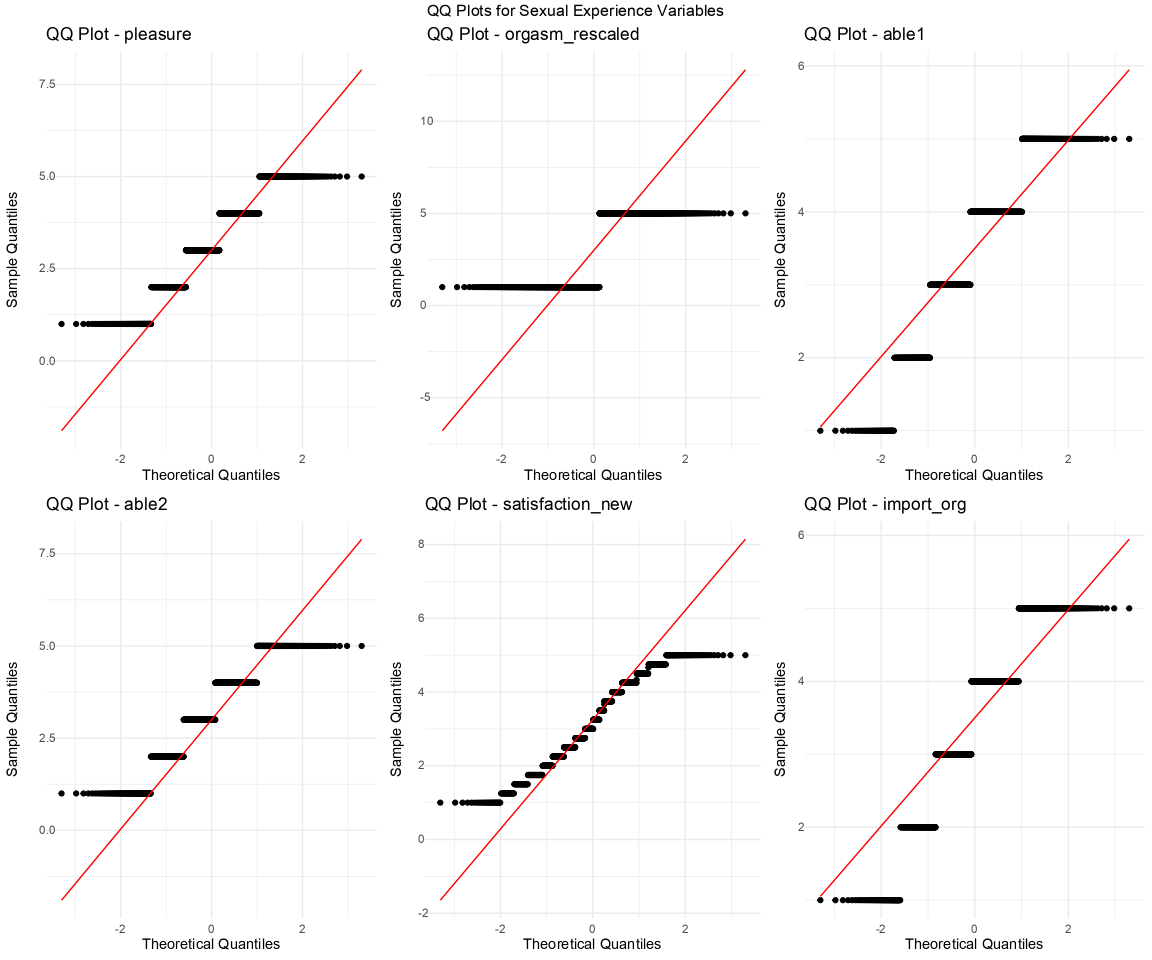


# Zero-order Correlations

**Table S5**

*Zero-Order Correlation of the Main Predictors with the Experiential Factors and Personality Traits*

|  | 1 | 2 | 3 | 4 | 5 | 6 | 7 | 8 |
| --- | --- | --- | --- | --- | --- | --- | --- | --- |
| 1. Gender |  |  |  |  |  |  |  |  |
| 2. ONS Type | 0.14^***^  [0.08, 0.20] |  |  |  |  |  |  |  |
| 3. Regret | -0.25^***^  [-0.30, -0.19] | -0.12^***^  [-0.18, -0.06] |  |  |  |  |  |  |
| 4. Intoxication (ln) | -0.20^***^  [-0.26, -0.14] | -0.21^***^  [-0.27, -0.15] | 0.34^***^  [0.28, 0.39] |  |  |  |  |  |
| 5. Intoxication (squ) | 0.05  [-0.01, 0.11] | 0.13^***^  [0.07, 0.19] | 0.15^***^  [0.09, 0.20] | -0.10^**^  [-0.16, -0.04] |  |  |  |  |
| 6. Satisfaction | 0.44^***^  [0.39, 0.48] | 0.15^***^  [0.09, 0.20] | -0.44^***^  [-0.48, -0.39] | -0.28^***^  [-0.33, -0.22] | -0.03  [-0.09, 0.03] |  |  |  |
| 7. Heteronomy | -0.14^***^  [-0.20, -0.08] | -0.10^***^  [-0.16, -0.04] | 0.50^***^  [0.46, 0.55] | 0.32^***^  [0.27, 0.37] | 0.11^***^  [0.05, 0.17] | -0.29^***^  [-0.35, -0.24] |  |  |
| 8. Phys. Disgust | -0.08^**^  [-0.14, -0.02] | -0.02  [-0.08, 0.04] | 0.36^***^  [0.31, 0.41] | 0.21^***^  [0.15, 0.26] | 0.09^**^  [0.03, 0.15] | -0.22^***^  [-0.27, -0.16] | 0.33^***^  [0.27, 0.38] |  |
| 9. Moral concern | 0.01  [-0.05, 0.07] | -0.08^**^  [-0.14, -0.02] | 0.47^***^  [0.42, 0.51] | 0.19^***^  [0.13, 0.24] | 0.11^***^  [0.05, 0.17] | -0.10^***^  [-0.16, -0.04] | 0.35^***^  [0.29, 0.40] | 0.36^***^  [0.31, 0.41] |
| 10. Pregnancy concern | -0.06^*^  [-0.12, -0.00] | -0.17^***^  [-0.23, -0.11] | 0.13^***^  [0.07, 0.19] | 0.09^**^  [0.03, 0.15] | -0.00  [-0.06, 0.06] | -0.09^**^  [-0.15, -0.03] | 0.10^***^  [0.04, 0.16] | 0.13^***^  [0.07, 0.19] |
| 11. STI concern | -0.04  [-0.10, 0.02] | 0.07^*^  [0.01, 0.13] | 0.16^***^  [0.10, 0.21] | 0.02  [-0.04, 0.08] | 0.10^***^  [0.04, 0.16] | -0.01  [-0.07, 0.05] | 0.13^***^  [0.07, 0.19] | 0.29^***^  [0.23, 0.34] |
| 12. Rep. concern | -0.14^***^  [-0.20, -0.08] | -0.02 [-0.08, 0.04] | 0.44^***^  [0.39, 0.49] | 0.21^***^  [0.15, 0.26] | 0.11^***^  [0.05, 0.17] | -0.15^***^  [-0.21, -0.09] | 0.32^***^  [0.26, 0.37] | 0.26^***^  [0.20, 0.32] |
| 13. Extraversion | -0.07^*^  [-0.13, -0.01] | -0.08^*^ [-0.14, -0.02] | -0.07^*^  [-0.13, -0.01] | 0.13^***^  [0.07, 0.19] | -0.12^***^  [-0.18, -0.06] | -0.02  [-0.09, 0.04] | -0.06  [-0.12, 0.01] | -0.08^**^  [-0.14, -0.02] |
| 14. Agreeableness | -0.12^***^  [-0.18, -0.06] | -0.03 [-0.09, 0.03] | 0.00  [-0.06, 0.06] | 0.06^*^  [0.00, 0.12] | -0.09^**^  [-0.15, -0.03] | -0.06^*^  [-0.12, -0.00] | 0.04  [-0.03, 0.10] | -0.06  [-0.12, 0.00] |
| 15. Conscientiousness | -0.06^*^  [-0.12, -0.00] | -0.05 [-0.11, 0.01] | -0.03  [-0.09, 0.03] | -0.08^*^  [-0.14, -0.02] | -0.00  [-0.06, 0.06] | 0.02  [-0.04, 0.08] | -0.05  [-0.11, 0.01] | -0.09^**^  [-0.15, -0.03] |
| 16. Em. stability | 0.21^***^  [0.15, 0.27] | -0.04 [-0.11, 0.02] | -0.20^***^  [-0.26, -0.14] | -0.05  [-0.11, 0.01] | -0.05  [-0.11, 0.01] | 0.19^***^  [0.14, 0.25] | -0.17^***^  [-0.23, -0.11] | -0.20^***^  [-0.25, -0.14] |
| 17. Openness | -0.10^**^  [-0.16, -0.04] | 0.02 [-0.05, 0.08] | -0.13^***^  [-0.18, -0.07] | 0.04  [-0.03, 0.10] | -0.07^*^  [-0.13, -0.01] | 0.01  [-0.05, 0.07] | -0.08^*^  [-0.14, -0.02] | -0.03  [-0.09, 0.03] |

|  | 9 | 10 | 11 | 12 | 13 | 14 | 15 | 16 |
| --- | --- | --- | --- | --- | --- | --- | --- | --- |
| 10. Pregnancy concern | 0.13^***^  [0.07, 0.19] |  |  |  |  |  |  |  |
| 11. STI concern | 0.15^***^  [0.09, 0.21] | 0.37^***^  [0.32, 0.42] |  |  |  |  |  |  |
| 12. Rep. concern | 0.47^***^  [0.42, 0.52] | 0.19^***^  [0.13, 0.25] | 0.18^***^  [0.13, 0.24] |  |  |  |  |  |
| 13. Extraversion | -0.07^*^  [-0.13, -0.01] | -0.05  [-0.11, 0.01] | -0.09^**^  [-0.15, -0.03] | -0.06^*^  [-0.12, -0.00] |  |  |  |  |
| 14. Agreeableness | -0.07^*^  [-0.13, -0.01] | -0.01  [-0.07, 0.05] | -0.07^*^  [-0.13, -0.00] | 0.01  [-0.05, 0.07] | 0.03  [-0.03, 0.09] |  |  |  |
| 15. Conscientiousness | -0.03  [-0.09, 0.03] | -0.05  [-0.11, 0.01] | -0.03  [-0.09, 0.03] | 0.02  [-0.04, 0.08] | 0.02  [-0.04, 0.08] | 0.18^***^  [0.12, 0.24] |  |  |
| 16. Em. stability | -0.10^**^  [-0.16, -0.04] | -0.10^**^  [-0.16, -0.04] | -0.11^***^  [-0.17, -0.05] | -0.11^***^  [-0.17, -0.05] | 0.26^***^  [0.20, 0.32] | 0.16^***^  [0.10, 0.22] | 0.27^***^  [0.22, 0.33] |  |
| 17. Openness | -0.14^***^  [-0.20, -0.08] | -0.05  [-0.11, 0.01] | -0.01  [-0.07, 0.05] | -0.09^**^  [-0.15, -0.03] | 0.33^***^  [0.27, 0.38] | 0.20^***^  [0.14, 0.26] | 0.06^*^  [0.00, 0.12] | 0.16^***^  [0.10, 0.22] |

*Note.* Pearson correlation coefficients with 95% confidence intervals in brackets. Significance levels are indicated as follows: ^*^ *p* < .05, ^**^ *p* < .01, ^***^ *p* < .001 (all Holm-corrected).

**Figure S12**

*Zero-order correlations of regret with the mediating variables and the Big Five*


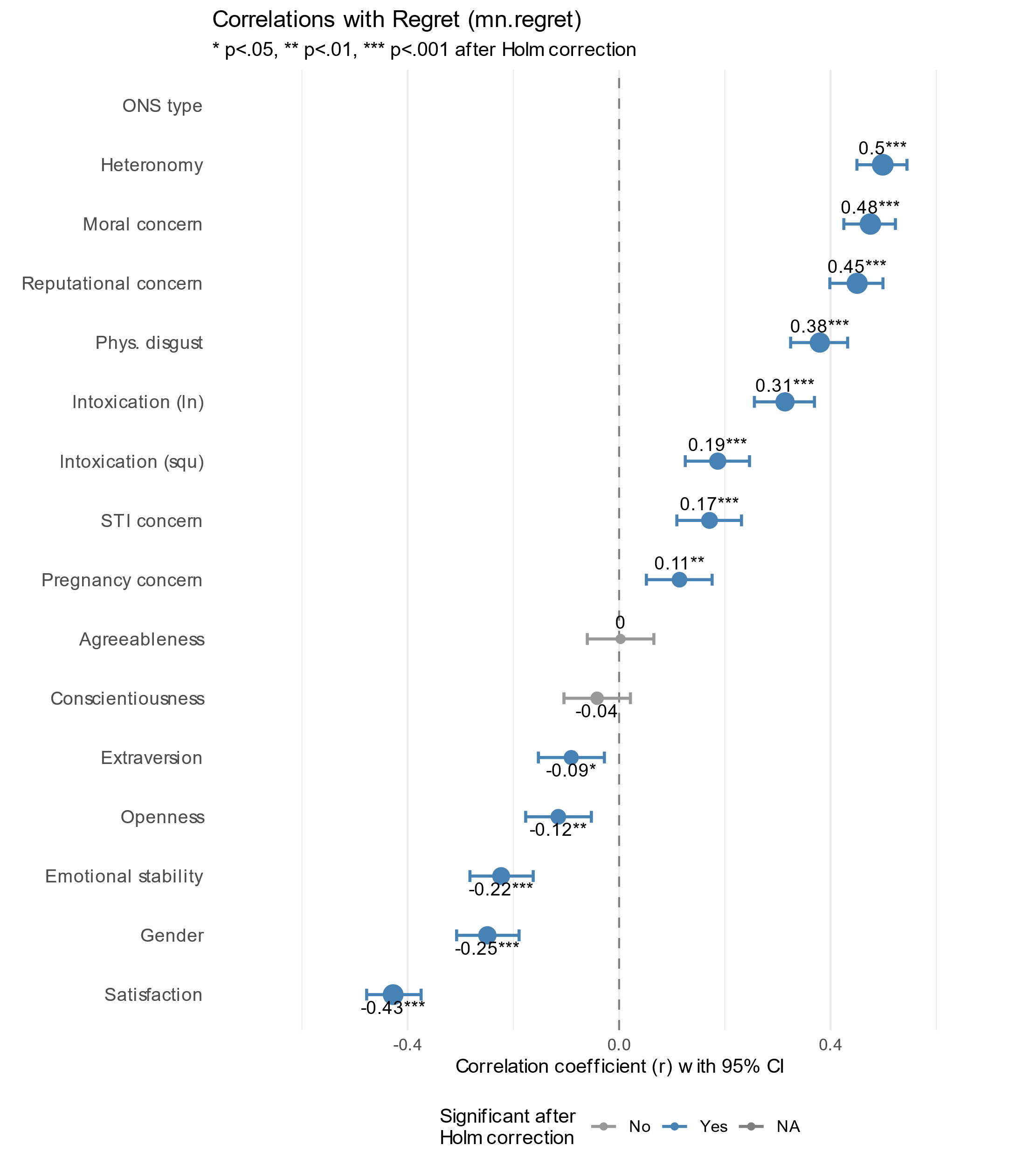


**Table S6**

*Zero-order correlations of the mediating variables with regret by ONS type*

| Heterosexual ONS (n = 969) | | | |
| --- | --- | --- | --- |
| Variable | r | Raw p value | Adjusted p value |
| Heteronomy | 0.50 | 0.000 | 0.000 |
| Moral concern | 0.48 | 0.000 | 0.000 |
| Reputation concern | 0.45 | 0.000 | 0.000 |
| Satisfaction | -0.43 | 0.000 | 0.000 |
| Phys. Disgust | 0.38 | 0.000 | 0.000 |
| Intoxication (lin) | 0.31 | 0.000 | 0.000 |
| Gender | -0.25 | 0.000 | 0.000 |
| Emotional stability | -0.22 | 0.000 | 0.000 |
| Intoxication (squ) | 0.19 | 0.000 | 0.000 |
| STO concern | 0.17 | 0.000 | 0.000 |
| Pregnancy concern | 0.11 | 0.000 | 0.002 |
| Openness | -0.12 | 0.000 | 0.002 |
| Extraversion | -0.09 | 0.005 | 0.015 |
| Conscientiousness | -0.04 | 0.196 | 0.392 |
| Agreeableness | 0.00 | 0.928 | 0.928 |
|  |  |  |  |
|  |  |  |  |
| Same-sex ONS (n = 75) | | | |
| Variable | r | Raw p value | Adjusted p value |
| Heteronomy | 0.39 | 0.001 | 0.008 |
| Satisfaction | -0.38 | 0.000 | 0.001 |
| Intoxication (lin) | 0.38 | 0.001 | 0.013 |
| Openness | -0.30 | 0.010 | 0.122 |
| Reputation concern | 0.29 | 0.012 | 0.135 |
| Moral concern | 0.22 | 0.058 | 0.580 |
| STO concern | 0.07 | 0.567 | 1 |
| Phys. Disgust | 0.02 | 0.836 | 1 |
| Extraversion | 0.01 | 0.960 | 1 |
| Emotional stability | -0.03 | 0.792 | 1 |
| Pregnancy concern | -0.04 | 0.734 | 1 |
| Conscientiousness | -0.08 | 0.496 | 1 |
| Agreeableness | -0.09 | 0.436 | 1 |
| Gender | -0.10 | 0.394 | 1 |
| Intoxication (squ) | -0.11 | 0.342 | 1 |

**Figure S13**

*Zero-order correlations of gender, ONS type and regret with the experiential factors on item level*


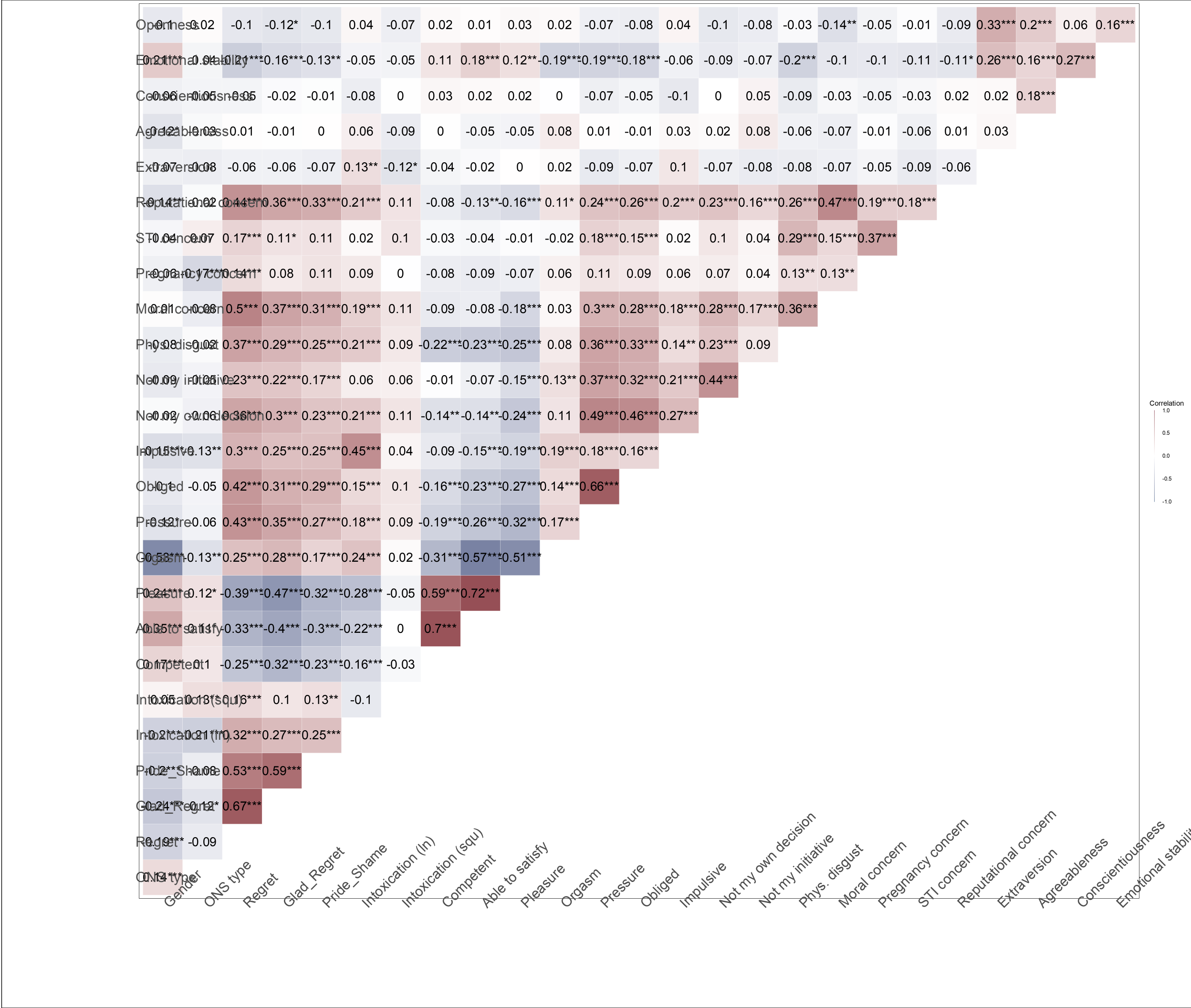


*Note.* Correlation coefficients were Holm-corrected for multiple testing. Gender was coded -1 = female, 1 = male. Positive correlations with gender thus mean higher values for men in the respective variable; negative correlations mean higher values for women.

Contrary to previous research (e.g., Kennair et al., 2018), we did not find men to report taking the initiative to engage in the ONS more often than did women. Of the heteronomy variables, women reported making a more impulsive decision and feeling slightly more pressured. The largest gender difference emerged for sexual gratification items; most notably for orgasm achievement, replicating many prior findings. This was followed by the partner’s ability to satisfy, and sexual pleasure experienced, while competence of the partner showed weaker correlations.

# Heteroscedasticity due to uneven group size

**Figure S14**

*Residual plots*

**
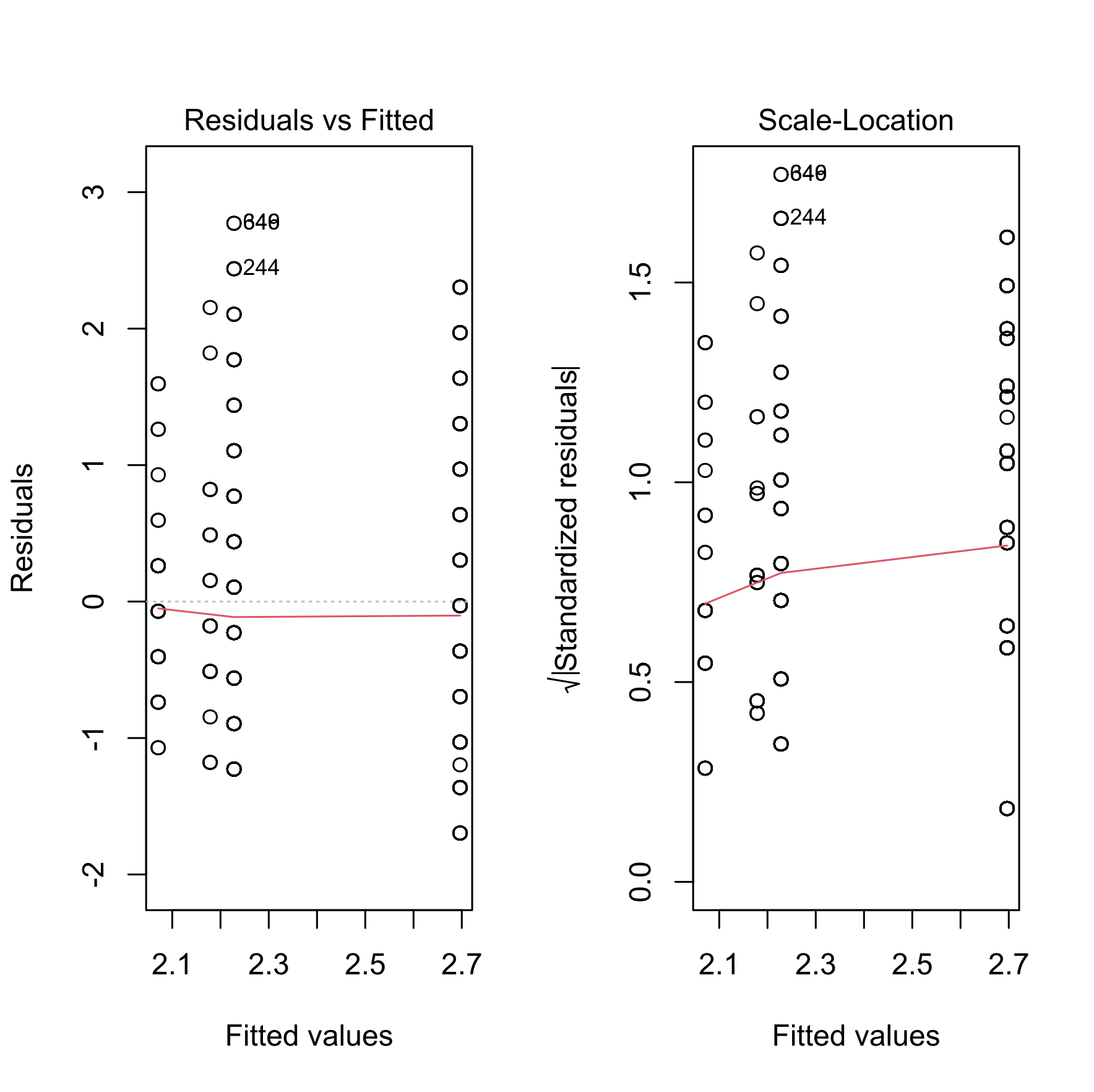
**

Due to heteroscedasticity, we used HC3 robust standard errors in linear regressions and MLR estimation in SEMs. A comparison of the two linear regressions showed that the results were highly similar, but that due to smaller standard errors, the CIs and the p-values were reduced.

The following table presents an overview of the standard and the robust regression model.

|  | Dependent Variable | |
| --- | --- | --- |
|  | Regret score | |
|  | Standard SE | HC3 Robust SE |
| Gender | -0.157*** | -0.157*** |
|  | (0.055) | (0.049) |
|  |  |  |
| ONS Type | -0.160*** | -0.160*** |
|  | (0.055) | (0.049) |
|  |  |  |
| Gender * ONS Type (Partner gender) | 0.082 | 0.082* |
|  | (0.055) | (0.049) |
|  |  |  |
| Constant | 2.307*** | 2.307*** |
|  | (0.055) | (0.049) |
|  |  |  |
| Observations | 1,037 | 1,037 |
| R2 | 0.072 | 0.072 |
| Adjusted R2 | 0.069 | 0.069 |
| Residual Std. Error (df = 1033) | 0.882 | 0.882 |
| F Statistic (df = 3; 1033) | 26.712*** | 26.712*** |

Standardized Parameter Estimates

| Parameter | Std. Coef. | 95% CI |
| --- | --- | --- |
|  |  |  |
| (Intercept) | -0.22 | [-0.34, -0.10] |
| Gender | -0.17 | [-0.29, -0.05] |
| ONS Type | -0.17 | [-0.29, -0.06] |
| Gender * ONS Type (Partner gender) | 0.09 | [-0.03,  0.21] |

# Reasons for Moral Concern

Although moral concern did not correlate with gender, it was a strong correlate of regret and thus we analyzed its distribution and the reasons for moral concern in more detail (Figure S12 and Figure S13). As there were not enough individuals who reported moral concern after same-sex ONS, we included only participants in heterosexual ONS in this analysis who endorsed specific reasons (225 women, 122 men). We conducted a series of chi-square tests with Holm-adjusted p-values to control for multiple comparisons, comparing the proportion of each gender.

Men and women showed notable differences in their patterns of moral concern. The most pronounced difference emerged for hurting the self, with women significantly more likely than men to report having hurt themselves by engaging in the ONS (*p* = .000, Φ = 0.25). We observed a trend toward gender difference in concerns about cheating, with more men than women citing this as a reason for moral concern (*p* = .058, Φ = 0.13). Similarly, more men than women expressed concern about hurting others, though this difference did not reach statistical significance after correction (*p* = .154, Φ = 0.10). No meaningful gender differences emerged for concerns about the ONS involving the “wrong person” (*p* = .916) or other unspecified reasons (30.7% women vs. 25.4% men, *p* = .728). Further proportion comparison by relationships status at the time of the ONS and moral concern are reported in Figure S14.

**Figure S15**

*Distribution of Moral Concerns by Gender and ONS Type*

*
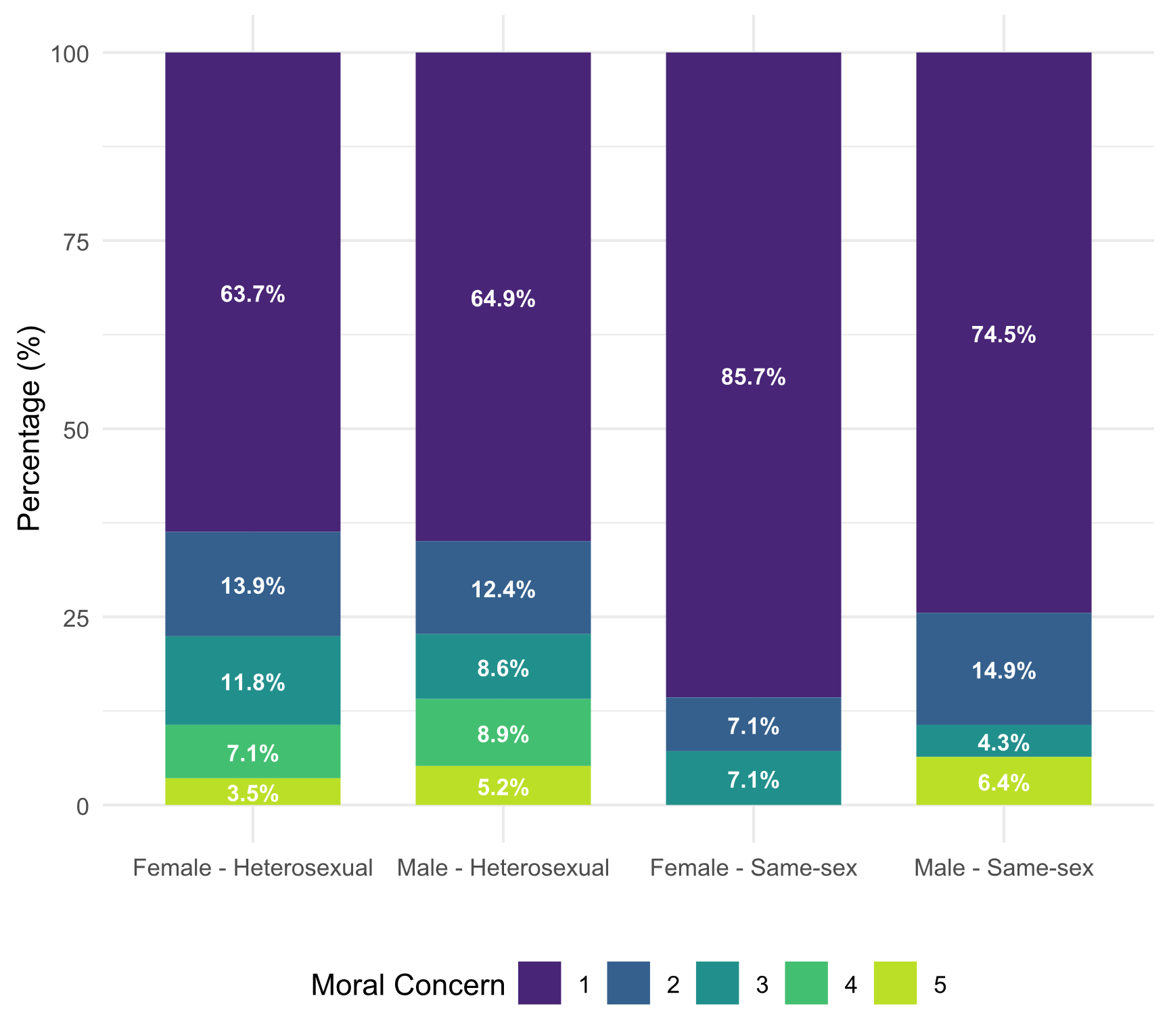
*

*Note.* This figure displays the extent of moral concern about the ONS by gender and type of sexual encounter. In each group, most participants reported no moral concerns at all about their ONS experience, while the distribution varies notably by ONS type. Same-sex ONS were reported as less morally concerning than heterosexual ONS. Female same-sex ONS had the lowest moral concerns. Heterosexual ONS showed more similar patterns between genders.

**Figure S16**

*Reasons for Moral Concerns by Gender and ONS Type*


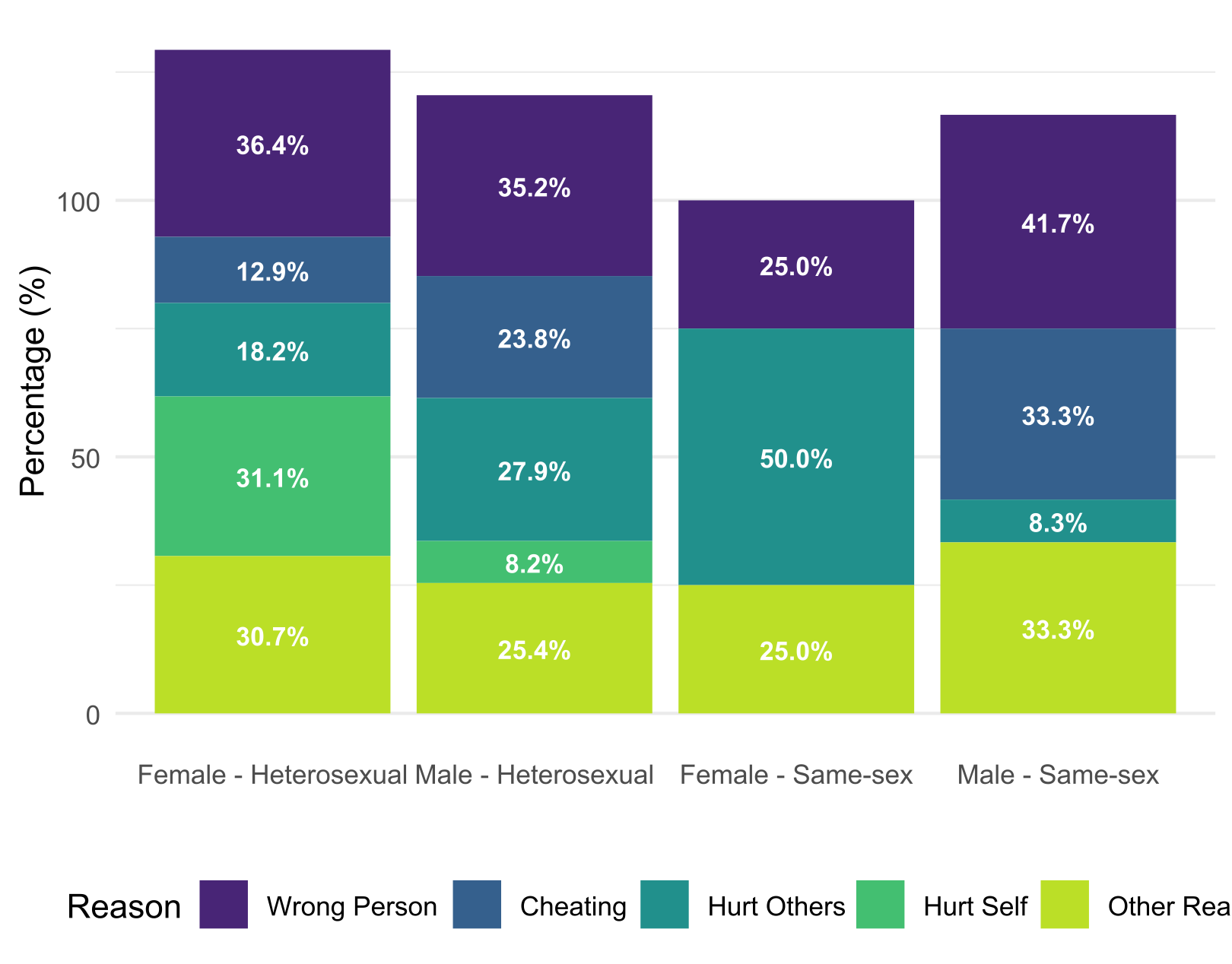


*Note.* This figure visualizes the percentage distribution of reasons selected for moral concern by gender and ONS type. Participants who indicated at least some moral concern (> 1, see Figure S12) were asked to select all reasons for that concern that apply. Note that there were only four women and 12 men in same-sex ONS who reported moral concern. The proportions for same-sex ONS are thus not reliable. All reasons specified in the “Other” category are listed in Table S5.

**Figure S17**

*Reasons for moral concern by ONS type and relationship status*


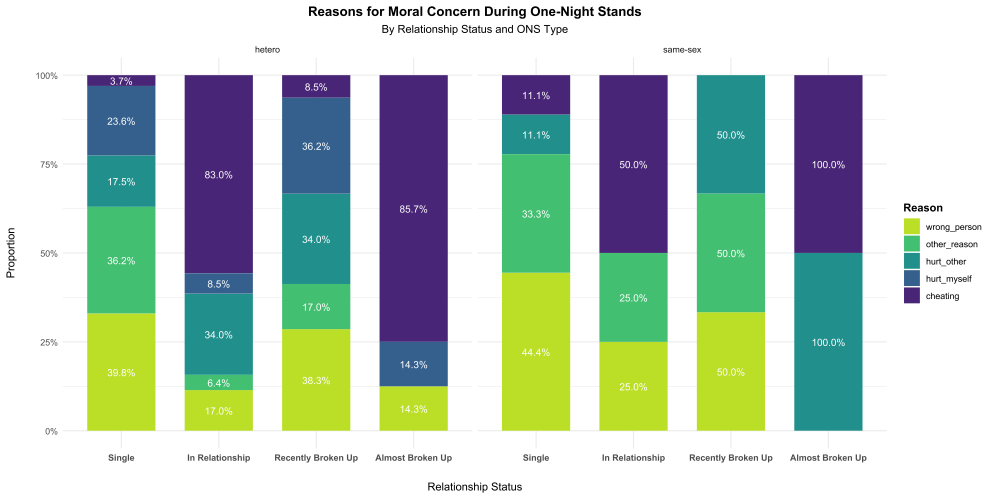


**Table S7**

*Reasons indicated for moral concern as an open text response*

| **ID** | **Reason (original language)** | **Reason (English translation)** |
| --- | --- | --- |
| 412 | 14 Jahre älter | 14 years older |
| 251 | absolut keine Gefühle | absolutely no feelings |
| 692 | Eigentlich gegen meine damaligen Prinzipien | Actually against my principles at the time |
| 413 | Eigentlich nicht mein typ | Actually not my type |
| 332 | Alter und Beziehungsstatus des Gegenübers | Age and relationship status of the counterpart |
| 361 | altersunterschied | age difference |
| 948 | Altersunterschied | Age difference |
| 255 | Einstellung zu ONS - gesellschaftlicher druck | Attitude towards ONS - societal pressure |
| 1109 | großer Altersunterschied | big age difference |
| 1103 | kompliziertes Netzwerk aus Freunden/Bekanntschaften | Complicated network of friends/acquaintances |
| 1018 | konservative Erziehung | Conservative upbringing |
| 584 | Cousine eines Freundes | Cousin of a friend |
| 793 | crush eines freundes | crush of a friend |
| 202 | Habe ihn zu wenig gekannt. | Didn't know him well enough |
| 733 | entfernter Verwandter | distant relative |
| 907 | Betrunken | Drunk |
| 1025 | aufgrund meiner Periode wollte ich eigentlich keinen Sex, trotz mehrmaligem Äußern hörte er nicht auf mich anzufassen. Schließlich ließ ich mich darauf ein, weil ich es wollte. Unmoralisch hat sich angefühlt es zu tun obwohl es ihn nicht interessiert hat | Due to my period, I actually didn't want to have sex. Despite expressing this multiple times, he didn't stop touching me. Eventually, I gave in because I wanted it. It felt immoral to do it even though he didn't care. |
| 930 | Erwartungen haben sich nicht erfÃ¼llt | Expectations were not fulfilled |
| 725 | Ausnutzen, von anderer Person loskommen | Exploiting, getting away from another person |
| 830 | Erstes Mal | First time |
| 716 | aus den falschen Gründen | for the wrong reasons |
| 1045 | er war vergeben und es haben alle Freunde, die auf der Party waren, mitbekommen | he was in a relationship, and all the friends at the party noticed |
| 167 | Er war verheiratet und hatte Kinder | He was married and had children |
| 448 | Er war nicht wirklich mein Typ | He wasn’t really my type |
| 656 | Meinen Stolz verletzt | Hurt my pride |
| 82 | Ich habe mich selber enttäuscht mit meiner Wahl | I disappointed myself with my choice. |
| 880 | ich hatte zu dem Zeitpunkt eine Geschlechtskrankheit und war so betrunken, dass ich mich kaum noch daran erinnern kann | I had a sexually transmitted disease at the time and was so drunk that I can hardly remember it. |
| 1031 | ich stand unter drogen und habe mich danach nicht mehr gemeldet (habe aber nicht gelogen o. Ä. um zum ONS zu kommen) | I was on drugs and didn’t get in touch afterwards (but I didn’t lie or anything to get to the ONS). |
| 972 | Ich traf mich zu der Zeit mit einer weiteren Person, die jedoch nichts von der Sexpartnerin wusste | I was seeing another person at the time, who didn’t know about the [female] sex partner. |
| 781 | Ich wurde damit sehr verletzt, da ich am nächsten Tag erfuhr, dass er jemanden Anders sehr gerne hat. Ich fühlte mich sehr verarscht und verletzt, da er sowohl Anzeichen mir gegenüber gemacht hat, mich sehr gerne zu mögen – Gefühl von Ausnutzung | I was very hurt by it because I found out the next day that he really liked someone else. I felt very deceived and hurt because he had shown signs of liking me very much – feeling of exploitation. |
| 790 | In einem fremden Bett | In a stranger’s bed. |
| 328 | In der öffentlichkeit bei einer Party | In public at a party. |
| 198 | Im Nachhinein fühlte ich mich schlecht. (Habe zwar Bestätigung bekommen habe, war aber noch nicht aber meinen Ex-Partner hinweg und habe den Sex einfach über mich ergehen lassen, auch wenn ich es eigentlich gar nicht mehr wollte.) | In retrospect, I felt bad. (Although it was good for my ego, I was not yet over my ex-partner and just endured the sex, even though I didn’t really want it anymore.) |
| 576 | Anfangs bestand kein Interesse | Initially there was no interest |
| 214 | es fühlt sich später falsch an, weil der push fürs ego wichtiger war als alles andere. und der verfliegt | it feels wrong later because the push for the ego was more important than anything else. And that fades away. |
| 833 | Ist immer noch verrufen | It is still disreputable. |
| 3 | Es hätte nicht sein müssen. Wurde von ihm überredet. Hätte bei NEIN bleiben sollen ... | It shouldn’t have happened. Was persuaded by him. Should have stayed with NO |
| 495 | Es war einfach eine unnötige Aktion | It was just an unnecessary action |
| 861 | Keine Gefühle und wenig Interesse an Person selbst | No feelings and little interest in the person |
| 272 | Keiner | None |
| 633 | gar nicht | Not at all |
| 268 | andere Person war mit im Zimmer; Schamempfinden aber aufgrund Alkoholeinfluss in dem Moment nicht gegeben | other person was in the room; shame was not present due to alcohol influence |
| 891 | heimlich, mit anderen Personen im selben Raum | Secretly, with another person in the room |
| 87 | andere Person dafür versetzt | Stooped up other person for it |
| 933 | Fremde Person + erstes Mal | Stranger + first time |
| 892 | der Typ war 50 und hatte ne Frau und 2 Kinder, und ja das hat er mir vor der Action erzählt | the guy was 50 and had a wife and 2 kids, and yes he told me that before the action |
| 109 | Der Typ war in einer Beziehung, habe ich erst danach erfahren. | The guy was in a relationship, I only found out afterwards |
| 254 | 3er | Threesome |
| 204 | hat in einem Hostelzimmer stattgefunden wo währenddessen andere geschlafen haben und man eigentlich auch niemanden mitnehmen durfte... | took place in a hostel room where others were sleeping and you weren’t actually allowed to bring anyone... |
| 290 | hat probiert mich zu sachen zu überreden die ich nicht wollte | tried to persuade me to do things I didn’t want |
| 238 | mit Familie gut bekannt | Well known to the family |
| 10 | falscher Zeitpunkt | wrong timing |
| 1251 | Get got really dirty and sexual and taboos were discussed |  |
| 1176 | Grew up catholic, cant shake some of the things I had been told were wrong |  |
| 1345 | he coaxed me into doing it when i didn't really want to |  |
| 1260 | I firstly said no. |  |
| 1358 | I knew I would hurt this person by doing this as I could not change my stance on the relationship between us. |  |
| 1158 | I read the question as in it felt wrong . besides not feeling confident in my decision, it wasn't wrong as in ceating or smthn, we were both singles and viewed it as casual |  |
| 1392 | I shouldnt give myself sexually to people I’m not committed to emotionally |  |
| 1406 | I'm not sure, but it felt wrong like I didn't really want it |  |
| 1330 | It is not safe |  |
| 1319 | it seemed meaningless |  |
| 1299 | It was a meaningless stranger. Very uneducated girl, boring in bed and outside. |  |
| 1370 | It was very kinky |  |
| 1241 | just a wrong decision, no negative consequences that I was aware of, but I was disappointed in myself and the other person. I felt, and continue to feel it was a gross, unpleasant experience I simply should have avoided |  |

Analysis of participants’ moral concerns regarding casual sexual encounters revealed several distinct thematic categories, some of which matched the evaluation of the ONS (e.g., decision heteronomy) or the existing reason categories. Indeed, many participants selected both a specific reason and the “other” category, possibly to provide more information on their concern. As such, relationship status issues were frequently cited, including partners who were married or in relationships, as well as complications from participants’ own relationship situations or social network connections.

Decision quality concerns encompassed regret about choices made, lack of genuine interest in partners, and perceptions of encounters as meaningless or motivated by ego rather than an authentic connection. Consent and pressure dynamics were reported by multiple participants, including feelings of being coerced or persuaded despite initial reluctance. Intoxication was noted as a complicating factor affecting judgment in several accounts.

Contextual factors such as inappropriate settings or the presence of others nearby constituted another category of concern. Religious backgrounds and cultural values influenced moral evaluations, as did significant age differences between partners.

Physical and health considerations included unsafe practices and (giving) sexually transmitted infections. Emotional impact was cited by some participants, particularly feelings of self-disappointment or exploitation. Sexual content concerns were mentioned in relation to unconventional activities or multiple partners.

# Structural equation modelling – detailed path statistics

**Table S8**

*Parallel SEM including emotional stability as a covariate*

| **Criterion** | **Predictor** | **b** | **SE** | **z** | **p** | **LLCI** | **ULCI** | **β** |
| --- | --- | --- | --- | --- | --- | --- | --- | --- |
| Regret | Gender | -0.01 | 0.04 | -0.28 | .782 | -0.09 | 0.07 | -0.01 |
| Regret | ONS type | -0.06 | 0.04 | -1.60 | .110 | -0.14 | 0.01 | -0.03 |
| Regret | Gender × ONS type | 0.01 | 0.04 | 0.38 | .701 | -0.06 | 0.09 | 0.02 |
| Regret | Emotional stability | -0.04 | 0.02 | -2.24 | .025 | -0.07 | -0.01 | -0.06 |
| Satisfaction | Gender | 0.36 | 0.06 | 5.77 | < .001 | 0.24 | 0.49 | 0.32 |
| Satisfaction | ONS type | 0.22 | 0.06 | 3.61 | < .001 | 0.10 | 0.34 | 0.10 |
| Satisfaction | Gender × ONS type | -0.11 | 0.06 | -1.73 | .083 | -0.23 | 0.01 | -0.09 |
| Satisfaction | Emotional stability | 0.09 | 0.02 | 3.85 | < .001 | 0.04 | 0.13 | 0.11 |
| Heteronomy | Gender | -0.02 | 0.04 | -0.44 | .657 | -0.09 | 0.05 | -0.02 |
| Heteronomy | ONS type | -0.15 | 0.04 | -4.24 | < .001 | -0.21 | -0.08 | -0.11 |
| Heteronomy | Gender × ONS type | 0.07 | 0.04 | 1.86 | .063 | -0.01 | 0.13 | 0.09 |
| Heteronomy | Emotional stability | -0.08 | 0.02 | -4.43 | < .001 | -0.11 | -0.04 | -0.15 |
| Intoxication (ln) | Gender | -0.45 | 0.09 | -5.18 | < .001 | -0.63 | -0.28 | -0.29 |
| Intoxication (ln) | ONS type | -0.51 | 0.09 | -5.76 | < .001 | -0.68 | -0.33 | -0.17 |
| Intoxication (ln) | Gender × ONS type | -0.22 | 0.09 | -2.50 | .012 | -0.39 | -0.05 | -0.14 |
| Intoxication (ln) | Emotional stability | -0.03 | 0.03 | -0.83 | .407 | -0.09 | 0.04 | -0.03 |
| Intoxication (squ) | Gender | 0.30 | 0.12 | 2.57 | .010 | 0.07 | 0.53 | 0.15 |
| Intoxication (squ) | ONS type | 0.39 | 0.12 | 3.36 | .001 | 0.16 | 0.62 | 0.11 |
| Intoxication (squ) | Gender × ONS type | 0.26 | 0.11 | 2.22 | .026 | 0.03 | 0.48 | 0.13 |
| Intoxication (squ) | Emotional stability | -0.07 | 0.04 | -1.69 | .091 | -0.15 | 0.01 | -0.05 |
| Reputational concern | Gender | -0.04 | 0.07 | -0.63 | .532 | -0.17 | 0.08 | -0.04 |
| Reputational concern | ONS type | -0.05 | 0.07 | -0.72 | .474 | -0.17 | 0.09 | -0.02 |
| Reputational concern | Gender × ONS type | 0.11 | 0.07 | 1.76 | .079 | -0.02 | 0.24 | 0.10 |
| Reputational concern | Emotional stability | -0.07 | 0.02 | -2.81 | .005 | -0.12 | -0.02 | -0.09 |
| Regret | Satisfaction | -0.21 | 0.02 | -8.71 | < .001 | -0.26 | -0.16 | -0.25 |
| Regret | Heteronomy | 0.36 | 0.04 | 9.31 | < .001 | 0.28 | 0.44 | 0.28 |
| Regret | Intoxication (ln) | 0.07 | 0.02 | 4.26 | < .001 | 0.04 | 0.10 | 0.11 |
| Regret | Intoxication (squ) | 0.04 | 0.01 | 3.55 | < .001 | 0.02 | 0.07 | 0.09 |
| Regret | Reputational concern | 0.22 | 0.02 | 9.01 | < .001 | 0.17 | 0.27 | 0.27 |

| **Pathway** | **b** | **SE** | **z** | **p** | **LLCI** | **ULCI** | **β** |
| --- | --- | --- | --- | --- | --- | --- | --- |
| Indirect: Gender → Satisfaction → Regret (Hetero) | -0.10 | 0.01 | -7.28 | < .001 | -0.13 | -0.07 | -0.11 |
| Indirect: Gender → Heteronomy → Regret (Hetero) | -0.03 | 0.01 | -3.35 | .001 | -0.05 | -0.01 | -0.03 |
| Indirect: Gender → Intoxication (ln) → Regret (Hetero) | -0.02 | 0.01 | -3.15 | .002 | -0.03 | -0.01 | -0.02 |
| Indirect: Gender → Intoxication (squ) → Regret (Hetero) | 0.00 | 0.00 | 0.63 | .530 | 0.00 | 0.01 | 0.00 |
| Indirect: Gender → Reputational concern → Regret (Hetero) | -0.03 | 0.01 | -4.11 | < .001 | -0.05 | -0.02 | -0.04 |
| Indirect: Gender → Satisfaction → Regret (Same-sex) | -0.05 | 0.03 | -2.03 | .042 | -0.11 | 0.00 | -0.06 |
| Indirect: Gender → Heteronomy → Regret (Same-sex) | 0.02 | 0.02 | 0.74 | .460 | -0.03 | 0.07 | 0.02 |
| Indirect: Gender → Intoxication (ln) → Regret (Same-sex) | -0.05 | 0.02 | -2.89 | .004 | -0.08 | -0.02 | -0.05 |
| Indirect: Gender → Intoxication (squ) → Regret (Same-sex) | 0.02 | 0.01 | 1.96 | .050 | 0.00 | 0.05 | 0.03 |
| Indirect: Gender → Reputational concern → Regret (Same-sex) | 0.02 | 0.03 | 0.58 | .559 | -0.04 | 0.07 | 0.02 |
| Total indirect effect (Hetero) | -0.18 | 0.02 | -8.23 | < .001 | -0.22 | -0.13 | -0.19 |
| Total indirect effect (Same-sex) | -0.04 | 0.06 | -0.69 | .487 | -0.16 | 0.07 | -0.04 |
| Total effect (Hetero) | -0.20 | 0.03 | -6.75 | < .001 | -0.26 | -0.14 | -0.21 |
| Total effect (Same-sex) | -0.04 | 0.09 | -0.40 | .691 | -0.22 | 0.14 | -0.04 |
| Moderated mediation: Satisfaction pathway | 0.05 | 0.03 | 1.69 | .090 | 0.00 | 0.10 | 0.05 |
| Moderated mediation: Heteronomy pathway | 0.05 | 0.03 | 1.81 | .070 | 0.00 | 0.10 | 0.05 |
| Moderated mediation: Intoxication (ln) pathway | -0.03 | 0.01 | -2.12 | .034 | -0.06 | -0.01 | -0.03 |
| Moderated mediation: Intoxication (squ) pathway | 0.02 | 0.01 | 1.81 | .070 | 0.00 | 0.05 | 0.02 |
| Moderated mediation: Reputational concern pathway | 0.05 | 0.03 | 1.74 | .082 | -0.01 | 0.11 | 0.05 |
| Moderated mediation: Total index | 0.14 | 0.06 | 2.25 | .025 | 0.02 | 0.25 | 0.14 |

*Note.* LLCI = Lower level confidence interval, ULCI = Upper level confidence interval; all confidence intervals of the pathways are 95% confidence intervals based on 5000 bias-corrected bootstraps. Hetero refers to heterosexual one-night stands, Same-sex refers to same-sex one-night stands. Gender was coded as -1 = female, 1 = male; ONS type was coded as -1 = heterosexual, 1 = same-sex.

**Table S9**

*Serial mediation SEM*

| **Criterion** | **Predictor** | **b** | **SE** | **z** | **p** | **LLCI** | **ULCI** | **β** |
| --- | --- | --- | --- | --- | --- | --- | --- | --- |
| Intoxication (ln) | Gender | -0.46 | 0.09 | -5.38 | < .001 | -0.63 | -0.29 | -0.29 |
| Intoxication (ln) | ONS type | -0.50 | 0.09 | -5.83 | < .001 | -0.67 | -0.33 | -0.17 |
| Intoxication (ln) | Gender × ONS type | -0.22 | 0.09 | -2.57 | .010 | -0.38 | -0.05 | -0.14 |
| Heteronomy | Intoxication (ln) | 0.12 | 0.01 | 8.40 | < .001 | 0.09 | 0.15 | 0.27 |
| Heteronomy | Intoxication (squ) | 0.05 | 0.01 | 4.08 | < .001 | 0.02 | 0.07 | 0.12 |
| Heteronomy | Gender | 0.01 | 0.03 | 0.31 | .758 | -0.06 | 0.07 | 0.01 |
| Heteronomy | ONS type | -0.09 | 0.03 | -2.63 | .008 | -0.16 | -0.02 | -0.07 |
| Heteronomy | Gender × ONS type | 0.09 | 0.03 | 2.61 | .009 | 0.02 | 0.15 | 0.12 |
| Satisfaction | Intoxication (ln) | -0.14 | 0.02 | -6.48 | < .001 | -0.18 | -0.10 | -0.19 |
| Satisfaction | Intoxication (squ) | -0.04 | 0.02 | -2.37 | .018 | -0.07 | -0.01 | -0.07 |
| Satisfaction | Gender | 0.33 | 0.06 | 5.42 | < .001 | 0.21 | 0.45 | 0.29 |
| Satisfaction | ONS type | 0.15 | 0.06 | 2.47 | .014 | 0.03 | 0.28 | 0.07 |
| Satisfaction | Gender × ONS type | -0.13 | 0.06 | -2.25 | .024 | -0.25 | -0.02 | -0.12 |
| Reputational concern | Gender | -0.06 | 0.06 | -0.89 | .372 | -0.19 | 0.06 | -0.05 |
| Reputational concern | ONS type | -0.03 | 0.07 | -0.49 | .623 | -0.15 | 0.11 | -0.01 |
| Reputational concern | Gender × ONS type | 0.12 | 0.06 | 1.85 | .064 | -0.02 | 0.25 | 0.10 |
| Regret | Satisfaction | -0.22 | 0.02 | -9.02 | < .001 | -0.26 | -0.17 | -0.27 |
| Regret | Heteronomy | 0.37 | 0.04 | 9.39 | < .001 | 0.29 | 0.45 | 0.29 |
| Regret | Intoxication (ln) | 0.07 | 0.02 | 4.23 | < .001 | 0.03 | 0.10 | 0.11 |
| Regret | Intoxication (squ) | 0.04 | 0.01 | 3.54 | < .001 | 0.02 | 0.07 | 0.09 |
| Regret | Reputational concern | 0.22 | 0.02 | 9.05 | < .001 | 0.18 | 0.27 | 0.28 |
| Regret | Gender | -0.02 | 0.04 | -0.44 | .663 | -0.09 | 0.06 | -0.02 |
| Regret | ONS type | -0.05 | 0.04 | -1.34 | .180 | -0.13 | 0.02 | -0.03 |
| Regret | Gender × ONS type | 0.02 | 0.04 | 0.40 | .687 | -0.06 | 0.09 | 0.02 |

| **Pathway** | **b** | **SE** | **z** | **p** | **LLCI** | **ULCI** | **β** |
| --- | --- | --- | --- | --- | --- | --- | --- |
| Serial: Gender → Intoxication → Heteronomy → Regret (Hetero) | -0.01 | 0.00 | -3.81 | < .001 | -0.02 | -0.01 | -0.01 |
| Serial: Gender → Intoxication → Heteronomy → Regret (Same-sex) | -0.03 | 0.01 | -3.43 | .001 | -0.05 | -0.02 | -0.03 |
| Serial: Gender → Intoxication → Satisfaction → Regret (Hetero) | -0.01 | 0.00 | -3.56 | < .001 | -0.01 | -0.00 | -0.01 |
| Serial: Gender → Intoxication → Satisfaction → Regret (Same-sex) | -0.02 | 0.01 | -3.26 | .001 | -0.03 | -0.01 | -0.02 |
| Direct: Gender → Intoxication → Regret (Hetero) | -0.02 | 0.01 | -3.21 | .001 | -0.03 | -0.01 | -0.02 |
| Direct: Gender → Intoxication → Regret (Same-sex) | -0.05 | 0.02 | -2.92 | .004 | -0.08 | -0.02 | -0.05 |
| Direct: Gender → Reputational concern → Regret (Hetero) | -0.04 | 0.01 | -4.60 | < .001 | -0.06 | -0.02 | -0.04 |
| Direct: Gender → Reputational concern → Regret (Same-sex) | 0.01 | 0.03 | 0.49 | .621 | -0.05 | 0.07 | 0.01 |
| Direct: Gender → Heteronomy → Regret (Hetero) | -0.03 | 0.01 | -3.34 | .001 | -0.05 | -0.01 | -0.03 |
| Direct: Gender → Heteronomy → Regret (Same-sex) | 0.04 | 0.02 | 1.51 | .132 | -0.01 | 0.08 | 0.04 |
| Direct: Gender → Satisfaction → Regret (Hetero) | -0.10 | 0.01 | -7.40 | < .001 | -0.13 | -0.08 | -0.11 |
| Direct: Gender → Satisfaction → Regret (Same-sex) | -0.04 | 0.03 | -1.63 | .104 | -0.10 | 0.01 | -0.05 |
| Total indirect effect (Hetero) | -0.20 | 0.02 | -9.71 | < .001 | -0.25 | -0.16 | -0.22 |
| Total indirect effect (Same-sex) | -0.09 | 0.06 | -1.47 | .141 | -0.21 | 0.03 | -0.10 |
| Total effect (Hetero) | -0.24 | 0.03 | -8.25 | < .001 | -0.29 | -0.18 | -0.25 |
| Total effect (Same-sex) | -0.09 | 0.09 | -0.99 | .324 | -0.27 | 0.09 | -0.10 |
| Moderated mediation: Intoxication → Heteronomy | -0.02 | 0.01 | -2.36 | .019 | -0.04 | -0.00 | -0.02 |
| Moderated mediation: Intoxication → Satisfaction | -0.01 | 0.01 | -2.30 | .021 | -0.03 | -0.00 | -0.01 |
| Moderated mediation: Intoxication pathway | -0.03 | 0.01 | -2.14 | .032 | -0.06 | -0.01 | -0.03 |
| Moderated mediation: Heteronomy pathway | 0.06 | 0.03 | 2.50 | .013 | 0.01 | 0.11 | 0.07 |
| Moderated mediation: Satisfaction pathway | 0.06 | 0.03 | 2.18 | .029 | 0.01 | 0.11 | 0.06 |
| Moderated mediation: Reputational concern pathway | 0.05 | 0.03 | 1.84 | .066 | -0.01 | 0.11 | 0.06 |
| Moderated mediation: Total index | 0.11 | 0.06 | 1.81 | .070 | -0.01 | 0.24 | 0.12 |

*Note.* LLCI = Lower level confidence interval, ULCI = Upper level confidence interval; all confidence intervals of the pathways are 95% confidence intervals based on 5000 bias-corrected bootstraps. Hetero refers to heterosexual one-night stands, Same-sex refers to same-sex one-night stands. Gender was coded as -1 = female, 1 = male; ONS type was coded as -1 = heterosexual, 1 = same-sex.

**Table S10**

*Satisfaction components SEM*

| **Criterion** | **Predictor** | **b** | **SE** | **z** | **p** | **LLCI** | **ULCI** | **β** |
| --- | --- | --- | --- | --- | --- | --- | --- | --- |
| Regret | Gender | 0.10 | 0.05 | 1.90 | .057 | 0.00 | 0.20 | 0.10 |
| Regret | ONS type | -0.01 | 0.04 | -0.23 | .820 | -0.09 | 0.08 | -0.01 |
| Regret | Gender × ONS type | 0.00 | 0.04 | 0.00 | .999 | -0.08 | 0.09 | 0.00 |
| Sex. pleasure | Gender | 0.26 | 0.07 | 3.80 | < .001 | 0.13 | 0.40 | 0.22 |
| Sex. pleasure | ONS type | 0.20 | 0.07 | 2.92 | .003 | 0.07 | 0.34 | 0.09 |
| Sex. pleasure | Gender × ONS type | -0.01 | 0.07 | -0.21 | .836 | -0.15 | 0.12 | -0.01 |
| Partner competence | Gender | 0.10 | 0.06 | 1.61 | .108 | -0.02 | 0.22 | 0.09 |
| Partner competence | ONS type | 0.17 | 0.06 | 2.78 | .006 | 0.05 | 0.28 | 0.08 |
| Partner competence | Gender × ONS type | -0.08 | 0.06 | -1.36 | .173 | -0.20 | 0.04 | -0.08 |
| Ability to satisfy | Gender | 0.26 | 0.07 | 3.81 | < .001 | 0.13 | 0.40 | 0.21 |
| Ability to satisfy | ONS type | 0.19 | 0.07 | 2.73 | .006 | 0.05 | 0.32 | 0.08 |
| Ability to satisfy | Gender × ONS type | -0.18 | 0.07 | -2.60 | .009 | -0.31 | -0.04 | -0.14 |
| Orgasm | Gender | 0.64 | 0.09 | 6.91 | < .001 | 0.48 | 0.82 | 0.50 |
| Orgasm | ONS type | 0.20 | 0.09 | 2.12 | .034 | 0.03 | 0.38 | 0.08 |
| Orgasm | Gender × ONS type | -0.12 | 0.09 | -1.26 | .208 | -0.27 | 0.07 | -0.09 |
| Heteronomy | Gender | -0.03 | 0.03 | -0.97 | .333 | -0.10 | 0.03 | -0.05 |
| Heteronomy | ONS type | -0.13 | 0.03 | -3.88 | < .001 | -0.20 | -0.07 | -0.10 |
| Heteronomy | Gender × ONS type | 0.07 | 0.04 | 2.02 | .043 | 0.00 | 0.14 | 0.10 |
| Intoxication (ln) | Gender | -0.46 | 0.09 | -5.37 | < .001 | -0.63 | -0.29 | -0.29 |
| Intoxication (ln) | ONS type | -0.50 | 0.09 | -5.82 | < .001 | -0.67 | -0.33 | -0.17 |
| Intoxication (ln) | Gender × ONS type | -0.22 | 0.09 | -2.57 | .010 | -0.38 | -0.05 | -0.14 |
| Intoxication (squ) | Gender | 0.28 | 0.11 | 2.48 | .013 | 0.06 | 0.49 | 0.14 |
| Intoxication (squ) | ONS type | 0.40 | 0.11 | 3.61 | < .001 | 0.19 | 0.62 | 0.11 |
| Intoxication (squ) | Gender × ONS type | 0.26 | 0.11 | 2.33 | .020 | 0.04 | 0.48 | 0.13 |
| Reputational concern | Gender | -0.06 | 0.06 | -0.90 | .370 | -0.19 | 0.06 | -0.05 |
| Reputational concern | ONS type | -0.03 | 0.07 | -0.49 | .624 | -0.15 | 0.11 | -0.01 |
| Reputational concern | Gender × ONS type | 0.12 | 0.06 | 1.85 | .064 | -0.02 | 0.25 | 0.10 |
| Regret | Sex. pleasure | -0.16 | 0.03 | -5.61 | < .001 | -0.21 | -0.10 | -0.20 |
| Regret | Orgasm | -0.22 | 0.04 | -5.79 | < .001 | -0.29 | -0.14 | -0.29 |
| Regret | Partner competence | -0.03 | 0.03 | -0.99 | .322 | -0.09 | 0.03 | -0.03 |
| Regret | Ability to satisfy | -0.07 | 0.03 | -2.15 | .031 | -0.13 | -0.01 | -0.09 |
| Regret | Heteronomy | 0.35 | 0.04 | 8.65 | < .001 | 0.27 | 0.42 | 0.27 |
| Regret | Intoxication (ln) | 0.06 | 0.02 | 4.00 | < .001 | 0.03 | 0.09 | 0.10 |
| Regret | Intoxication (squ) | 0.04 | 0.01 | 3.56 | < .001 | 0.02 | 0.07 | 0.09 |
| Regret | Reputational concern | 0.22 | 0.02 | 8.89 | < .001 | 0.17 | 0.27 | 0.27 |

| **Pathway** | **b** | **SE** | **z** | **p** | **LLCI** | **ULCI** | **β** |
| --- | --- | --- | --- | --- | --- | --- | --- |
| Indirect: Gender → Physical pleasure → Regret (Hetero) | -0.04 | 0.01 | -4.40 | < .001 | -0.06 | -0.03 | -0.05 |
| Indirect: Gender → Orgasm → Regret (Hetero) | -0.16 | 0.03 | -5.43 | < .001 | -0.22 | -0.11 | -0.17 |
| Indirect: Gender → Partner competence → Regret (Hetero) | -0.01 | 0.01 | -0.96 | .339 | -0.02 | 0.01 | -0.01 |
| Indirect: Gender → Ability to satisfy → Regret (Hetero) | -0.03 | 0.01 | -2.09 | .036 | -0.06 | 0.00 | -0.03 |
| Indirect: Gender → Heteronomy → Regret (Hetero) | -0.04 | 0.01 | -4.17 | < .001 | -0.05 | -0.02 | -0.04 |
| Indirect: Gender → Intoxication (ln) → Regret (Hetero) | -0.02 | 0.00 | -3.11 | .002 | -0.03 | -0.01 | -0.02 |
| Indirect: Gender → Intoxication (squ) → Regret (Hetero) | 0.00 | 0.00 | 0.24 | .810 | 0.00 | 0.01 | 0.00 |
| Indirect: Gender → Reputational concern → Regret (Hetero) | -0.04 | 0.01 | -4.59 | < .001 | -0.06 | -0.02 | -0.04 |
| Indirect: Gender → Physical pleasure → Regret (Same-sex) | -0.04 | 0.02 | -1.73 | .083 | -0.09 | 0.00 | -0.04 |
| Indirect: Gender → Orgasm → Regret (Same-sex) | -0.11 | 0.04 | -2.55 | .011 | -0.21 | -0.04 | -0.12 |
| Indirect: Gender → Partner competence → Regret (Same-sex) | 0.00 | 0.01 | -0.09 | .930 | -0.01 | 0.01 | 0.00 |
| Indirect: Gender → Ability to satisfy → Regret (Same-sex) | -0.01 | 0.01 | -0.53 | .596 | -0.03 | 0.01 | -0.01 |
| Indirect: Gender → Samenomy → Regret (Same-sex) | 0.01 | 0.02 | 0.56 | .578 | -0.03 | 0.06 | 0.01 |
| Indirect: Gender → Intoxication (ln) → Regret (Same-sex) | -0.04 | 0.02 | -2.82 | .005 | -0.08 | -0.02 | -0.04 |
| Indirect: Gender → Intoxication (squ) → Regret (Same-sex) | 0.02 | 0.01 | 1.95 | .051 | 0.00 | 0.05 | 0.02 |
| Indirect: Gender → Reputational concern → Regret (Same-sex) | 0.01 | 0.03 | 0.49 | .621 | -0.04 | 0.07 | 0.01 |
| Total indirect effect (Hetero) | -0.33 | 0.04 | -8.01 | < .001 | -0.41 | -0.25 | -0.35 |
| Total indirect effect (Same-sex) | -0.15 | 0.08 | -1.81 | .070 | -0.32 | 0.00 | -0.16 |
| Total effect (Hetero) | -0.23 | 0.03 | -8.05 | < .001 | -0.29 | -0.18 | -0.25 |
| Total effect (Same-sex) | -0.05 | 0.09 | -0.59 | .553 | -0.23 | 0.12 | -0.06 |
| Moderated mediation: Physical pleasure pathway | 0.00 | 0.02 | 0.21 | .837 | -0.04 | 0.05 | 0.00 |
| Moderated mediation: Orgasm pathway | 0.05 | 0.04 | 1.21 | .227 | -0.03 | 0.13 | 0.05 |
| Moderated mediation: Partner competence pathway | 0.01 | 0.01 | 0.68 | .494 | -0.01 | 0.02 | 0.01 |
| Moderated mediation: Ability to satisfy pathway | 0.02 | 0.01 | 1.63 | .103 | 0.00 | 0.06 | 0.02 |
| Moderated mediation: Heteronomy pathway | 0.05 | 0.03 | 1.95 | .051 | 0.00 | 0.10 | 0.05 |
| Moderated mediation: Intoxication (ln) pathway | -0.03 | 0.01 | -2.09 | .036 | -0.06 | -0.01 | -0.03 |
| Moderated mediation: Intoxication (squ) pathway | 0.02 | 0.01 | 1.87 | .062 | 0.00 | 0.05 | 0.02 |
| Moderated mediation: Reputational concern pathway | 0.05 | 0.03 | 1.84 | .065 | -0.01 | 0.11 | 0.06 |
| Moderated mediation: Total index | 0.18 | 0.08 | 2.17 | .030 | 0.01 | 0.34 | 0.19 |

*Note.* LLCI = Lower level confidence interval, ULCI = Upper level confidence interval; all confidence intervals are 95% confidence intervals based on 5000 bias-corrected bootstraps. Hetero refers to heterosexual one-night stands, Same-sex refers to same-sex one-night stands. Gender was coded as -1 = female, 1 = male; ONS type was coded as -1 = heterosexual, 1 = same-sex.

# ONS contexts for students and non-students

**Table S11**

*Frequency of different ONS contexts for students and non-students*

| Context | Student | | Total | Test of proportion equality |
| --- | --- | --- | --- | --- |
|  | 0 | 1 |  |  |
| After a date | 49 15.7 % | 111 15.2 % | 160 15.3 % | χ^2^ = 0.01, *p* = .928 |
| After a party | 92 29.4 % | 395 54.1 % | 487 46.7 % | χ^2^ = 52.78, *p* < .001 |
| After a small social gathering | 53 16.9 % | 91 12.5 % | 144 13.8 % | χ^2^ = 3.31, *p* = .069 |
| After a sex date | 76 24.3 % | 83 11.4 % | 159 15.2 % | χ^2^ = 27.27, *p* < .001 |
| After an unplanned encounter | 38 12.1 % | 44 6 % | 82 7.9 % | χ^2^ = 10.47, *p* = .001 |
| Other | 5 1.6 % | 6 0.8 % | 11 1.1 % | n too small |
| Total | 313 100 % | 730 100 % | 1043 100 % |  |
| *Note.* Student was coded 0 = not a student, 1 = student | | | | |

# Regret distribution for different nationalities

**Figure S18**

*Regret distribution for different nationalities*

**
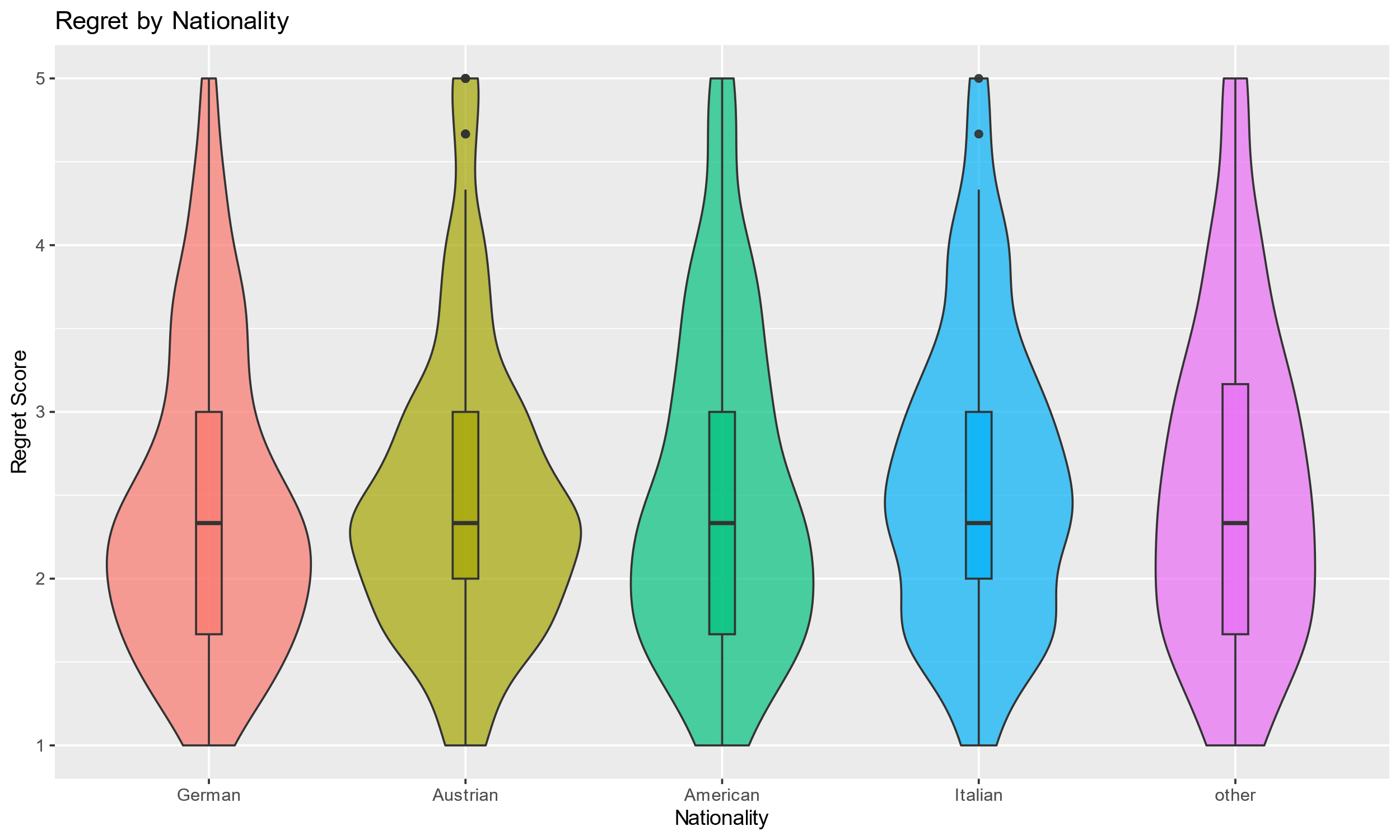
**

1. Zwick, R. (1985). Nonparametric one-way multivariate analysis of variance: A computational approach based on the Pillai-Bartlett trace. *Psychological Bulletin, 97*(1), 148–152. https://doi.org/10.1037/0033-2909.97.1.148 [↑](#footnote-ref-1)
2. Raftery, A. E. (1995). Bayesian model selection in social research. *Sociological Methodology, 25,* 111-163. [↑](#footnote-ref-2)
